# Paramagpy: software for fitting magnetic susceptibility tensors using paramagnetic effects measured in NMR spectra

**DOI:** 10.5194/mr-1-1-2020

**Published:** 2020-02-14

**Authors:** Henry William Orton, Thomas Huber, Gottfried Otting

**Affiliations:** Research School of Chemistry, Australian National University, Canberra, ACT 2601, Australia

## Abstract

Paramagnetic metal ions with fast-relaxing electrons generate pseudocontact shifts (PCSs), residual dipolar couplings (RDCs), paramagnetic relaxation enhancements (PREs) and cross-correlated relaxation (CCR) in the nuclear magnetic resonance (NMR) spectra of the molecules they bind to. These effects offer long-range structural information in molecules equipped with binding sites for such metal ions. Here we present the new open-source software Paramagpy, which has been written in Python 3 with a graphic user interface. Paramagpy combines the functionalities of different currently available programs to support the fitting of magnetic susceptibility tensors using PCS, RDC, PRE and CCR data and molecular coordinates in Protein Data Bank (PDB) format, including a convenient graphical user interface. Paramagpy uses efficient fitting algorithms to avoid local minima and supports corrections to back-calculated PCS and PRE data arising from cross-correlation effects with chemical shift tensors. The source code is available from 10.5281/zenodo.3594568 [Bibr bib1.bibx27].

## 1 Introduction

Paramagnetic metal ions with fast-relaxing electrons produce a number of spatially dependent effects in nuclear magnetic resonance (NMR) spectra of biomolecules which are useful for probing molecular structure and interactions. These effects arise from the magnetic susceptibility of unpaired electrons, which manifests in NMR spectra most notably as pseudocontact shifts (PCSs), paramagnetic relaxation enhancements (PRE) and residual dipolar couplings (RDCs), but also as cross-correlated relaxation (CCR) effects. PCSs and RDCs only arise when the magnetic susceptibility is anisotropic, which is the case for all trivalent paramagnetic lanthanide ions except ${\mathrm{Gd}}^{3+}$ .

A number of programs have been developed for fitting the parameters of magnetic susceptibility tensors, χ , to atomic coordinates of biomolecules using the paramagnetic effects experimentally observed in NMR spectra. The program Numbat supports calculation and fitting of the magnetic susceptibility anisotropy tensor, Δχ , from experimental PCS data with corrections for residual anisotropic chemical shifts (RACSs) [Bibr bib1.bibx19] in a convenient graphical user interface (GUI) [Bibr bib1.bibx34]. The Python module PyParaTools offers similar functionality to Numbat but in a scripting environment and adds methods for fitting χ tensors and alignment tensors using PREs and RDCs, respectively [Bibr bib1.bibx38]. The software FANTEN offers a convenient web-based GUI for fitting Δχ and alignment tensors from PCS and RDC data sets, respectively [Bibr bib1.bibx33].

RDCs arise not only from paramagnetism, but also in the presence of alignment media such as dilute liquid crystals. The programs PALES [Bibr bib1.bibx43] and REDCAT [Bibr bib1.bibx40] fit alignment tensors to atomic coordinates using RDCs. The program Module can use RDCs to fit alignment tensors for molecular structure refinement [Bibr bib1.bibx14]. PCS and RDC restraints have also been implemented in the software packages CYANA [Bibr bib1.bibx1], XPLOR-NIH [Bibr bib1.bibx2], Rosetta [Bibr bib1.bibx35] and HADDOCK [Bibr bib1.bibx13] for structure determination and refinement.

The coordinates of paramagnetic centres can also be determined from PREs, and suitable fitting programs include the programs RelaxGUI [Bibr bib1.bibx9] and Spinach [Bibr bib1.bibx18]. CCR effects can occur between Curie-spin and dipole–dipole relaxation [Bibr bib1.bibx16] and also between Curie-spin and chemical shift anisotropy (CSA) relaxation [Bibr bib1.bibx30]. The former is observed as a difference in relaxation rates between the multiplet components of scalar coupled resonances [Bibr bib1.bibx16]. The software FANTACROSS supports calculation of this CCR effect, but does not allow fitting of the χ tensor position [Bibr bib1.bibx6]. The latter CCR effect was experimentally demonstrated only recently [Bibr bib1.bibx26].

NMR spectra of biomolecules labelled with paramagnetic metal ions with fast electronic relaxation rates, as afforded by lanthanide tags, simultaneously display PCS, RDC, PRE and CCR effects in the same spectrum [Bibr bib1.bibx31]. Due to their common origin in the paramagnetism of the metal ion, all these effects are interrelated. For example, the Δχ tensor determined from PCS measurements can, in principle, be used to predict RDCs, and RDCs arising from paramagnetic alignment allow predictions of some of the Δχ -tensor parameters. The software PyParaTools offers convenient integration of all of these effects, but it lacks many refinements, such as the computation of RACS effects which may affect PCS measurements [Bibr bib1.bibx19], explicit routines for calculating PREs based on Solomon–Bloembergen–Morgan (SBM) or Curie-spin relaxation theory including anisotropic effects arising from non-vanishing Δχ tensors, calculation of cross-correlated Curie-spin–CSA PRE effects or Curie-spin and dipole–dipole CCR involving anisotropic Δχ tensors, or anisotropic SBM [Bibr bib1.bibx39] calculations.

Here we present a new Python-based program, Paramagpy, which offers a graphical interface for fitting magnetic susceptibility tensors using PCS, RDC, PRE and CCR data and seamless transition between these calculations. The fitting routine of Paramagpy for determining Δχ tensors from PCSs employs an efficient grid search algorithm as previously implemented in GPS-Rosetta [Bibr bib1.bibx35]. The algorithm is adept at overcoming the local minima problem that sometimes compromises the results obtained with Numbat and PyParaTools. Paramagpy uses both Curie-spin [Bibr bib1.bibx17] and Solomon–Bloembergen–Morgan [Bibr bib1.bibx37] theory to calculate PREs, and it includes cross-correlation effects with anisotropic chemical shift tensors [Bibr bib1.bibx30], which have not been taken into account by any previous tensor-fitting software. Paramagpy can be installed as a Python module and scripted for efficient calculations, or run via an intuitive GUI.

Calculations using Paramagpy have been verified with data from previous publications. This includes fitting of Δχ tensors to amide PCS data of lanthanide-loaded calbindin D ${}_{\text{9k}}$ and calculating PREs for amide ${}^{1}H$ spins [Bibr bib1.bibx25]. Paramagpy has also been used successfully to predict cross-correlated CSA–Curie-spin relaxation giving rise to negative PREs for amide ${}^{15}N$ spins [Bibr bib1.bibx26]. CCR calculations have been verified with data from high- and low-spin paramagnetic myoglobin [Bibr bib1.bibx29]. Paramagpy has been shown to fit alignment tensors consistent with previous results for lanthanide-tagged ubiquitin [Bibr bib1.bibx28], but may also be applied to data sets arising from alignment media where Paramagpy reports alignment and Saupe tensors alongside Δχ tensors. Paramagpy can thus be used with RDC data obtained by any means of weak molecular alignment in the magnetic field, substituting software like Module [Bibr bib1.bibx14].

## 2 Pseudocontact shifts

The magnetic susceptibility tensor χ associated with a paramagnetic centre creates a dipolar shielding tensor σ at a given position r and distance r from the paramagnetic centre as shown in Eq. (1), where $I_{3}$ is the 3×3 identity matrix, ⊗ denotes the Kronecker product and . denotes the matrix multiplication.

1σ=14π3r⊗rTr5-I3r3.χ2=14πr5(3x2-r2)3xy3xz3xy(3y2-r2)3yz3xz3yz(3z2-r2).χxxχxyχxzχxyχyyχyzχxzχyzχzz

The PCS is given by the trace of the shielding tensor as shown in the PCS Eq. (3). The Δχ tensor is given by the traceless part of the χ tensor. Considering only the Δχ tensor, a linear form of the PCS equation can be obtained, which characterises the Δχ tensor by five explicit parameters as shown in Eq. (4). Including the three position parameters represented by the coordinates of the metal centre ( x , y , z ), solving the PCS equation requires determining eight parameters in total.

3δPCS=13Tr[σ]4=14πr5x2-z2,y2-z2,2xy,2xz,2yz.ΔχxxΔχyyΔχxyΔχxzΔχyz

### 2.1 Singular value decomposition (SVD) grid search

Equation (4) can be rewritten in matrix form to give Eq. (5), where b is a column vector of length n of the calculated PCS values, x is a column vector of length 5 of the Δχ -tensor parameters and A is a n×5 matrix with rows defined by the row vector in Eq. (4) containing coordinate parameters.

5A.x=b6x=A+.b7x=(W.A)+.(W.b)

Populating vector b with many experimental PCS values and the matrix A with atomic coordinates from a molecule of known structure, the system is likely overdetermined and a least-squares solution for the Δχ -tensor parameters x can be obtained analytically by considering the singular values of the matrix A and constructing the pseudo-inverse $A^{+}$ . This allows calculation of the best-fitting tensor at a given position by Eq. (6) [Bibr bib1.bibx35]. A weighted least-squares fit can be obtained using Eq. (7), where the square matrix W contains the weights along the diagonal $W_{ii}=1/S_{\text{PCS},i}$ , which may be sourced from the experimental standard deviations $S_{\text{PCS},i}$ of the i th spin.

Since this calculation is fast, a grid search over many positions of the paramagnetic centre is feasible, providing a robust initial guess prior to iterative refinement of the tensor position by non-linear gradient-descent methods. Paramagpy can evaluate 5000 grid points for 50 PCS values in under 1 s using a 2 GHz Intel i5 2016 processor of a typical laptop computer.

### 2.2 Non-linear gradient descent

When fitting of the position of the paramagnetic centre is required, the PCS equation becomes non-linear. A fit can be found iteratively by minimising the sum of squares of the differences between experimental and back-calculated PCS values. An efficient method for minimisation is by non-linear gradient descent. We chose the Broyden–Fletcher–Goldfarb–Shanno (BFGS) algorithm [Bibr bib1.bibx15] for non-linear least-squares minimisation of the cost function in Eq. ([Disp-formula Ch1.E8]). Here, ${\text{PCS}}_{i}^{\exp}$ and ${\text{PCS}}_{i}^{\mathrm{cal}}$ are, respectively, the experimental and back-calculated PCSs for spin i , and $S_{\text{PCS},i}$ is the experimental uncertainty in the PCS of spin i .

8 $$\text{cost}=\sum_{i}\frac{{\left({\text{PCS}}_{i}^{\mathrm{cal}}-{\text{PCS}}_{i}^{\exp}\right)}^{2}}{S_{\text{PCS},i}^{2}}$$

### 2.3 Multiple PCS data sets

Often there are multiple PCS data sets available for different metal ions bound at the same position, obtained from multiple samples prepared with different metal ions. A simultaneous fit of the common position is possible, independently fitting the tensor magnitude and orientation for each data set, and can lead to a more accurate overall position of the paramagnetic centre. Paramagpy supports multiple data sets for simultaneous fitting of a common metal position by both the SVD grid-search and non-linear gradient-descent algorithms.

### 2.4 Corrections to PCS calculations

An anisotropic magnetic susceptibility causes alignment of the molecule in the external magnetic field. As molecular orientations are no longer sampled uniformly, shielding tensors may no longer average to their isotropic values. In this situation, the chemical shift actually observed in the paramagnetic sample contains contributions from residual anisotropic chemical shifts (RACSs) arising from non-zero averaging of the chemical shift anisotropy (CSA) tensor. Paramagpy supports PCS calculations that include RACS corrections [Bibr bib1.bibx19]. Paramagpy provides standard CSA tensors for amide ${}^{1}$ H spins and backbone amide ${}^{15}$ N and carbonyl ${}^{13}$ C spins [Bibr bib1.bibx11]. Customised CSA tensors may also be set for any of the nuclear spins.

In addition to the CSA tensor, there is also a dipolar shielding tensor σ at the site of a nuclear spin, which arises from the magnetic susceptibility of the paramagnetic centre. In analogy to the RACS effect, this can lead to a residual anisotropic dipolar shift (RADS), which is a small perturbation to the observed PCS in paramagnetic samples arising from molecular alignment [Bibr bib1.bibx8]. Paramagpy includes RADS as an option in the PCS calculation and Δχ -tensor fitting routines.

Systematic errors in experimental PCS values can arise due to variations in the carrier frequency or calibration of the recorded NMR spectra of the diamagnetic and paramagnetic species. This offset can be included as a parameter during the fitting of Δχ tensors, although doing so is meaningful only if a sufficient number of PCS data are available to avoid overfitting.

## 3 Residual dipolar couplings

An anisotropic magnetic susceptibility tensor induces a coincident alignment tensor A , giving rise to RDCs between nuclear spins. The alignment tensor can be found from the Δχ tensor using Eq. ([Disp-formula Ch1.E9]), where $B_{0}$ is the magnetic field, $\mu_{0}$ the vacuum permeability, $k_{B}$ the Boltzmann constant and T the temperature [Bibr bib1.bibx8].

9 $$A=\frac{B_{0}^{2}}{15\mu_{0}k_{B}T}\Delta \chi$$

The RDC values can be calculated using Eq. (10), where $r_{AB}$ is the internuclear vector and $r_{AB}$ the distance between the two nuclei A and B [Bibr bib1.bibx22]. This can be expanded into the vector equation Eq. (11), where x , y and z are the Cartesian coordinates of the internuclear vector $r_{AB}$ .

10RDC=3γAγBμ0ℏ8π2rAB5rABT.A.rAB11=3γAγBμ0ℏ8π2rAB5x2-z2,y2-z2,2xy,2xz,2yz.AxxAyyAxyAxzAyz

Unlike the PCS tensor, the RDC tensor does not require parameters for position and can therefore be described by five parameters for magnitude and orientation. Fitting can therefore be achieved by a linear least-squares fit.

### 3.1 SVD fitting algorithm

Paramagpy uses the SVD algorithm similar to the original implementation in the program REDCAT [Bibr bib1.bibx40]. It is functionally the same as the algorithm applied to solving the PCS equation in Sect. [Sec Ch1.S2.SS1]. A n×5 matrix with rows defined by the row vector in Eq. (11) containing coordinate parameters is constructed. From this, a pseudo-inverse matrix is calculated and applied to the experimental RDC values, yielding the best-fitting alignment tensor.

## 4 Paramagnetic relaxation enhancements

PREs describe the relaxation rates of longitudinal magnetisation, $R_{1}=1/T_{1}$ , or transverse magnetisation, $R_{2}=1/T_{2}$ , of nuclear spins, where $T_{1}$ and $T_{2}$ are the longitudinal and transverse relaxation times, respectively. For PREs of paramagnetic molecules in solution, the relaxation rates are governed by dipole–dipole interactions as described by the SBM equations or the shielding tensor anisotropy as described by the Curie-spin equations [Bibr bib1.bibx37].

### 4.1 Solomon–Bloembergen–Morgan theory

The SBM equations for $R_{1}$ and $R_{2}$ are shown in Eqs. (12) and (13), respectively, where γ is the nuclear gyromagnetic ratio, r the distance of the nucleus from the paramagnetic centre, and ω and $\omega_{S}$ the nuclear and electronic Larmor frequencies, respectively. $\tau_{c}$ is the correlation time calculated as $1/\tau_{c}=1/\tau_{r}+1/T_{1e}$ , where $\tau_{r}$ is the rotational correlation time of the molecule and $T_{1e}$ is the electronic relaxation time. $\mu_{\text{eff}}$ is the effective magnetic moment of the paramagnetic centre, which can be predicted from the Landé g factor, the Bohr magneton $\mu_{\text{B}}$ and the total angular momentum quantum number J (Eq. 14).

12R1SBM=215μ04πγμeffr323τc1+τc2ω2+7τc1+τc2ωS213R2SBM=115μ04πγμeffr324τc+3τc1+τc2ω2+13τc1+τc2ωS214μeff=gμBJ(J+1)

An extension to the SBM theory which accounts for anisotropy of the dipolar spectral density is described by Eqs. (15) and (16) where G(ω) describes the spectral power density tensor [Bibr bib1.bibx39]. $\hat{r}$ is the unit vector from the paramagnetic centre to the nuclear spin. The spectral power density tensor usually cannot be derived theoretically, but is instead fitted to experimental data.

15R1SBM-aniso=23μ04πγr32Tr3r^⊗r^-I32.G(ω)16R2SBM-aniso=13μ04πγr32Tr3r^⊗r^-I32.(G(0)+G(ω))

### 4.2 Curie-spin theory

Curie-spin relaxation is governed by the dipolar shielding tensor σ as calculated in Eq. (1), which must include the isotropic component of the χ tensor, $\chi_{\text{iso}}$ , which can be predicted using Eq. (17). The first invariant Λ and second invariant Δ of the shielding tensor are calculated by Eqs. (18) and (19), where $\sigma_{ij}$ denotes the i th and j th components of the shielding tensor σ [Bibr bib1.bibx39]. This allows calculation of the $R_{1}$ and $R_{2}$ PREs by Eqs. (20) and (21), respectively. These equations account for anisotropy of the magnetic susceptibility, provided Eq. (1) is used to calculate σ [Bibr bib1.bibx41].

17χiso=μ0μeff23kBT18Λ2=(σxy-σyx)2+(σxz-σzx)2+(σyz-σzy)219Δ2=σxx2+σyy2+σzz2-σxxσyy-σxxσzz-σyyσzz+34(σxy+σyx)2+(σxz+σzx)2+(σyz+σzy)220R1Curie=12Λ2ω2τr1+9τr2ω2+215Δ2ω2τr1+ω2τr221R2Curie=14Λ2ω2τr1+9τr2ω2+145Δ2ω24τr+3τr1+τr2ω2

When PREs due to Curie-spin relaxation are cross-correlated with CSA relaxation, the CSA tensor is added to the dipolar shielding tensor to give an effective shielding tensor $\sigma_{\text{eff}}$ . The PRE including CSA cross-correlation $R_{\text{Curie}\;\times \;\text{CSA}}$ is determined as the difference in relaxation rates in the paramagnetic and diamagnetic state as shown in Eq. (22). This can give rise to negative PREs as shown previously and confirmed by experiment [Bibr bib1.bibx30].

22 $$R^{\text{Curie}\;\times \;\text{CSA}}=R^{\mathrm{Curie}}(\sigma_{\text{eff}})-R^{\mathrm{Curie}}(\sigma_{\text{CSA}})$$

### 4.3 Fitting algorithm

Paramagpy includes routines to calculate PREs and fit all parameters for each of the above relaxation theories, including cross-correlated relaxation with CSA effects. This is achieved by non-linear gradient descent to minimise the cost function of Eq. (23). Here, ${\text{PRE}}_{i}^{\exp}$ and ${\text{PRE}}_{i}^{\mathrm{cal}}$ are, respectively, the experimental and back-calculated PREs for spin i , and $S_{\text{PRE},i}$ is the experimental uncertainty in the PRE of spin i . The user can choose to fit or constrain different parameters, such as the magnetic susceptibility or power spectral density tensor position, magnitude, correlation time $\tau_{c}$ , etc. Parameter templates for lanthanide ions are also provided, based on tensor magnitudes and anisotropies previously reported for lanthanide complexes of calbindin D ${}_{\text{9k}}$ [Bibr bib1.bibx5]. These may be used to give a quick estimate of expected PRE values.

23 $$\text{cost}=\sum_{i}\frac{({\text{PRE}}_{i}^{\mathrm{cal}}-{\text{PRE}}_{i}^{\exp}{)}^{2}}{S_{\text{PRE},i}^{2}}$$

## 5 Curie-spin dipole–dipole cross-correlated relaxation

Interference of the internuclear dipole–dipole (DD) relaxation with Curie-spin relaxation provides a mechanism for differential relaxation rates of multiplet components by cross-correlated relaxation (CCR) [Bibr bib1.bibx16]. This effect is readily observed and measured as the difference in the relaxation rate $R_{2}$ of the two doublet components of an amide ${}^{1}H{-}^{15}N$ spin pair. In this case, the shielding tensor arising at the ${}^{1}H$ spin due to the ${}^{15}N$ dipole is given by Eq. (24), where $r_{\text{HN}}$ is the H-N bond vector, $r_{\text{HN}}$ is the internuclear distance, $\gamma_{\text{N}}$ is the gyromagnetic ratio of ${}^{15}N$ and $I=\frac{1}{2}$ is the spin of ${}^{15}N$ . The factor of $1/B_{0}$ is necessary to express the ${}^{15}N$ shielding tensor in units of parts per million to match the units of the Curie-spin shielding tensor. The effective shielding tensor for the ${}^{1}H$ spin due to both the Curie spin and the ${}^{15}N$ dipole in either the up or down spin state is given by Eqs. (25) and (26), respectively. The relaxation rate $R_{2}^{\mathrm{Curie}}$ is then calculated using Eq. (21) for both the up and down effective shielding tensors $\sigma_{↑}$ and $\sigma_{↓}$ , and their difference is taken to represent the Curie×DD differential line broadening $R^{\text{Curie}\;\times \;\text{DD}}$ . In this way the auto-correlated relaxation mechanisms arising from the separate DD and Curie mechanisms are subtracted out, leaving the pure cross-correlated term. A derivation showing the equivalence of Eqs. (24)–(27) to those reported by Ghose and Prestegard [Bibr bib1.bibx16] is given in the Supplement.

24σN=1B0μ04πγNℏI3rHN⊗rHNTrHN5-I3rHN325σ↑=σ+σN26σ↓=σ-σN27RCurie×DD=RCurie(σ↑)-RCurie(σ↓)

Paramagpy uses the above equations for all DD×Curie relaxation calculations. By using Eq. (1) for calculating the Curie-spin shielding tensor σ , these equations also account for anisotropy of the magnetic susceptibility χ . CCR values can be calculated between any two atoms in the specified Protein Data Bank (PDB) file. The calculations have been shown to agree with previous experimental CCR data on high- and low-spin myoglobin [Bibr bib1.bibx29].

### 5.1 Fitting algorithm

Paramagpy includes routines to fit all parameters of the χ tensor, including position, magnitude and anisotropy, to experimentally measured CCR data. This is achieved by non-linear gradient descent to minimise the cost function of Eq. (28). Here, ${\text{CCR}}_{i}^{\exp}$ and ${\text{CCR}}_{i}^{\mathrm{cal}}$ are, respectively, the experimental and back-calculated CCRs for spin i , and $S_{\text{CCR},i}$ is the experimental uncertainty in the CCR of spin i .

28 $$\text{cost}=\sum_{i}\frac{({\text{CCR}}_{i}^{\mathrm{cal}}-{\text{CCR}}_{i}^{\exp}{)}^{2}}{S_{\text{CCR},i}^{2}}$$

## 6 Uncertainty calculations

To judge the quality of a Δχ or χ tensor fitted using PCS, RDC, PRE or CCR data, Paramagpy offers three methods to test the robustness of the fit: structure-sourced, bootstrap and Monte Carlo. The structure-sourced method assumes that multiple models in a PDB file represent experimental uncertainty in the atomic coordinates as is common for NMR structures (see Sect. [Sec Ch1.S7.SS1]). In this approach, a tensor is fitted to each individual model and uncertainties in the fitted tensor parameters are reported. The alternative bootstrapping method repeats the fit many times, with each iteration randomly sampling a specified proportion of the data, and subsequently reports the standard deviation in the fitted tensor parameters. The Monte Carlo method repeats the fit using all the data, but each time adds noise to the experimental values. The noise is sourced from a uniform distribution that has been scaled by values provided by the user for each atom. These scaling values are ideally calculated from noise in the spectrum to reflect uncertainty in peak positions or amplitudes [Bibr bib1.bibx21]. The standard deviations in the fitted tensor parameters are then reported.

## 7 Molecular structures with multiple models

### 7.1 Structures with uncertainties represented by a family of models

Biomolecular structures in the PDB, which have been determined by solution NMR, usually report experimental uncertainty in the atomic coordinates by including multiple models, which individually fulfil the experimental restraints. The default behaviour of Paramagpy is to fit a magnetic susceptibility tensor to each model independently and then report an average of all these tensors. The tensor averaging is achieved by Eq. (29) where the summation runs over the tensors fitted to each of the n models. This ensures no errors are introduced by averaging prolate/oblate tensors with different principal axis definitions. All other parameters involved in the fit, such as origin of the tensor position, rotational correlation time or electronic relaxation time, are averaged in the conventional way. Note that the final result is sensitive to different relative orientations of the models.

29 $$\chi_{\text{average}}=\frac{1}{n}\sum_{i}^{n}\left[\begin{array}{ccc} (\chi_{xx}{)}_{i} & (\chi_{xy}{)}_{i} & (\chi_{xz}{)}_{i}\\ (\chi_{xy}{)}_{i} & (\chi_{yy}{)}_{i} & (\chi_{yz}{)}_{i}\\ (\chi_{xz}{)}_{i} & (\chi_{yz}{)}_{i} & (\chi_{zz}{)}_{i} \end{array}\right]$$

### 7.2 Structures represented by a conformational ensemble

Some coordinate sets in the PDB have been determined by molecular dynamics, where the ensemble of models deposited fulfils the experimental restraints better than each individual model. For this case, Paramagpy has the option for calculation of ensemble-averaged paramagnetic effects at all stages of calculations and fitting. Ensemble-averaged fitting presents a subtle but important difference compared to the multiple-model method described in Sect. [Sec Ch1.S7.SS1] above. This is particularly noticeable for RDCs, where the ensemble average can be much smaller than the corresponding RDC of a single model, and therefore several models representing different bond orientations may be simultaneously required to fit an appropriate alignment tensor or Δχ tensor.

The implementation of ensemble averaging in Paramagpy averages the paramagnetic values calculated for each atom in the different models, identifying the specific atoms by identical atom numbers in the PDB file. Custom ensemble averaging behaviour can be changed by the user in the scripted environment. In the implementations of the SVD algorithm, ensemble averaging involves summation of rows for common atoms of the matrix A of Eq. (5) before calculation of the singular values. In the implementations of the non-linear gradient descent algorithm, the values calculated for the common atoms are averaged prior to calculating the sum of squares of differences. This is shown in Eq. (30) where $a^{\mathrm{cal}}$ and $a^{\exp}$ are the calculated and experimental PCS, RDC, PRE or CCR values, respectively. The index m is for atoms that are common between models, and the index i runs over all atoms in the structure.

30 $${\text{cost}}_{\text{ensemble}}=\sum_{i}\frac{(\sum_{m}\left[{\text{a}}_{m,i}^{\mathrm{cal}}-{\text{a}}_{i}^{\exp}\right]{)}^{2}}{\sigma_{\text{a(i)}}^{2}}$$

### 7.3 Fitting tensor parameters to multimers

In the case of symmetric multimers composed of monomers with each containing a paramagnetic metal ion, the ensemble averaging feature of Paramagpy can be exploited to fit the Δχ tensor associated with a given monomer. This is achieved simply by defining the monomeric units in the PDB file as models of the same structure and applying the ensemble averaging routine to fit the Δχ tensor using the experimental PCSs, which reflect the average of the PCSs observed in each monomer. Note that, due to the averaging, the final fitted Δχ tensor must be scaled by the user n -fold, where n is the number of monomers. This feature can also be exploited in NMR crystallography [Bibr bib1.bibx20].

## 8 Quality factors

To judge the agreement of tensor fits with the experimental data, a Q factor can be assigned to a given fit, which Paramagpy calculates using Eq. (31). Here, the experimental and calculated PCS, RDC, PRE or CCR values are denoted $a^{\exp}$ and $a^{\mathrm{cal}}$ , respectively, the index m is for ensemble averaging of common spins between models, and the index i is for summation over all spins of the molecule. A low Q factor signifies a good-quality fit.

31 $$Q=\sqrt{\frac{\sum_{i}\left[{\left(\sum_{m}\left[a_{i}^{\exp}-a_{m,i}^{\mathrm{cal}}\right]\right)}^{2}\right]}{\sum_{i}\left[{\left(\sum_{m}\left[a_{i}^{\exp}\right]\right)}^{2}\right]}}$$

Alternative Q factors have been proposed [Bibr bib1.bibx10]. The Q factor proposed by [Bibr bib1.bibx3], which uses sums of experimental and calculated values in the denominator of Eq. (31) and therefore tends to be 2 times smaller, is supported by the scripted environment of Paramagpy. It is important to note that the fitting algorithm used by Paramagpy targets the minimal root-mean-square deviation between experimental and calculated data rather than the Q factor. It has been pointed out that Q -factor evaluations are meaningful only if the number of fitted data greatly exceeds the number of variables [Bibr bib1.bibx4].

## 9 Graphical user interface

Paramagpy has a graphical user interface (GUI) written for the inbuilt Tk/Tcl interface of Python 3, which can run on Mac OS X, Windows and Linux operating systems. The GUI offers a user-friendly environment for loading and visualising PDB files and experimental PCS, RDC, PRE and CCR data. Two frames display the initial and fitted tensors. The fitted tensor is calculated and displayed by the push of a button. An overview of the PCS fitting tab is shown in Fig. [Fig Ch1.F1]. Hovering the mouse over any element in the window displays a useful tool tip to help the user.

**Figure 1 Ch1.F1:**
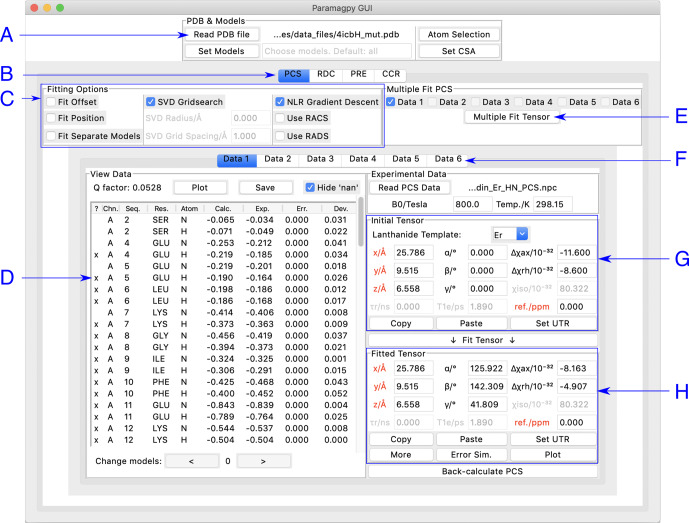
Paramagpy GUI running on Mac OS X. (A) Frame for loading PDB coordinates. The atoms and models (conformers) of interest can be selected and CSA-tensor parameters set by the user. (B) The user can switch between PCS, RDC, PRE and CCR tabs, where CCR stands for the Curie-spin–dipole–dipole cross-correlated relaxation. (C) Fitting options can be specified by selecting the relevant check box. The “SVD Gridsearch” option searches for the best-fit tensor within a sphere about the initial tensor origin with radius and grid spacing as specified. The “NLR Gradient Descent” option refines the tensor using non-linear least-squares minimisation. (D) Experimental data for atoms in the PDB file are displayed here. The first column contains an “x” if the datum will be used during fitting and may be toggled by pressing the “x” key on the keyboard. Experimental and back-calculated PCS values are also reported and their correlation can be displayed by clicking the “Plot” button above. (E) To utilise multiple PCS data sets to fit different tensors to a common position, the “Multiple Fit Tensor” button can be clicked after selecting the desired data sets. (F) Each tab can contain a different PCS data set, allowing up to 6 to be loaded. If more data sets are required, Paramagpy supports this through the scripted module. (G) The initial tensor parameters can be specified here to define a starting point before fitting. For convenience, the paramagnetic centre can be positioned at any atom in the PDB file by double-clicking on a row of the data view in the frame to the left. Parameters in red are constrained during fitting. Greyed out parameters are not relevant to PCS or RDC calculations, but are used in PRE and CCR calculations. (H) The fitted tensor is displayed here. Clicking the “Copy” button allows the tensor to be pasted into other tabs of the program (see B and F above). The “Plot” button will prompt the user to save an isosurface file for opening in PyMOL. “Error Sim.” will assess the quality of the fit by bootstrap or Monte Carlo methods. The button “Set UTR” is for conversion of the tensor parameters to the unique tensor representation defined by the program Numbat [Bibr bib1.bibx34].

## 10 Visualisation

Paramagpy offers a number of plot options to quickly visualise tensors and quality of fit. The scalar PCS or PRE field can be written to a CCP4 [Bibr bib1.bibx24] density map, which can then be visualised as a three-dimensional contour plot in the program PyMOL [Bibr bib1.bibx36]. The fit quality can be visualised in correlation plots of back-calculated PCS, RDC, PRE and CCR values versus the experimental values. Finally, a scatter plot of the principle axes of the tensors can be viewed in a Sanson–Flamsteed-style projection following Monte Carlo or bootstrap error analyses. Example plots are summarised in Fig. [Fig Ch1.F2].

**Figure 2 Ch1.F2:**
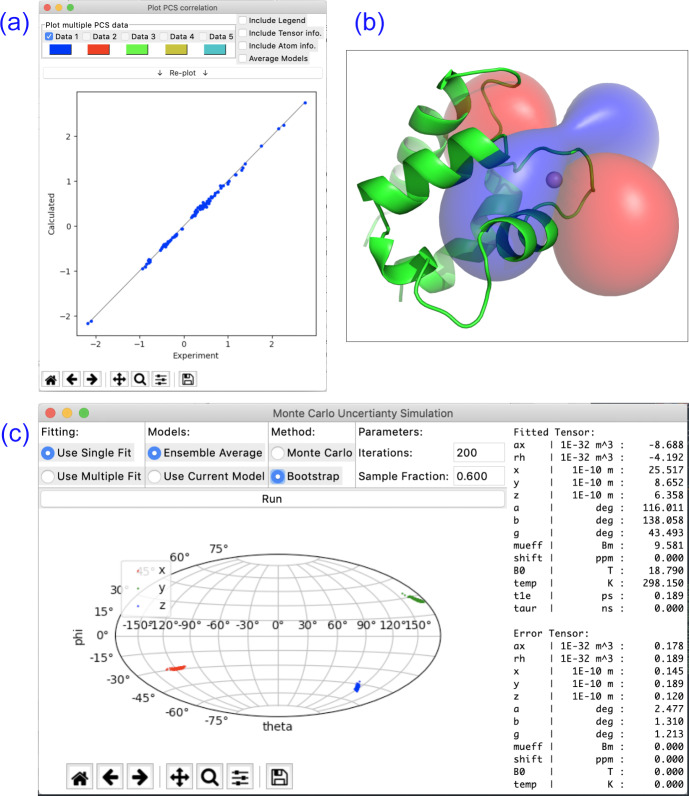
Plotting options available in Paramagpy illustrated with data of calbindin D ${}_{\text{9k}}$ loaded with Er ${}^{3+}$ . **(a)** Correlation plot of calculated versus experimental PCS values after fitting of the Δχ tensor. **(b)** PCS isosurface plot viewed in PyMOL. **(c)** Sanson–Flamsteed plot showing the principle axes projections after bootstrap analysis. “Error Tensor” reports the standard deviation in fitted parameters.

**Figure 3 Ch1.F3:**
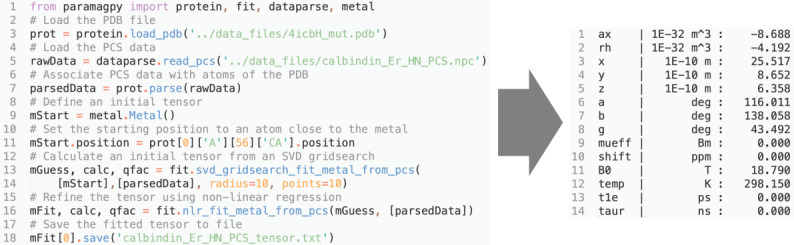
Example Python script for fitting a Δχ tensor to experimental PCS data. The output with fitted tensor parameters is displayed to the right.

**Figure 4 Ch1.F4:**
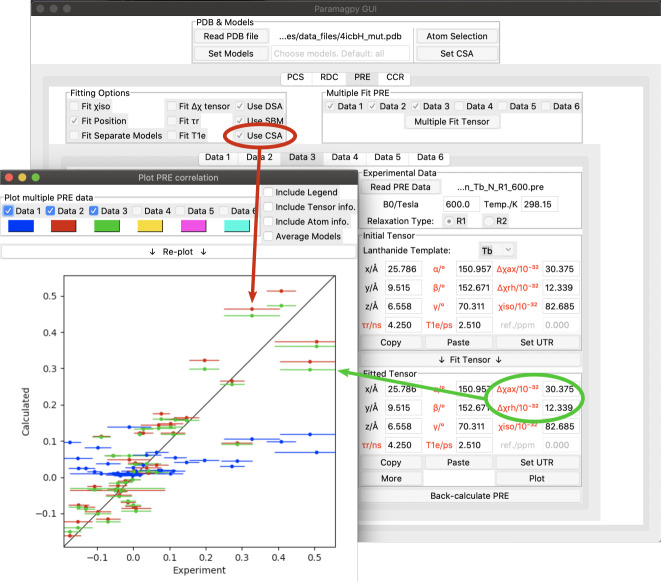
Paramagpy GUI showing $R_{1}$ ( ${}^{15}N$ ) PRE data for calbindin D ${}_{\text{9k}}$ loaded with ${\mathrm{Tb}}^{3+}$ . The correlation plot shows calculated vs. experimental values. Blue: SBM and isotropic Curie-spin theory are used for calculating PREs ( Q factor 1.01). Red: also taking into account the cross-correlation between Curie-spin and CSA relaxation ( Q factor 0.49). Green: including the additional correction arising from the anisotropy of the χ tensor ( Q factor 0.47).

## 11 Scripting

Paramagpy is a Python module and can be imported into a scripting environment. The module is split into four major submodules. (i) The “metal” submodule deals with the paramagnetic centre, tensor representations and methods for calculating PCS, RDC, PRE and CCR values. (ii) The “protein” submodule handles the atomic coordinates from the PDB file and CSA-tensor definitions. (iii) The “dataparse” submodule manages the reading and writing of data files. (iv) The “fit” submodule contains functions for fitting tensors to experimental data. An example script for fitting of a Δχ tensor to experimental PCS data for calbindin D ${}_{\text{9k}}$ is shown in Fig. [Fig Ch1.F3]. It uses only nine lines of code. Some more advanced features of Paramagpy, such as fitting of power spectral density tensors in Eqs. (15) and (16), are only available in the scripted environment. The scripted environment also offers control over which parameters are included for fitting routines and allows calculations for coordinates other than PDB formats.

## 12 NMR software integration

Paramagpy includes macro scripts to interface with popular NMR software: CcpNmr analysis and Sparky [Bibr bib1.bibx42]. Currently, these macros allow for the rapid calculation of experimental PCS values from NMR spectra with up to three dimensions, fitting of Δχ tensors and plotting of back-calculated PCS values onto paramagnetic spectra.

## 13 Tensor conventions and conversions

Paramagpy offers a number of simple routines to convert between tensor representations. In addition to the 3×3 matrix representations of tensors, positions, rotation matrices, eigenvalues, axial/rhombic components and Euler angles, alignment tensors and Saupe tensors are available upon clicking the “More” button within the GUI. The axial and rhombic components are defined as follows (Eqs. 32 and 33).

32Δχax=Δχzz-Δχxx+Δχyy233Δχrh=Δχxx-Δχyy

By default, Paramagpy reports all fitted tensors in the unique tensor representation used by the program Numbat [Bibr bib1.bibx34]. This requires that the principle axis magnitudes of the Δχ tensor are ordered $\left|\Delta \chi_{zz}|\geq |\Delta \chi_{yy}|\geq |\Delta \chi_{xx}\right|$ , and all Euler angles are in the range [0,π] using the ZYZ convention.

## 14 Example PRE calculation

PRE calculations that include anisotropy effects and cross-correlation with CSA can be daunting to set up as they require the Δχ and CSA tensors to possess the correct orientations in the frame of the molecular coordinates. Paramagpy simplifies this for the user by allowing Δχ tensors fitted from PCS data to be transferred easily to the tab for PRE calculations. Furthermore, CSA-tensor templates are provided for most protein backbone atoms.

As an example, Fig. [Fig Ch1.F4] shows the Paramagpy GUI with $R_{1}$ ( ${}^{15}N$ ) PRE data for calbindin D ${}_{\text{9k}}$ loaded with ${\mathrm{Tb}}^{3+}$ [Bibr bib1.bibx26]. A Δχ tensor was fitted using the PCS tab, then transferred to the PRE tab using the “Copy” and “Paste” buttons. Curie-spin–CSA cross-correlation is taken into account simply by checking the box “Use CSA”. This greatly improves the correlation and allows the prediction of negative PREs, resulting in a reduction in the Q factor from 1.01 to 0.49. The small additional correction arising from the anisotropy of the Curie spin can be included by setting the $\Delta \chi_{\text{ax}}$ and $\Delta \chi_{\text{rh}}$ parameters to the non-zero values obtained from the Δχ tensor fitted with the help of PCS data yielding a further reduction in the Q factor to 0.47.

The CSA tensors of ${}^{15}N$ spins are much larger than those of ${}^{1}H$ spins, so that Curie-spin–CSA cross-correlation effects can dominate the PRE to the point that even negative PREs can be observed ([Bibr bib1.bibx26] Fig. 4). These CCR effects are predicted to be most pronounced for ${}^{15}N$ spins located about 10 Å from the metal ion. In contrast, the CSA of ${}^{1}H$ spins is much smaller, so that their CCR effects are predicted to be most significant in the range of 20–25 Å and therefore too small to be easily observed experimentally [Bibr bib1.bibx30].

## 15 Conclusions

Paramagpy is an easy-to-use program that integrates the related paramagnetic NMR phenomena of PCS, RDC, PRE and CCR. Paramagpy allows the rapid analysis of NMR spectra of samples containing a single paramagnetic centre, which is particularly useful for data recorded with different paramagnetic lanthanide ions. With an intuitive calculation flow, Paramagpy can be used, for example, to fit a Δχ tensor using experimental PCS data and then quickly report the expected PREs of the same complex, informing the user which signals may be too broad to observe. Paramagpy uses efficient fitting algorithms and an up-to-date implementation of paramagnetic NMR theory to capture subtle corrections arising from CSA and anisotropy effects in the PCS and PRE calculations.

## Supplement

10.5194/mr-1-1-2020-supplement The supplement related to this article is available online at: https://doi.org/10.5194/mr-1-1-2020-supplement.

## Code availability

The source code for Paramagpy is available at: https://doi.org/10.5281/zenodo.3594568 (Orton, 2019).
