# Supplementary material for: Paramagpy: software for fitting magnetic susceptibility tensors using paramagnetic effects measured in NMR spectra
Source: Magn Reson (Gott). 2020 Feb 14;1(1):1–12. doi: 10.5194/mr-1-1-2020 (PMC10500712; doi:10.5194/mr-1-1-2020)
Supplement: The supplement related to this article is available online at: https://doi.org/10.5194/mr-1-1-2020-supplement. [file mr-1-1-supplement.pdf]

Supplement of Magn. Reson., 1, 1–12, 2020
https://doi.org/10.5194/mr-1-1-2020-supplement
© Author(s) 2020. This work is distributed under
the Creative Commons Attribution 4.0 License.

Open Access
MAGNETIC RESONANCE

Supplement of

Paramagpy: software for fitting magnetic susceptibility tensors using paramagnetic effects measured in NMR spectra

Henry William Orton et al.

Correspondence to: Gottfried Otting (gottfried.otting@anu.edu.au)

The copyright of individual parts of the supplement might differ from the CC BY 4.0 License.

paramagpy Documentation

Release 1.0

Henry Orton

Dec 29, 2019

CONTENTS

1 Introduction **2**

2 Features **3**

3 Documentation **4**

4 Citing paramagpy **5**

5 Contents **6**

5.1 Installation Guide 6
5.2 Examples 7
5.3 Graphic User Interface (GUI) 40
5.4 NMR Software Macros 41
5.5 Mathematical Proofs 44
5.6 Reference Guide 46

Python Module Index **96**

Index **97**

Release 1.0

Date Dec 29, 2019

CHAPTER ONE

INTRODUCTION

paramagpy is a python module for calculating paramagnetic effects in NMR spectra of proteins. This currently includes fitting of paramagnetic susceptibility tensors to experimental data associated with pseudocontact shifts (PCS) residual dipolar couplings (RDC), paramagnetic relaxation enhancements (PRE) and cross-correlated relaxation (CCR). A GUI allows easy viewing of data and seamless transition between PCS/RDC/PRE/CCR calculations.

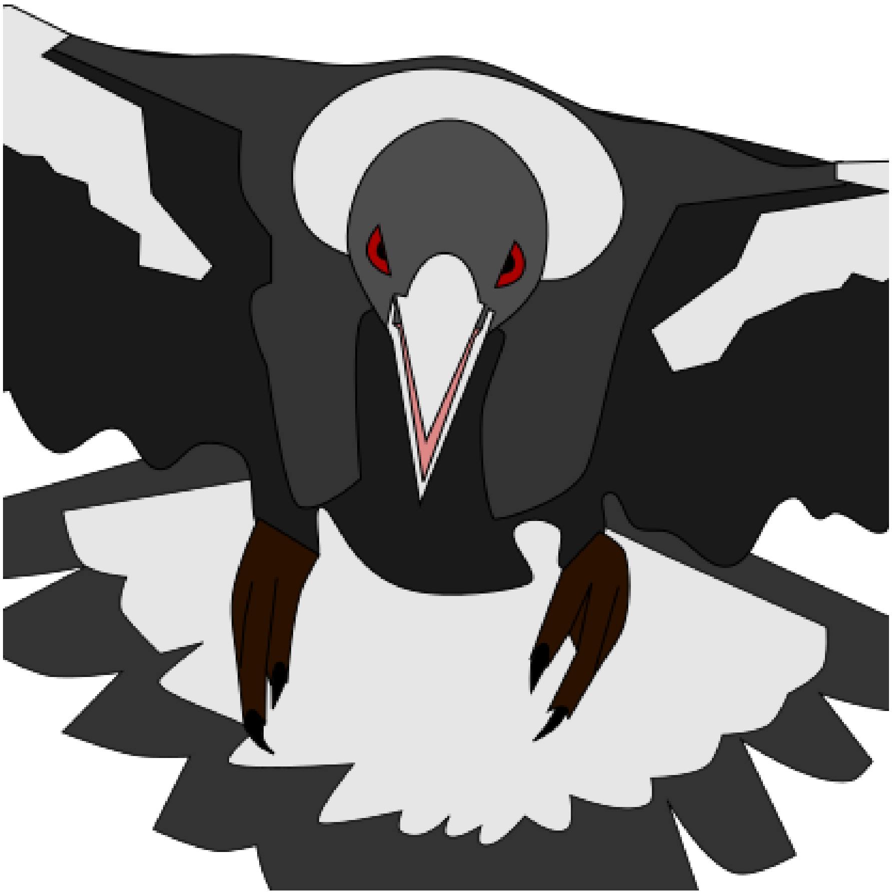

Fig. 1: *Please, not the eyes!* - Canberra cyclist

CHAPTER

TWO

FEATURES

- Support for PDB protein structures with models
- Combined SVD gridsearch and gradient descent algorithms for solving PCS tensors
- Optional fitting of reference offset parameter for PCS datasets
- Support for Residual Anisotropic Chemical Shielding (RACS) and Residual Anisotropic Dipolar Shielding (RADS) corrections to PCS
- Lanthanide parameter templates available
- Plotting of correlation between experiment/calculated values
- Plotting of tensor isosurfaces compatible with PyMol
- Q-factor calculations
- Error analysis of tensor fit quality by Monte-Carlo or Bootstrap methods
- Optimisation of multiple PCS/PRE/CCR datasets to a common position
- Unique tensor representation compatible with Numbat (program)
- Fitting of RDC tensor by SVD algorithm
- PRE calculations by Solomon and Curie spin mechanisms
- Spectral power density tensor fitting for anisotropic dipolar PREs
- CSA cross-correlation correction to PRE calculations
- Dipole-dipole/Curie spin cross-correlated relaxation calculations
- Fitting of tensor parameters to PRE/CCR data
- Macro scripts for integration with CCPNMR and Sparky

CHAPTER

THREE

DOCUMENTATION

- https://henryorton.github.io/paramagpy/

CHAPTER

FOUR

CITING PARAMAGPY

Paramagpy is published in Magnetic Resonance https://doi.org/10.5194/mr-2019-3

CHAPTER

FIVE

CONTENTS

5.1 Installation Guide

5.1.1 Requirements

Paramagpy is written for python 3. It requires packages:

- NumPy
- SciPy
- matplotlib
- BioPython

5.1.2 Unix/OSX Installation

Install directly using pip:

```
$ pip install paramagpy
```

Or, download the source code and run:

```
$ python setup.py install
```

within the source directory.

5.1.3 Windows Installation

Paramagpy has never been tested on windows, but theoretically it should work. Good luck!

5.1.4 Running the GUI

Once you have installed paramagpy, see *Graphic User Interface (GUI)* for how to run the GUI.

5.2 Examples

Note that all these examples are for scripted calculations using the Paramagpy python module. Most of this functionality is also available by the GUI *Graphic User Interface (GUI)*

5.2.1 PCS data

Fit Tensor to PCS Data

This example shows how to fit a $\Delta\chi$-tensor to experimental PCS data for the protein calbindin D9k. These data contain amide 1H and 15N chemical shifts between diamagnetic and paramagnetic states with the lanthanide Er3+ bound.

Downloads

- Download the data files `4icbH_mut.pdb` and `calbindin_Er_HN_PCS.npc` from here:
- Download the script `pcs_fit.py`

Script + Explanation

Firstly, the necessary modules are imported from paramagpy.

```
from paramagpy import protein, fit, dataparse, metal
```

The protein is then loaded from a PDB file using *paramagpy.protein.load_pdb()* into the variable `prot`. This returns a `CustomStructure` object which is closely based on the `Structure` object from BioPython and contains the atomic coordinates. The object, and how to access atomic coordinates is discussed at this link.

```
# Load the PDB file
prot = protein.load_pdb('../data_files/4icbH_mut.pdb')
```

The PCS data is then loaded from a `.npc` file using the function *paramagpy.dataparse.read_pcs()* into the variable `rawData`. This is a dictionary of `(PCS, Error)` tuples which may be accessed by `rawData[(seq, atom)]` where `seq` is an integer specifying the sequence and `atom` is the atom name e.g `(3,'HA')`. Note that these should match the corresponding sequence and atom in the PDB file.

```
# Load the PCS data
rawData = dataparse.read_pcs('../data_files/calbindin_Er_HN_PCS.npc')
```

To associate the experimental PCS value with atoms of the PDB structure, the method *paramagpy.protein.CustomStructure.parse()* is called on `rawData`. The returned array `parsedData` has a row for each atom with columns `[mdl,atm,exp,cal,err,idx]`, where `mdl` is the model number from the PDB file, `atm` is an atom object from the BioPython PDB structure, `exp` and `cal` are the experimental and calculated values, `err` is the experimental uncertainty and `idx` is the atom index, used to define ensemble averaging behaviour.

```
# Associate PCS data with atoms of the PDB
parsedData = prot.parse(rawData)
```

An initial $\Delta\chi$-tensor is defined by initialising a *paramagpy.metal.Metal* object. The initial position is known to be near the binding site, which is set to the CA atom of residue 56. Note that the `position` attribute is always in Angstrom units.

```
# Define an initial tensor
mStart = metal.Metal()

# Set the starting position to an atom close to the metal
mStart.position = prot[0]['A'][56]['CA'].position
```

A quick gridsearch is conducted in a sphere of 10 Angstrom with 10 points per radius using the function *paramagpy.fit.svd_gridsearch_fit_metal_from_pcs()*. This requires two lists containing the starting metals `mStart` and parsed experimental dataArray `parsedData`. This function returns lists containing a new fitted metal object, the calculated PCS values from the fitted model.

```
# Calculate an initial tensor from an SVD gridsearch
[mGuess], [data] = fit.svd_gridsearch_fit_metal_from_pcs(
        [mStart],[parsedData], radius=10, points=10)
```

This is then refined using a non-linear regression gradient descent with the function *paramagpy.fit.nlr_fit_metal_from_pcs()*.

```
# Refine the tensor using non-linear regression
[mFit], [data] = fit.nlr_fit_metal_from_pcs([mGuess], [parsedData])
```

The Q-factor is then calculated using the function :py:func`paramagpy.fit.qfactor`.

```
# Calculate the Q-factor
qfac = fit.qfactor(data)
```

The fitted tensor parameters are saved by calling the method *paramagpy.metal.Metal.save()*. Alterntaively they may be displayed using `print(mFit.info())`

```
# Save the fitted tensor to file
mFit.save('calbindin_Er_HN_PCS_tensor.txt')
```

Output: [`calbindin_Er_HN_PCS_tensor.txt`]

```
ax    | 1E-32 m^3 :    -8.688
rh    | 1E-32 m^3 :    -4.192
x     |   1E-10 m :    25.517
y     |   1E-10 m :     8.652
z     |   1E-10 m :     6.358
a     |       deg :   116.011
b     |       deg :   138.058
g     |       deg :    43.492
mueff |        Bm :     0.000
shift |       ppm :     0.000
B0    |         T :    18.790
temp  |         K :   298.150
t1e   |        ps :     0.000
taur  |        ns :     0.000
```

These experimental/calculated PCS values are then plotted in a correlation plot to assess the fit. This is achieved using standard functions of the plotting module matplotlib.

```
#### Plot the correlation ####
from matplotlib import pyplot as plt
fig, ax = plt.subplots(figsize=(5,5))

# Plot the data
ax.plot(data['exp'], data['cal'], marker='o', lw=0, ms=3, c='r',
        label="Q-factor = {:5.4f}".format(qfac))

# Plot a diagonal
```

(continues on next page)

(continued from previous page)

```
l, h = ax.get_xlim()
ax.plot([l,h],[l,h],'-k',zorder=0)
ax.set_xlim(l,h)
ax.set_ylim(l,h)

# Make axis labels and save figure
ax.set_xlabel("Experiment")
ax.set_ylabel("Calculated")
ax.legend()
fig.savefig("pcs_fit.png")
```

Output: [pcs_fit.png]

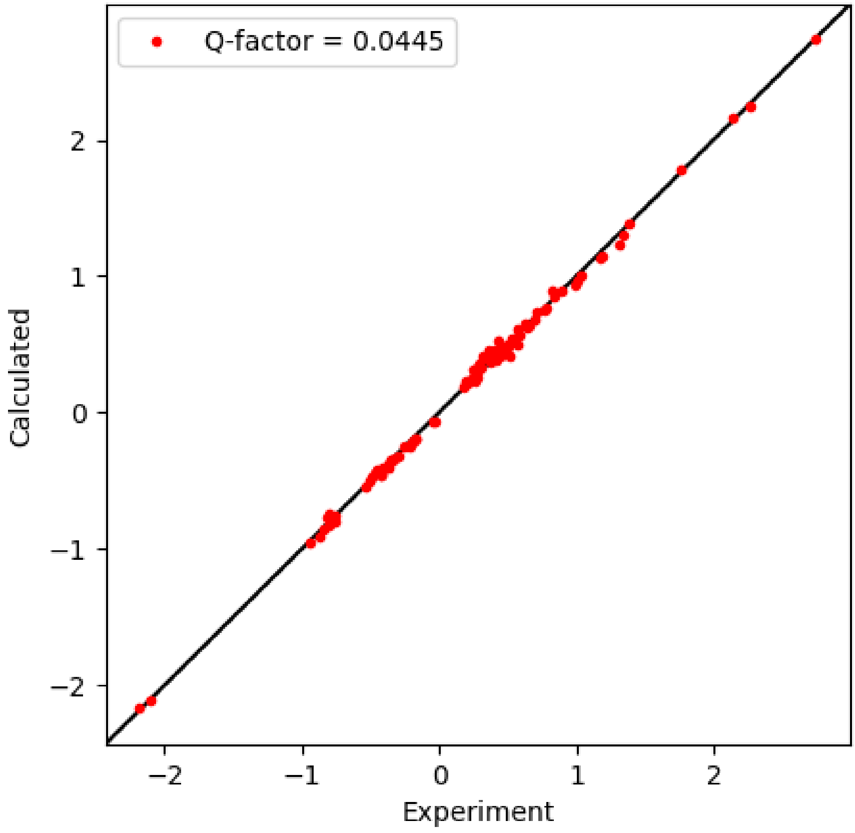

Plot PCS isosurface (PyMol view)

This example shows how to plot the PCS isosurface of a fitted $\Delta\chi$-tensor for data from the example *Fit Tensor to PCS Data*. The isosurface can be viewed in PyMol.

Downloads

- Download the data files `4icbH_mut.pdb` and `calbindin_Er_HN_PCS_tensor.txt` from here:
- Download the script pcs_plot_isosurface.py

Explanation

The protein and tensor are loaded as described previously in.

The isosurface files are generated using the function *paramagpy.metal.Metal.isomap()*. The contour level can be chosen by setting the `isoval` argument. A larger `density` value will result in a smoother surface. This function writes two files `isomap.pml` and `isomap.pml.ccp4` which are the PyMol script and PCS grid files respectively.

The isosurface can be displayed by executing `pymol isomap.pml` from a terminal, or by selecting `File>Run` and navigating to the script `isomap.pml`.

Script

[pcs_plot_isosurface.py]

```
from paramagpy import protein, fit, dataparse, metal

# Load the PDB file
prot = protein.load_pdb('../data_files/4icbH_mut.pdb')

# Load the fitted tensor
met = metal.load_tensor('../data_files/calbindin_Er_HN_PCS_tensor.txt')

# Plot the isosurface to be opened in PyMol
met.isomap(prot.id, density=1, isoval=1.0)
```

Output

PyMol view of isosurface

[pcs_plot_isosurface.png]

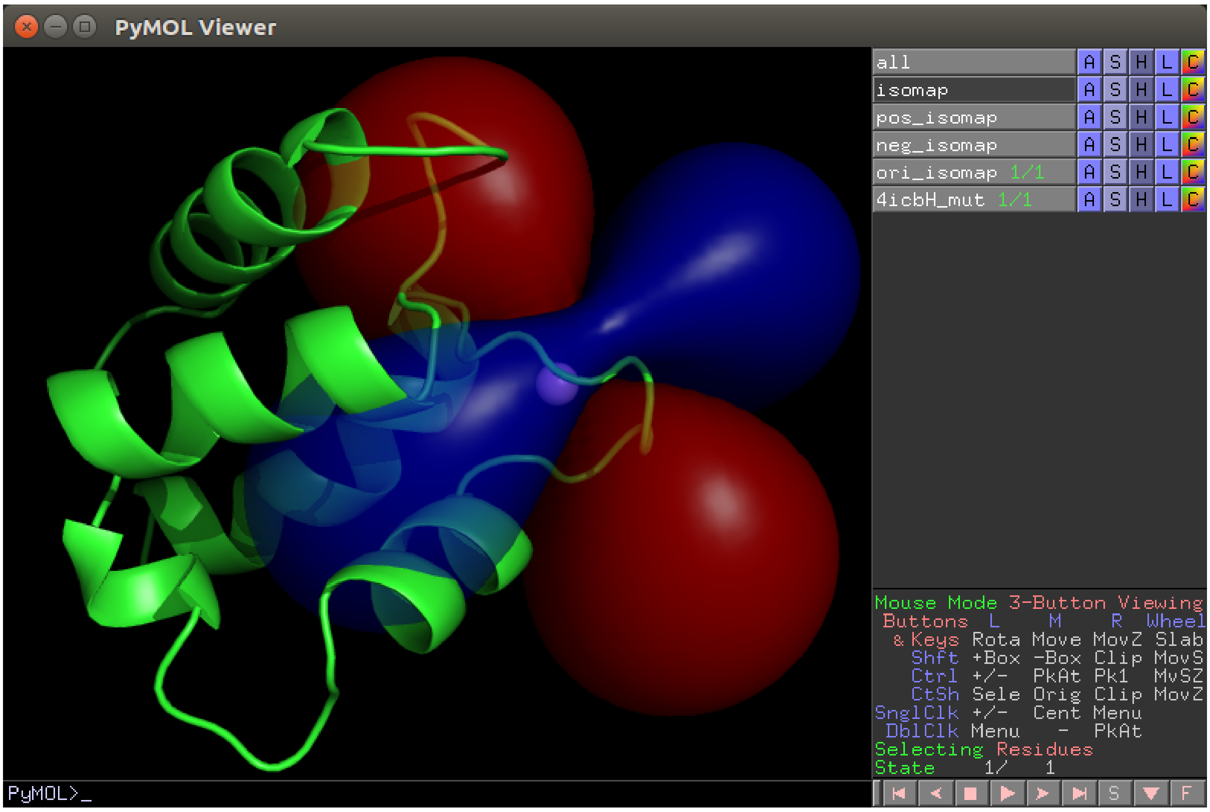

Fit multiple PCS datasets to common position

This example shows how to fit multiple $\Delta\chi$-tensors to their respective datasets with a common position, but varied magnitude and orientation. This may arise if several lanthanides were investigated at the same binding site, and the data may be used simultaneously to fit a common position. Data from several PCS datasets for calbindin D9k were used here, and is a generalisation of the previous example: *Fit Tensor to PCS Data*.

Downloads

- Download the data files `4icbH_mut.pdb`, `calbindin_Tb_HN_PCS.npc`, `calbindin_Er_HN_PCS.npc` and `calbindin_Yb_HN_PCS_tensor.txt` from here:
- Download the script pcs_fit_multiple.py

Explanation

The protein and PCS datasets are loaded and parsed. These are placed into a list `parsedData`, for which each element is a PCS dataset of a given lanthanide.

The two fitting functions:

- *`paramagpy.fit.svd_gridsearch_fit_metal_from_pcs()`*
- *`paramagpy.fit.nlr_fit_metal_from_pcs()`*

can accept a list of metal objects and a list of datasets with arbitrary size. If this list contains more than one element, fitting will be performed to a common position. The starting position is taken only from the first metal of the list.

After fitting, a list of fitted metals is returned. The fitted tensor are then written to files and a correlation plot is made.

Script

[pcs_fit_multiple.py]

```
from paramagpy import protein, fit, dataparse, metal

# Load the PDB file
prot = protein.load_pdb('../data_files/4icbH_mut.pdb')

# Load the PCS data
rawData1 = dataparse.read_pcs('../data_files/calbindin_Tb_HN_PCS.npc')
rawData2 = dataparse.read_pcs('../data_files/calbindin_Er_HN_PCS.npc')
rawData3 = dataparse.read_pcs('../data_files/calbindin_Yb_HN_PCS.npc')

# Associate PCS data with atoms of the PDB
parsedData = []
for rd in [rawData1, rawData2, rawData3]:
        parsedData.append(prot.parse(rd))

# Make a list of starting tensors
mStart = [metal.Metal(), metal.Metal(), metal.Metal()]

# Set the starting position to an atom close to the metal
mStart[0].position = prot[0]['A'][56]['CA'].position

# Calculate initial tensors from an SVD gridsearch
mGuess, datas = fit.svd_gridsearch_fit_metal_from_pcs(
        mStart, parsedData, radius=10, points=10)

# Refine the tensors using non-linear regression
fitParameters = ['x','y','z','ax','rh','a','b','g']
mFit, datas = fit.nlr_fit_metal_from_pcs(mGuess, parsedData, fitParameters)

# Save the fitted tensors to files
for name, metal in zip(['Tb','Er','Yb'], mFit):
        metal.save("tensor_{}.txt".format(name))

#### Plot the correlation ####
from matplotlib import pyplot as plt
fig, ax = plt.subplots(figsize=(5,5))

# Plot the data
for d, name, colour in zip(datas, ['Tb','Er','Yb'],['r','g','b']):
        qfactor = fit.qfactor(d)
        ax.plot(d['exp'], d['cal'], marker='o', lw=0, ms=1, c=colour,
                label="{0:} - {1:5.3f}".format(name, qfactor))

# Plot a diagonal
l, h = ax.get_xlim()
ax.plot([l,h],[l,h],'-k',zorder=0)
ax.set_xlim(l,h)
ax.set_ylim(l,h)

# Axis labels
ax.set_xlabel("Experiment")
ax.set_ylabel("Calculated")
ax.legend()
fig.savefig("pcs_fit_multiple.png")
```

Outputs

Tb fitted tensor

[tensor_Tb.txt]

```
ax    | 1E-32 m^3 :    31.096
rh    | 1E-32 m^3 :    12.328
x     |   1E-10 m :    25.937
y     |   1E-10 m :     9.481
z     |   1E-10 m :     6.597
a     |       deg :   151.053
b     |       deg :   152.849
g     |       deg :    69.821
mueff |        Bm :     0.000
shift |       ppm :     0.000
B0    |         T :    18.790
temp  |         K :   298.150
t1e   |        ps :     0.000
taur  |        ns :     0.000
```

Er fitted tensor

[tensor_Er.txt]

```
ax    | 1E-32 m^3 :    -8.422
rh    | 1E-32 m^3 :    -4.886
x     |   1E-10 m :    25.937
y     |   1E-10 m :     9.481
z     |   1E-10 m :     6.597
a     |       deg :   126.015
b     |       deg :   142.899
g     |       deg :    41.039
mueff |        Bm :     0.000
shift |       ppm :     0.000
B0    |         T :    18.790
temp  |         K :   298.150
t1e   |        ps :     0.000
taur  |        ns :     0.000
```

Yb fitted tensor

[tensor_Yb.txt]

```
ax    | 1E-32 m^3 :    -5.392
rh    | 1E-32 m^3 :    -2.490
x     |   1E-10 m :    25.937
y     |   1E-10 m :     9.481
z     |   1E-10 m :     6.597
a     |       deg :   129.650
b     |       deg :   137.708
g     |       deg :    88.796
mueff |        Bm :     0.000
shift |       ppm :     0.000
B0    |         T :    18.790
temp  |         K :   298.150
t1e   |        ps :     0.000
taur  |        ns :     0.000
```

Correlation Plot

[pcs_fit_multiple.png]

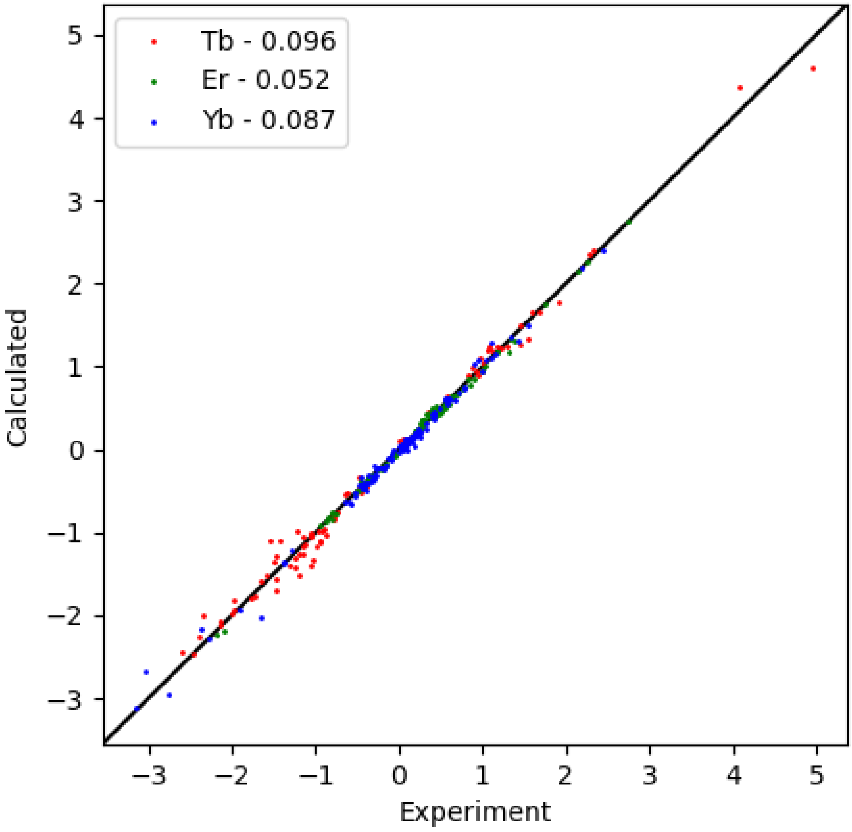

Fit Tensor to PDB with Models

This example shows how to fit a $\Delta\chi$-tensor to experimental PCS data using an NMR structure that contains many models. The tensor can be fit to ensemble averaged PCS values, or to individual models. An ensemble averaged PCS is the mean calculated PCS of all models. No structural averages are ever taken.

Data for calbindin D9k are used as in the previous example *Fit Tensor to PCS Data*.

Downloads

- Download the data files `2bcb.pdb` and `calbindin_Er_HN_PCS.npc` from here:
- Download the script `pcs_fit_models.py`

Script + Explanation

Firstly, the standard preamble and loading of data.

```
from paramagpy import protein, fit, dataparse, metal

# Load data
prot = protein.load_pdb('../data_files/2bcb.pdb')
rawData = dataparse.read_pcs('../data_files/calbindin_Er_HN_PCS.npc')
mStart = metal.Metal()
mStart.position = prot[0]['A'][56]['CA'].position
```

The default method of fitting is to fit the tensor independently to each model of the PDB file. To achieve ensemble averaging behaviour, this requires setting the argument `ensembleAverage` to true within the fitting function. The default ensemble averaging behaviour is to average atoms with the same serial number in the PDB file. To manipulate ensemble averaging, you can specify the `idx` column of the input dataArray for *paramagpy.fit.nlr_fit_metal_from_pcs()*. The `idx` array contains common integers for corresponding atoms to be averaged. After fitting the Q-factor is calculated (with the ensembleAverage argument set to True), and ensemble averaging of the calculated data values is achieved with the function *paramagpy.fit.ensemble_average()*

```
#### Ensemble average fitting ####
parsedData = prot.parse(rawData)
[mGuess], [data] = fit.svd_gridsearch_fit_metal_from_pcs(
        [mStart],[parsedData], radius=10, points=10, ensembleAverage=True)
[mFit], [data] = fit.nlr_fit_metal_from_pcs([mGuess], [parsedData],␣
↪ensembleAverage=True)

qfac = fit.qfactor(data, ensembleAverage=True)
dataEAv = fit.ensemble_average(data)

mFit.save('calbindin_Er_HN_PCS_tensor_ensemble.txt')
```

Fitting a separate tensor to each model of the PDB is the default behaviour of the fitting functions, the average of all fitted tensors is then returned. The model with the minimum Q-factor is then found by looping over the calculated data and sorting them by the calculated Q-factor.

```
#### Single model fitting ####
[mFitMod], [dataMod] = fit.nlr_fit_metal_from_pcs(
        [mGuess], [parsedData])
qs = {}
for mdl in set(dataMod['mdl']):
        qs[mdl] = fit.qfactor(dataMod[dataMod['mdl']==mdl])

minModel, minQfac = sorted(qs.items(), key=lambda x: x[1])[0]
minData = dataMod[dataMod['mdl']==minModel]
```

Finally we plot three sets of data:

- The ensemble average fit calculated for each model (green)
- The ensemble average of the calculated values of the ensemble fit (red)
- The best fitting single model (blue)

Note that to calculate the ensemble average of the calculated values we use the function *paramagpy.fit.ensemble_average()*. This can take any number of arguments, and will average values based on common serial numbers of the list of atoms in the first argument.

```
# #### Plot the correlation ####
from matplotlib import pyplot as plt
fig, ax = plt.subplots(figsize=(5,5))

# Plot all models
ax.plot(data['exp'], data['cal'], marker='o', lw=0, ms=2, c='g',
        alpha=0.5, label="All models: Q = {:5.4f}".format(qfac))

# Plot the ensemble average
ax.plot(dataEAv['exp'], dataEAv['cal'], marker='o', lw=0, ms=2, c='r',
        alpha=0.5, label="Ensemble Average: Q = {:5.4f}".format(qfac))

# Plot the model with minimum Q-factor
ax.plot(minData['exp'], minData['cal'], marker='o', lw=0, ms=2, c='b',
        alpha=0.5, label="Best Model ({0:}): Q = {1:5.4f}".format(
                minModel, minQfac))
```

(continues on next page)

(continued from previous page)

```
# Plot a diagonal
l, h = ax.get_xlim()
ax.plot([l,h],[l,h],'-k',zorder=0)
ax.set_xlim(l,h)
ax.set_ylim(l,h)

# Make axis labels and save figure
ax.set_xlabel("Experiment")
ax.set_ylabel("Calculated")
ax.legend()
fig.savefig("pcs_fit_models.png")
```

Output: [pcs_fit_models.png]

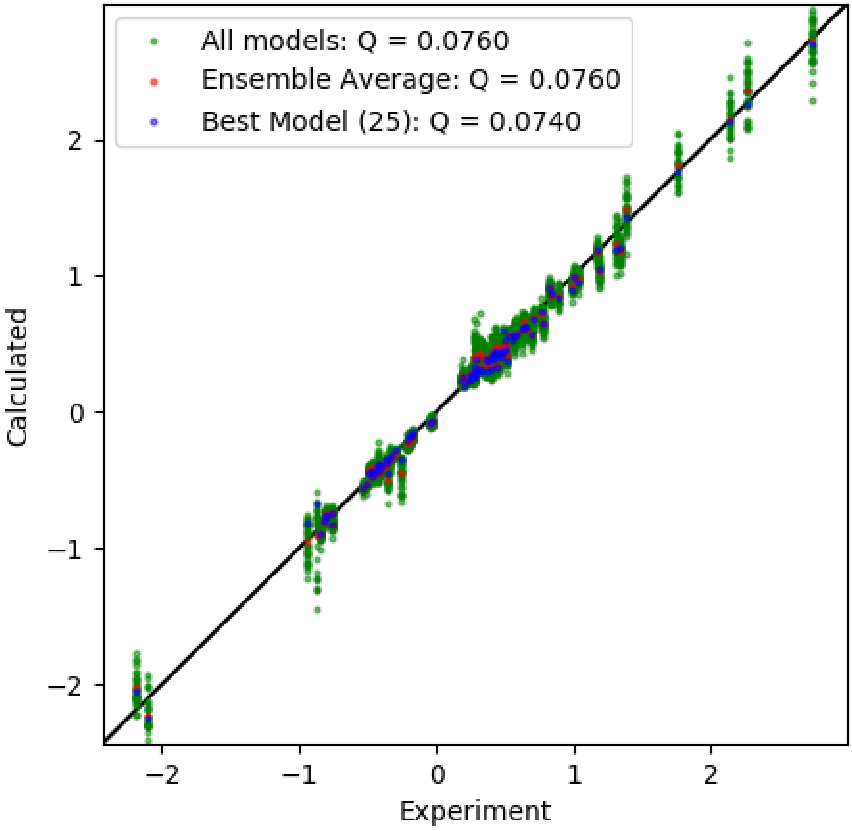

Constrained Fitting

This example shows how to fit a $\Delta\chi$-tensor with constraints applied. The two cases here constrain position to fit a tensor to a known metal ion position form an X-ray structure, and fit an axially symmetric tensor with only 6 of the usual 8 parameters.

Downloads

- Download the data files `4icbH_mut.pdb` and `calbindin_Er_HN_PCS.npc` from here:
- Download the script `pcs_fit_constrained.py`

Script + Explanation

The necessary modules are imported and data is loaded

```
from paramagpy import protein, fit, dataparse, metal

# Load data
prot = protein.load_pdb('../data_files/4icbH_mut.pdb')
rawData = dataparse.read_pcs('../data_files/calbindin_Er_HN_PCS.npc')
parsedData = prot.parse(rawData)
mStart = metal.Metal()
```

The calcium ion from the X-ray structure is contained in a heteroatom of the PDB file. We set the starting position of the tensor to this position.

```
# Set the starting position to Calcium ion heteroatom in PDB
mStart.position = prot[0]['A'][('H_ CA', 77, ' ')]['CA'].position
```

To fit the the anisotropy and orientation without position, the linear PCS equation can be solved analytically by the SVD gridsearch method but using only one point with a radius of zero. The Q-factor is then calculated and the tensor is saved.

```
# Calculate tensor by SVD
[mFit], [data] = fit.svd_gridsearch_fit_metal_from_pcs(
        [mStart],[parsedData], radius=0, points=1)

qfac = fit.qfactor(data)

mFit.save('calbindin_Er_HN_PCS_tensor_position_constrained.txt')
```

Output: [`pcs_fit_constrained.png`]

```
ax    | 1E-32 m^3 :    -8.152
rh    | 1E-32 m^3 :    -4.911
x     |   1E-10 m :    25.786
y     |   1E-10 m :     9.515
z     |   1E-10 m :     6.558
a     |       deg :   125.841
b     |       deg :   142.287
g     |       deg :    41.758
mueff |        Bm :     0.000
shift |       ppm :     0.000
B0    |         T :    18.790
temp  |         K :   298.150
t1e   |        ps :     0.000
taur  |        ns :     0.000
```

To fit an axially symmetric tensor, we can used the Non-linear regression method and specify exactly which parameters we want to fit. This will be the axiality `ax`, two Euler angles `b` and `g` and the position coordinates. Note that in the output, the rhombic `rh` and alpha `a` parameters are redundant.

```
# Calculate axially symmetric tensor by NRL
[mFitAx], [dataAx] = fit.nlr_fit_metal_from_pcs(
        [mStart], [parsedData], params=('ax','b','g','x','y','z'))

qfacAx = fit.qfactor(dataAx)

mFitAx.save('calbindin_Er_HN_PCS_tensor_axially_symmetric.txt')
```

Output: [pcs_fit_constrained.png]

```
ax    | 1E-32 m^3 :     9.510
rh    | 1E-32 m^3 :     0.000
x     |   1E-10 m :    24.948
y     |   1E-10 m :     8.992
z     |   1E-10 m :     3.205
a     |       deg :     0.000
b     |       deg :   134.697
g     |       deg :   180.000
mueff |        Bm :     0.000
shift |       ppm :     0.000
B0    |         T :    18.790
temp  |         K :   298.150
t1e   |        ps :     0.000
taur  |        ns :     0.000
```

Finally we plot the data.

```
#### Plot the correlation ####
from matplotlib import pyplot as plt
fig, ax = plt.subplots(figsize=(5,5))

# Plot the data
ax.plot(data['exp'], data['cal'], marker='o', lw=0, ms=2, c='r',
        label="Position constrained: Q = {:5.4f}".format(qfac))

ax.plot(dataAx['exp'], dataAx['cal'], marker='o', lw=0, ms=2, c='b',
        label="Axially symmetric: Q = {:5.4f}".format(qfacAx))

# Plot a diagonal
l, h = ax.get_xlim()
ax.plot([l,h],[l,h],'-k',zorder=0)
ax.set_xlim(l,h)
ax.set_ylim(l,h)

# Make axis labels and save figure
ax.set_xlabel("Experiment")
ax.set_ylabel("Calculated")
ax.legend()
fig.savefig("pcs_fit_constrained.png")
```

Output: [pcs_fit_constrained.png]

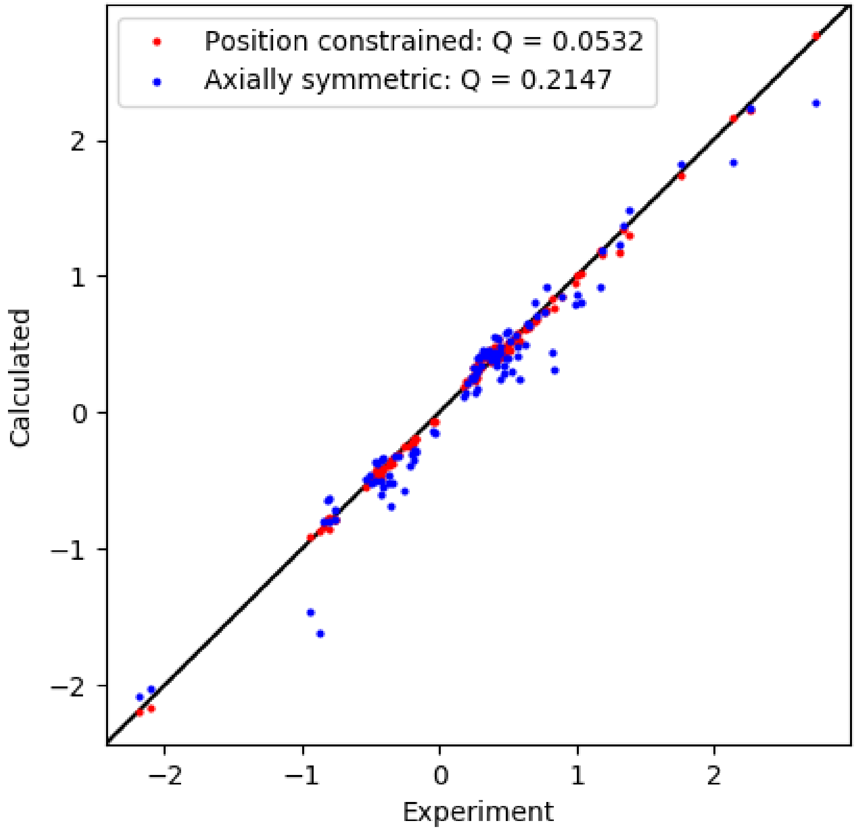

Fit a tensor to PCS data with uncertainties

This example shows how to conduct a weighted fit of a $\Delta\chi$-tensor to experimental PCS data with experimental errors.

Downloads

- Download the data files `4icbH_mut.pdb` and `calbindin_Er_HN_PCS_errors.npc` from here:
- Download the script `pcs_fit_error.py`

Script + Explanation

This script follows very closely the script *Fit Tensor to PCS Data*. The only difference being that errors are included in the fourth column of the .npc file and errorbars are included in the plotting routine.

```
from paramagpy import protein, fit, dataparse, metal

# Load the PDB file
prot = protein.load_pdb('../data_files/4icbH_mut.pdb')

# Load the PCS data
rawData = dataparse.read_pcs('../data_files/calbindin_Er_HN_PCS_errors.npc')

# Associate PCS data with atoms of the PDB
```

(continues on next page)

(continued from previous page)

```
parsedData = prot.parse(rawData)

# Define an initial tensor
mStart = metal.Metal()

# Set the starting position to an atom close to the metal
mStart.position = prot[0]['A'][56]['CA'].position

# Calculate an initial tensor from an SVD gridsearch
[mGuess], [data] = fit.svd_gridsearch_fit_metal_from_pcs(
        [mStart],[parsedData], radius=10, points=10)

# Refine the tensor using non-linear regression
[mFit], [data] = fit.nlr_fit_metal_from_pcs([mGuess], [parsedData])

qfac = fit.qfactor(data)

# Save the fitted tensor to file
mFit.save('calbindin_Er_HN_PCS_tensor_errors.txt')

#### Plot the correlation ####
from matplotlib import pyplot as plt
fig, ax = plt.subplots(figsize=(5,5))

# Plot the data
ax.errorbar(data['exp'], data['cal'], xerr=data['err'], fmt='o', c='r', ms=2,
        ecolor='k', capsize=3, label="Q-factor = {:5.4f}".format(qfac))

# Plot a diagonal
l, h = ax.get_xlim()
ax.plot([l,h],[l,h],'grey',zorder=0)
ax.set_xlim(l,h)
ax.set_ylim(l,h)

# Make axis labels and save figure
ax.set_xlabel("Experiment")
ax.set_ylabel("Calculated")
ax.legend()
fig.savefig("pcs_fit_error.png")
```

The fitted tensor:

Output: [calbindin_Er_HN_PCS_tensor_errors.txt]

```
ax    | 1E-32 m^3 :    -8.012
rh    | 1E-32 m^3 :    -4.125
x     |   1E-10 m :    24.892
y     |   1E-10 m :     8.456
z     |   1E-10 m :     6.287
a     |       deg :   112.440
b     |       deg :   135.924
g     |       deg :    46.210
mueff |        Bm :     0.000
shift |       ppm :     0.000
B0    |         T :    18.790
temp  |         K :   298.150
t1e   |        ps :     0.000
taur  |        ns :     0.000
```

And correlation plot:

Output: [pcs_fit_error.png]

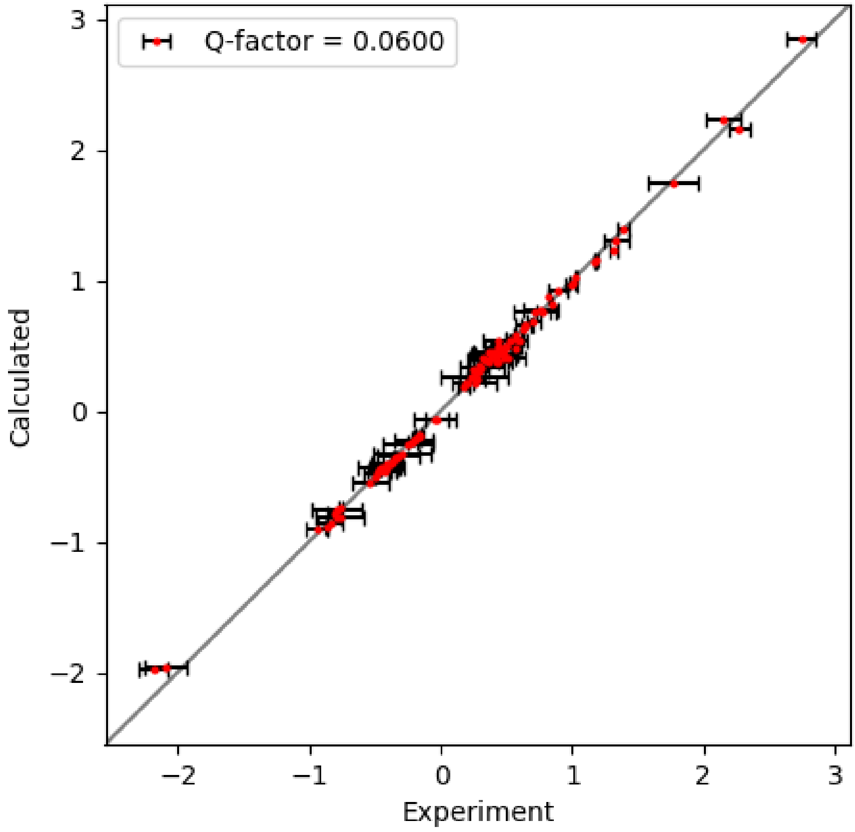

Propagate Uncertainty to Fitted Tensor Parameters

This example shows the various error analysis functions available in paramagpy for estimating the unceratinty in fitted parameters for a paramagnetic center.

Downloads

- Download the data files `2bcb.pdb` and `calbindin_Er_HN_PCS_errors.npc` from here:
- Download the script `pcs_fit_uncertainty.py`

Script + Explanation

This start of this script follows the script *Fit Tensor to PCS Data* to fit the tensor.

```
from paramagpy import protein, fit, dataparse, metal
import numpy as np

# Load the PDB file
prot = protein.load_pdb('../data_files/2bcb.pdb')

# Load the PCS data
rawData = dataparse.read_pcs('../data_files/calbindin_Er_HN_PCS_errors.npc')

# Associate PCS data with atoms of the PDB
```

(continues on next page)

(continued from previous page)

```
parsedData = prot.parse(rawData)

# Define an initial tensor
mStart = metal.Metal()

# Set the starting position to an atom close to the metal
mStart.position = prot[0]['A'][56]['CA'].position

# Calculate an initial tensor from an SVD gridsearch
[mGuess], [data] = fit.svd_gridsearch_fit_metal_from_pcs(
        [mStart],[parsedData], radius=10, points=10)

# Refine the tensor using non-linear regression
[mFit], [data] = fit.nlr_fit_metal_from_pcs([mGuess], [parsedData])
```

Uncertainty from structure models

The PDB file contains models that capture uncertainty in the structure of the protein. This can be propagated to estimate uncertainty in the fitted tensor parameters using the fnction `paramagpy.fit.fit_error_model()`. This fits a separate tensor to each model and returns all fitted tensors as well as the standard deviation in the fitted parameters.

```
# Estimate uncertainty sourcing noise from the models of the PDB
[mod_all], [mod_std] = fit.fit_error_models(fit.nlr_fit_metal_from_pcs,
        initMetals=[mFit], dataArrays=[parsedData])

mod_std.save('error_tensor_models.txt')
```

The standard deviation in the fitted tensor parameters is found in the variable `mod_std`. This variation in tensor principle axes can be viewed by a Sanson-Flamsteed plot.

Output: [`error_tensor_models.txt`]

```
ax     | 1E-32 m^3 :     0.556
rh     | 1E-32 m^3 :     0.525
x      |   1E-10 m :     0.756
y      |   1E-10 m :     0.695
z      |   1E-10 m :     0.957
a      |       deg :     7.466
b      |       deg :     9.948
g      |       deg :    19.294
mueff  |        Bm :     0.000
shift  |       ppm :     0.000
B0     |         T :     0.000
temp   |         K :     0.000
t1e    |        ps :     0.000
taur   |        ns :     0.000
```

Output: [`models.png`]

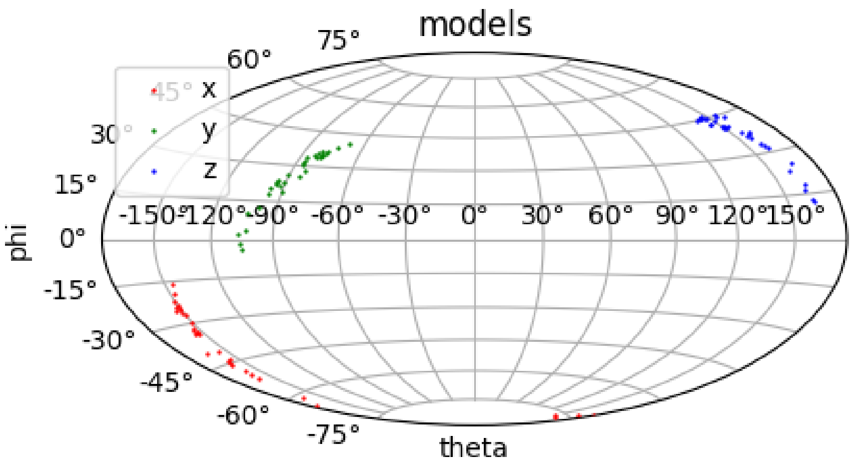

Uncertainty from experimental uncertainties

Experimental uncertainties can be measured. This may arise due to spectral noise in peak heights for PREs, or spectral noise as uncertainties in chemical shifts for PCSs, as is the case here. The function *paramagpy.fit.fit_error_monte_carlo()* will repeat the fit for many iterations, each time adding random noise from a uniform distribution scaled by the experimental errors present in the `err` column of the dataArray `parsedData`.

```
# Estimate uncertainty sourcing noise from experimental uncertainties
[mc_all], [mc_std] = fit.fit_error_monte_carlo(fit.nlr_fit_metal_from_pcs,
        50, initMetals=[mFit], dataArrays=[parsedData])

mod_std.save('error_tensor_monte_carlo.txt')
```

Output: [`error_tensor_monte_carlo.txt`]

```
ax    | 1E-32 m^3 :     0.556
rh    | 1E-32 m^3 :     0.525
x     |   1E-10 m :     0.756
y     |   1E-10 m :     0.695
z     |   1E-10 m :     0.957
a     |       deg :     7.466
b     |       deg :     9.948
g     |       deg :    19.294
mueff |        Bm :     0.000
shift |       ppm :     0.000
B0    |         T :     0.000
temp  |         K :     0.000
t1e   |        ps :     0.000
taur  |        ns :     0.000
```

Output: [`monte_carlo.png`]

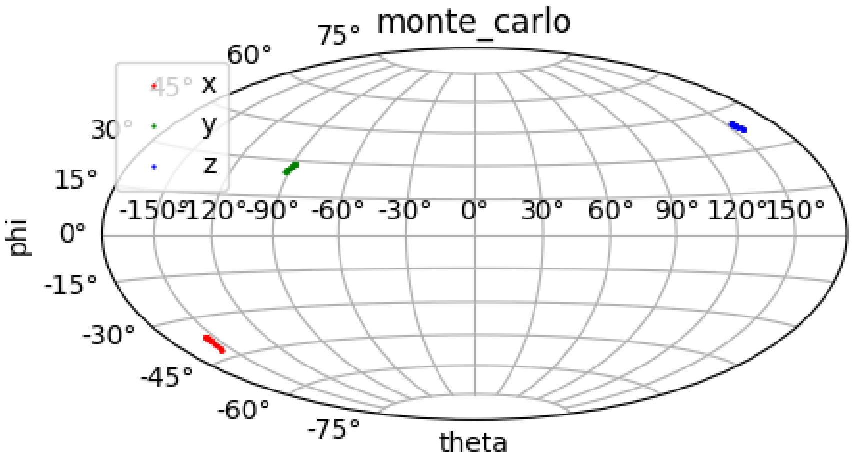

Uncertainty from sample fraction

A final, but generally not recommended method is to source noise from taking a random fraction of the data and conducting the fit for many iterations to then view the deviation in fitted parameters. This method is often called bootstrapping and is desirable if the experimental uncertainties are unknown and the PDB file does not contain models that capture structural uncertainty. The function *paramagpy.fit.fit_error_bootstrap()* will repeat the fit for many iterations, each time sampling the desired amount of the experimental data randomly.

```
# Estimate uncertainty sourcing noise from sample fractions
[bs_all], [bs_std] = fit.fit_error_bootstrap(fit.nlr_fit_metal_from_pcs,
        50, 0.8, initMetals=[mFit], dataArrays=[parsedData])

mod_std.save('error_tensor_bootstrap.txt')
```

Output: [error_tensor_bootstrap.txt]

```
ax     | 1E-32 m^3 :     0.556
rh     | 1E-32 m^3 :     0.525
x      |   1E-10 m :     0.756
y      |   1E-10 m :     0.695
z      |   1E-10 m :     0.957
a      |       deg :     7.466
b      |       deg :     9.948
g      |       deg :    19.294
mueff  |        Bm :     0.000
shift  |       ppm :     0.000
B0     |         T :     0.000
temp   |         K :     0.000
t1e    |        ps :     0.000
taur   |        ns :     0.000
```

Output: [bootstrap.png]

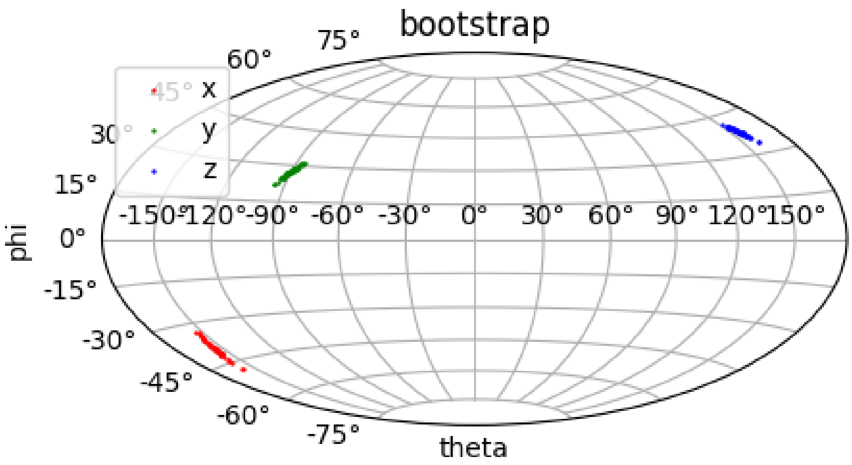

This piece of code is used to generate the Sanson-Flamsteed projection plots

```
#### Plot Sanson-Flamsteed ####
from matplotlib import pyplot as plt

def transform(vector):
        x, y, z = vector
        theta = np.arctan2(y, x)
        phi = -np.arccos(z) + np.pi/2.
        return theta, phi

for name, mset in [('models',mod_all), ('monte_carlo',mc_all), ('bootstrap',bs_
↪all)]:
        spcoords = []
        for m in mset:
                x, y, z = m.rotationMatrix.T
                spcoords.append(tuple(map(transform, [x,y,z])))
        points = zip(*spcoords)
        fig = plt.figure(figsize=(5, 3), dpi=100)
        ax = fig.add_subplot(111, projection='hammer')
        ax.set_xlabel("theta")
        ax.set_ylabel("phi")
        ax.set_title(name)
        ax.grid()
        for data, col, label in zip(points, ['r','g','b'], ['x','y','z']):
                theta, phi = zip(*data)
                ax.scatter(theta, phi, s=0.4, c=col, label=label, zorder=10)
        ax.legend()
        fig.savefig("{}.png".format(name))
```

Fit to PCS data with offset, RACS and RADS corrections

5.2.2 RDC data

Fit Tensor to RDC Data

This example shows how to fit a $\Delta\chi$-tensor or equivalently, and alignment tensor to experimental RDC data. These data are taken from a Tb3+ tagged ubiquitin mutant:

Benjamin J. G. Pearce, Shereen Jabar, Choy-Theng Loh, Monika Szabo, Bim Graham, Gottfried Otting (2017) Structure restraints from heteronuclear pseudocontact shifts generated by lanthanide tags at two different sites *J.*

Biomol. NMR 68:19-32

Downloads

- Download the data files `2kox.pdb`, `ubiquitin_a28c_c1_Tb_HN.rdc` and `ubiquitin_s57c_c1_Tb_HN.rdc` from here:
- Download the script `rdc_fit.py`

Script + Explanation

Firstly, the necessary modules are imported from paramagpy. And the two RDC datasets are loaded. Because this PDB contains over 600 models, loading may take a few seconds

```
from paramagpy import protein, fit, dataparse, metal

# Load the PDB file
prot = protein.load_pdb('../data_files/2kox.pdb')

# Load the RDC data
rawData1 = dataparse.read_rdc('../data_files/ubiquitin_a28c_c1_Tb_HN.rdc')
rawData2 = dataparse.read_rdc('../data_files/ubiquitin_s57c_c1_Tb_HN.rdc')

# Associate RDC data with atoms of the PDB
parsedData1 = prot.parse(rawData1)
parsedData2 = prot.parse(rawData2)
```

Two starting metals are initialised. It is important here to set the magnetic field strength and temperature.

```
# Define an initial tensor
mStart1 = metal.Metal(B0=18.8, temperature=308.0)
mStart2 = metal.Metal(B0=18.8, temperature=308.0)
```

The alignment tensor is solved using the function *paramagpy.fit.svd_fit_metal_from_rdc()* which return a tuple of `(metal, calculated)`, where metal is the fitted metal, calculated is the calculated RDC values. The tensors are then saved. Note that we set the argument `ensembleAverage` to `True`. This is important because the PDB structure represents and MD simulation. If set to False, a much smaller tensor would be fit.

```
# Calculate the tensor using SVD
[sol1], [data1] = fit.svd_fit_metal_from_rdc([mStart1], [parsedData1],␣
↪ensembleAverage=True)
[sol2], [data2] = fit.svd_fit_metal_from_rdc([mStart2], [parsedData2],␣
↪ensembleAverage=True)

# Save the fitted tensor to file
sol1.save('ubiquitin_a28c_c1_Tb_tensor.txt')
sol2.save('ubiquitin_s57c_c1_Tb_tensor.txt')
```

Output: [`ubiquitin_a28c_c1_Tb_tensor.txt`]

```
ax    | 1E-32 m^3 :    -4.776
rh    | 1E-32 m^3 :    -1.397
x     |   1E-10 m :     0.000
y     |   1E-10 m :     0.000
z     |   1E-10 m :     0.000
a     |       deg :    16.022
b     |       deg :    52.299
g     |       deg :    83.616
```

(continues on next page)

(continued from previous page)

```
mueff  |         Bm :      0.000
shift  |        ppm :      0.000
B0     |          T :     18.800
temp   |          K :    308.000
t1e    |         ps :      0.000
taur   |         ns :      0.000
```

Output: [ubiquitin_s57c_c1_Tb_tensor.txt]

```
ax     | 1E-32 m^3 :     -5.930
rh     | 1E-32 m^3 :     -1.899
x      |   1E-10 m :      0.000
y      |   1E-10 m :      0.000
z      |   1E-10 m :      0.000
a      |       deg :      9.976
b      |       deg :     99.463
g      |       deg :     37.410
mueff  |        Bm :      0.000
shift  |       ppm :      0.000
B0     |         T :     18.800
temp   |         K :    308.000
t1e    |        ps :      0.000
taur   |        ns :      0.000
```

The experimental/calculated correlations are then plotted. The tensor is by default fitted to the ensemble averaged calculated values. Backcalculation of all models is shown here, as well as the ensemble average.

```
#### Plot the correlation ####
from matplotlib import pyplot as plt
fig = plt.figure(figsize=(5,10))
ax1 = fig.add_subplot(211)
ax2 = fig.add_subplot(212)
ax1.set_title('A28C-C1-Tb')
ax2.set_title('S57C-C1-Tb')

for sol, ax, data in zip([sol1,sol2], [ax1,ax2], [data1,data2]):

        # Calculate ensemble averages
        dataEAv = fit.ensemble_average(data)

        # Calculate the Q-factor
        qfac = fit.qfactor(data, ensembleAverage=True)

        # Plot all models
        ax.plot(data['exp'], data['cal'], marker='o', lw=0, ms=2, c='b',
                alpha=0.5, label="All models: Q = {:5.4f}".format(qfac))

        # Plot the ensemble average
        ax.plot(dataEAv['exp'], dataEAv['cal'], marker='o', lw=0, ms=2, c='r',
                label="Ensemble Average: Q = {:5.4f}".format(qfac))

        # Plot a diagonal
        l, h = ax.get_xlim()
        ax.plot([l,h],[l,h],'-k',zorder=0)
        ax.set_xlim(l,h)
        ax.set_ylim(l,h)

        # Make axis labels and save figure
        ax.set_xlabel("Experiment")
        ax.set_ylabel("Calculated")
        ax.legend()
```

(continues on next page)

(continued from previous page)

```
fig.tight_layout()
fig.savefig("rdc_fit.png")
```

Output: [rdc_fit.png]

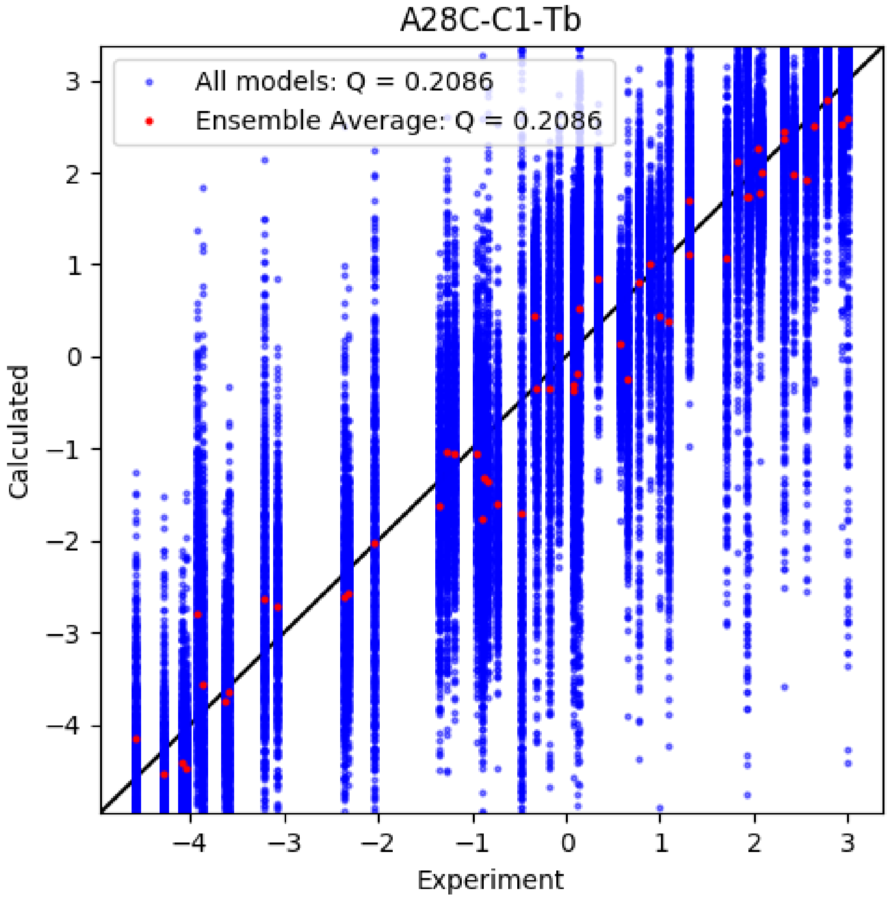
A28C-C1-Tb
All models: Q = 0.2086
Ensemble Average: Q = 0.2086
Calculated
Experiment

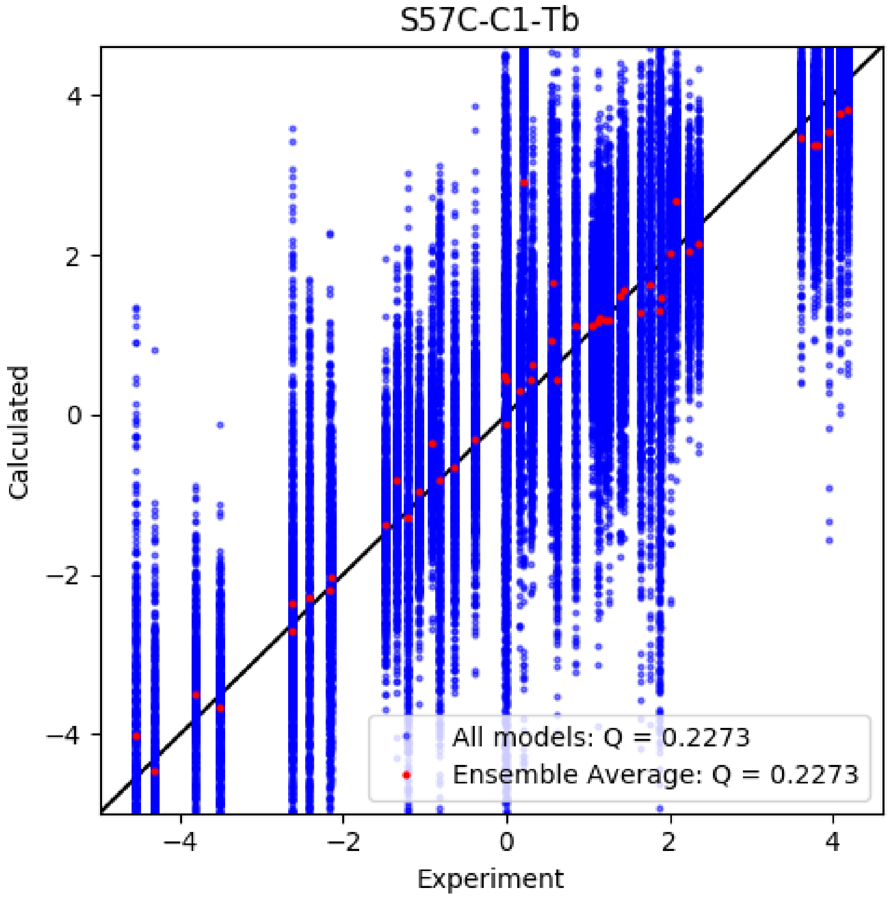
S57C-C1-Tb
All models: Q = 0.2273
Ensemble Average: Q = 0.2273
Calculated
Experiment

Calculate RDC from a known Tensor

This example shows how to calculate theoretical RDC values from a known $\Delta\chi$-tensor which has been fitted from PCS data. Paramagpy allows seamless calculation of one PCS/PRE/RDC/CCR effect from a tensor fitted from another effect.

Downloads

- Download the data files `4icbH_mut.pdb` and `calbindin_Er_HN_PCS_tensor.txt` from here:
- Download the script `rdc_calculate.py`

Script + Explanation

First the relevant modules are loaded, the protein is loaded and the metal is loaded from file. The magnetic field strength and temperature are also set.

```
from paramagpy import protein, metal

# Load the PDB file
prot = protein.load_pdb('../data_files/4icbH_mut.pdb')

# Load the fitted tensor
met = metal.load_tensor('../data_files/calbindin_Er_HN_PCS_tensor.txt')
met.B0 = 18.8
```

A loop is made over the atoms of the protein. The amide H and N atoms are selected and then the RDC value is calculated. Finally the formated data is appended to list `forFile`.

```
forFile = []
for atom in prot.get_atoms():
        if atom.name == 'H':
                residue = atom.parent
                seq = residue.id[1]
                if 'N' in residue:
                        H = atom
                        N = residue['N']
                        rdc = met.atom_rdc(H, N)
                        line = "{0:2d} {1:^3s} {2:2d} {3:^3s} {4:6.3f} 0.0\n".
↪format(
                                seq, H.name, seq, N.name, rdc)
                        forFile.append(line)
```

The formatted data is written to file:

```
with open("calbindin_Er_RDC_calc.rdc", 'w') as f:
        f.writelines(forFile)
```

Output: [`calbindin_Er_RDC_calc.rdc`]

```
 0  H    0  N  -1.724 0.0
 1  H    1  N  -6.196 0.0
 2  H    2  N  -4.993 0.0
 4  H    4  N  -0.922 0.0
 5  H    5  N   1.783 0.0
 6  H    6  N   0.280 0.0
 7  H    7  N  -1.906 0.0
 8  H    8  N   1.056 0.0
 9  H    9  N   0.713 0.0
```

(continues on next page)

(continued from previous page)

```
10   H  10   N    0.213 0.0
11   H  11   N   -0.881 0.0
12   H  12   N    2.712 0.0
13   H  13   N    0.614 0.0
14   H  14   N   -2.346 0.0
15   H  15   N    1.659 0.0
16   H  16   N    0.648 0.0
17   H  17   N    0.383 0.0
18   H  18   N    0.420 0.0
19   H  19   N   -7.863 0.0
21   H  21   N    0.973 0.0
22   H  22   N    1.026 0.0
23   H  23   N   -0.613 0.0
24   H  24   N   -5.847 0.0
25   H  25   N    1.761 0.0
26   H  26   N    6.470 0.0
27   H  27   N    5.541 0.0
28   H  28   N   -0.334 0.0
29   H  29   N    3.624 0.0
30   H  30   N    6.673 0.0
31   H  31   N    3.952 0.0
32   H  32   N    1.658 0.0
33   H  33   N    5.449 0.0
34   H  34   N    7.370 0.0
35   H  35   N    1.033 0.0
36   H  36   N    1.136 0.0
38   H  38   N   -7.378 0.0
39   H  39   N   -6.979 0.0
40   H  40   N   -4.810 0.0
41   H  41   N   -3.187 0.0
42   H  42   N    2.415 0.0
43   H  43   N    1.710 0.0
44   H  44   N   -5.977 0.0
45   H  45   N   -5.467 0.0
46   H  46   N    3.243 0.0
47   H  47   N    3.937 0.0
48   H  48   N    7.047 0.0
49   H  49   N    4.577 0.0
50   H  50   N    3.718 0.0
51   H  51   N    4.519 0.0
52   H  52   N    6.077 0.0
53   H  53   N    2.940 0.0
54   H  54   N    2.541 0.0
55   H  55   N   -7.493 0.0
56   H  56   N   -7.159 0.0
57   H  57   N    4.948 0.0
58   H  58   N   -1.078 0.0
59   H  59   N   -0.759 0.0
60   H  60   N    0.161 0.0
61   H  61   N   -1.132 0.0
62   H  62   N   -5.719 0.0
63   H  63   N    4.025 0.0
64   H  64   N    5.929 0.0
65   H  65   N    2.363 0.0
66   H  66   N    2.477 0.0
67   H  67   N    8.265 0.0
68   H  68   N    5.078 0.0
69   H  69   N    3.724 0.0
70   H  70   N    7.743 0.0
71   H  71   N    2.188 0.0
72   H  72   N    4.911 0.0
```

(continues on next page)

(continued from previous page)

```
73   H   73   N    7.514 0.0
74   H   74   N   -0.001 0.0
75   H   75   N    1.119 0.0
```

5.2.3 PRE data

Fit Tensor to PRE Data

This example demonstrates fitting of the rotational correlation time τ_r to 1H PRE data of calbindin D9k. You can fit any parameters of the χ-tensor you desire, such as position or magnitude as well.

Downloads

- Download the data files `4icbH_mut.pdb`, `calbindin_Er_H_R2_600.npc` and `calbindin_Tb_H_R2_800.npc` from here:
- Download the script `pre_fit_proton.py`

Script + Explanation

Firstly, the necessary modules are imported from paramagpy.

```
from paramagpy import protein, fit, dataparse, metal
```

The protein is then loaded from a PDB file.

```
# Load the PDB file
prot = protein.load_pdb('../data_files/4icbH_mut.pdb')
```

The PRE data is loaded. Note that the Er data was recorded at 600 MHz and the Tb data was recorded at 800 MHz.

```
rawData_er = dataparse.read_pre('../data_files/calbindin_Er_H_R2_600.pre')
rawData_tb = dataparse.read_pre('../data_files/calbindin_Tb_H_R2_800.pre')
```

The $\Delta\chi$-tensors that were fitted from PCS data are loaded from file and the relevant B_0 magnetic field strengths are set.

```
mStart_er = metal.load_tensor('../data_files/calbindin_Er_HN_PCS_tensor.txt')
mStart_tb = metal.load_tensor('../data_files/calbindin_Tb_HN_PCS_tensor.txt')
mStart_er.B0 = 14.1
mStart_tb.B0 = 18.8
```

Fitting of the rotational correlation time is done with the function *paramagpy.fit.nlr_fit_metal_from_pre()*. To fit position or χ-tensor magnitude, you can change the `params` argument.

```
(m_er,), (cal_er,) = fit.nlr_fit_metal_from_pre(
        [mStart_er], [data_er], params=['taur'], rtypes=['r2'])
(m_tb,), (cal_tb,) = fit.nlr_fit_metal_from_pre(
        [mStart_tb], [data_tb], params=['taur'], rtypes=['r2'])
```

The fitted tensors are saved to file. Note that the Er dataset gives a reasonable τ_r of around 4 ns which is close to the literature value of 4.25 ns. However, the Tb dataset gives an unreasonably large value of 18 ns. This is due to magnetisation attenuation due to 1H-1H RDCs present during the relaxation evolution time as discussed in literature giving rise to artificially large measured PREs for lanthanides with highly anisotropic $\Delta\chi$-tensors. This is also reflected in the correlation plot below.

```
m_er.save('calbindin_Er_H_R2_600_tensor.txt')
m_tb.save('calbindin_Tb_H_R2_800_tensor.txt')
```

Output: [calbindin_Er_H_R2_600_tensor.txt]

```
ax    | 1E-32 m^3 :    -8.152
rh    | 1E-32 m^3 :    -4.911
x     |   1E-10 m :    25.786
y     |   1E-10 m :     9.515
z     |   1E-10 m :     6.558
a     |       deg :   125.841
b     |       deg :   142.287
g     |       deg :    41.758
mueff |        Bm :     9.581
shift |       ppm :     0.000
B0    |         T :    14.100
temp  |         K :   298.150
t1e   |        ps :     0.189
taur  |        ns :     3.923
```

Output: [calbindin_Tb_H_R2_800_tensor.txt]

```
ax    | 1E-32 m^3 :    30.375
rh    | 1E-32 m^3 :    12.339
x     |   1E-10 m :    25.786
y     |   1E-10 m :     9.515
z     |   1E-10 m :     6.558
a     |       deg :   150.957
b     |       deg :   152.671
g     |       deg :    70.311
mueff |        Bm :     9.721
shift |       ppm :     0.000
B0    |         T :    18.800
temp  |         K :   298.150
t1e   |        ps :     0.251
taur  |        ns :    18.917
```

And the results are plotted.

```
from matplotlib import pyplot as plt
fig, ax = plt.subplots(figsize=(5,5))

# Plot the data
ax.plot(cal_er['exp'], cal_er['cal'], marker='o', lw=0, ms=3, c='r',
        label="Er: taur = {:3.1f} ns".format(1E9*m_er.taur))
ax.plot(cal_tb['exp'], cal_tb['cal'], marker='o', lw=0, ms=3, c='g',
        label="Tb: taur = {:3.1f} ns".format(1E9*m_tb.taur))

# Plot a diagonal
l, h = ax.get_ylim()
ax.plot([l,h],[l,h],'-k',zorder=0)
ax.set_xlim(l,h)
ax.set_ylim(l,h)

# Make axis labels and save figure
ax.set_xlabel("Experiment")
ax.set_ylabel("Calculated")
ax.legend()
fig.savefig("pre_fit_proton.png")
```

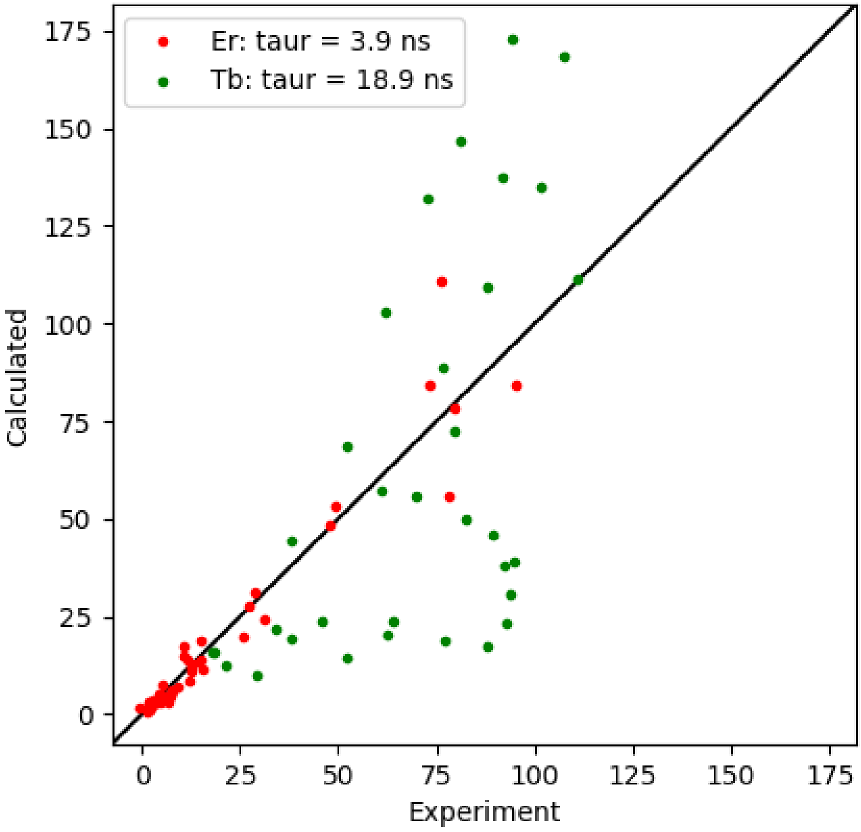

Calculate 15N PREs with cross-correlation effects

This example shows how to conduct a weighted fit of a $\Delta\chi$-tensor to experimental PCS data with experimental errors.

Downloads

- Download the data files `4icbH_mut.pdb`, `calbindin_Tb_N_R1_600.pre` and `calbindin_Tb_HN_PCS_tensor.txt` from here:
- Download the script `pre_calc_nitrogen.py`

Script + Explanation

First the relevant modules are loaded, the protein and data are read and the data is parsed by the protein.

```
from paramagpy import protein, metal, dataparse

# Load the PDB file
prot = protein.load_pdb('../data_files/4icbH_mut.pdb')

# Load PRE data
rawData = dataparse.read_pre('../data_files/calbindin_Tb_N_R1_600.pre')
```

(continues on next page)

(continued from previous page)

```
# Parse PRE data
data = prot.parse(rawData)
```

The Tb tensor fitted from PCS data is loaded and the relevant parameters, in this case the magnetic field strength, temperature and rotational correlation time are set.

```
met = metal.load_tensor('../data_files/calbindin_Tb_HN_PCS_tensor.txt')
met.B0 = 14.1
met.T = 298.0
met.taur = 4.25E-9
```

A loop is conducted over the nitrogen atoms that are present in the experimental data. The PRE is calculated using the function `paramagpy.metal.atom_pre()`. Calculations without CSA are appended to the list `cal` and calculations including CSA cross-correlation with the Curie-spin relaxation are appended to the list `cal_csa`.

```
exp = []
cal = []
cal_csa = []
for atom, pre, err in data[['atm','exp','err']]:
        exp.append(pre)
        cal.append(met.atom_pre(atom, rtype='r1'))
        cal_csa.append(met.atom_pre(atom, rtype='r1', csa=atom.csa))
```

Finally the data are plotted. Clearly CSA cross-correlation is a big effect for backbone nitrogen atoms and should always be taken into account for Curie-spin calculations. Also note the existence and correct prediction of negative PREs!

```
from matplotlib import pyplot as plt
fig, ax = plt.subplots(figsize=(5,5))

# Plot the data
ax.scatter(exp, cal, label="Standard Theory")
ax.scatter(exp, cal_csa, label="CSA x Curie spin")

# Plot a diagonal
l, h = ax.get_xlim()
ax.plot([l,h],[l,h],'grey',zorder=0)
ax.set_xlim(l,h)
ax.set_ylim(l,h)

# Make axis labels and save figure
ax.set_xlabel("Experiment")
ax.set_ylabel("Calculated")
ax.legend()
fig.savefig("pre_calc_nitrogen.png")
```

Output: [`pre_calc_nitrogen.png`]

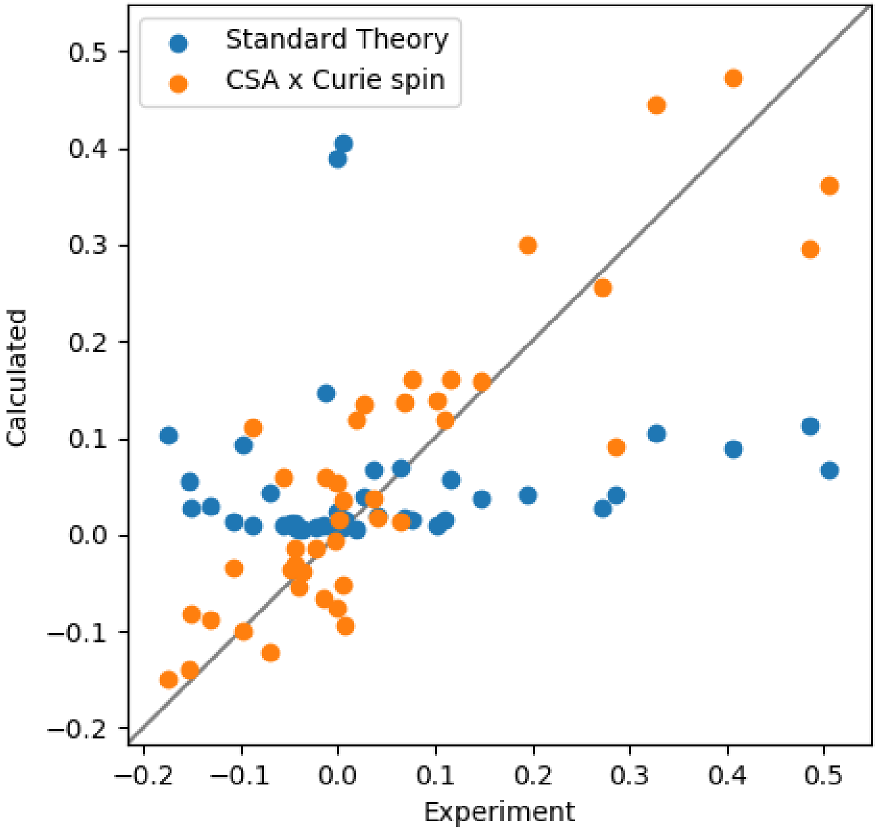

Fit spectral power density tensor

This example shows how to fit the spectral power density tensor to anisotropic PREs. The data and theory are derived from https://doi.org/10.1039/C8CP01332B.

Downloads

- Download the data files `parashift_Tb.pdb` and `parashift_Tb_R1_exp.pre` from here:
- Download the script `pre_fit_aniso_dipolar.py`

Script + Explanation

Load the relevant modules, read the PDB coordinates and experimental PRE values. Parse the values.

```
from paramagpy import protein, metal, fit, dataparse
from matplotlib import pyplot as plt
import numpy as np

prot = protein.load_pdb('../data_files/parashift_Tb.pdb')
pre_exp = dataparse.read_pre('../data_files/parashift_Tb_R1_exp.pre')
exp = prot.parse(pre_exp)
```

The spectral power density tensor is written here explicitly and set to the attribute `g_tensor`. The values here are sourced from the original paper, and arise from the robust linear fit to the experimental data. We will use this tensor for comparison to the fit achieved by paramagpy.

```
m = metal.Metal(taur=0.42E-9, B0=1.0, temperature=300.0)
m.set_lanthanide('Tb')

m.g_tensor = np.array([
        [1754.0, -859.0, -207.0],
        [-859.0, 2285.0, -351.0],
        [-207.0, -351.0, -196.0]]) * 1E-60
```

An starting tensor with no parameters is also initialised and will be used for fitting to the exerimental data with paramagpy.

```
m0 = metal.Metal(taur=0.42E-9, B0=1.0, temperature=300.0)
m0.set_lanthanide('Tb')
```

The fit is conducted by setting the `usegsbm` flag to `True`. This uses anisotropic SBM theory to fit the spectral power density tensor in place of the isotropic SBM theory. The relevant fitting parameters must be specified as `'t1e', 'gax', 'grh', 'a','b','g'` which represent the electronic relaxation time, the axial and rhombic componenets of the power spectral density tensor and the 3 Euler angles alpha, beta and gamma respectively. Note that the fitted `t1e` parameter is only an estimate of the electronic relaxation time.

```
[mfit], [data] = fit.nlr_fit_metal_from_pre([m0], [exp], params=('t1e', 'gax', 'grh
↪', 'a','b','g'),
```

Finally the results of the fit are plotted alongside the isotropic theory and the literature fit. Note that the difference in the fit from paramagpy is small, and probably arises because the original paper uses a *Robust* linear fit, which may include weighting with experimental uncertainties. However paramagpy weights values evely here because the experimental uncertainties are unknown.

```
pos = np.array([a.position for a in exp['atm']])
gam = np.array([a.gamma for a in exp['atm']])

fig = plt.figure(figsize=(5,5))
ax = fig.add_subplot(111)
ax.plot([0,3200],[0,3200], '-k')
ax.plot(exp['exp'], mfit.fast_sbm_r1(pos, gam), marker='o', lw=0, label='iso')
ax.plot(exp['exp'], mfit.fast_g_sbm_r1(pos, gam), marker='o', lw=0, label='aniso')
ax.plot(exp['exp'], m.fast_g_sbm_r1(pos, gam), marker='o', lw=0, label='literature␣
↪fit')
ax.set_xlim(0,3200)
ax.set_ylim(0,3200)
ax.set_xlabel("R1 experimental /Hz")
ax.set_ylabel("R1 calcualted /Hz")
ax.set_title("Tb parashift R1")
ax.legend()
fig.tight_layout()
fig.savefig("pre_fit_aniso_dipolar.png")
```

Output: `[pre_fit_aniso_dipolar.png]`

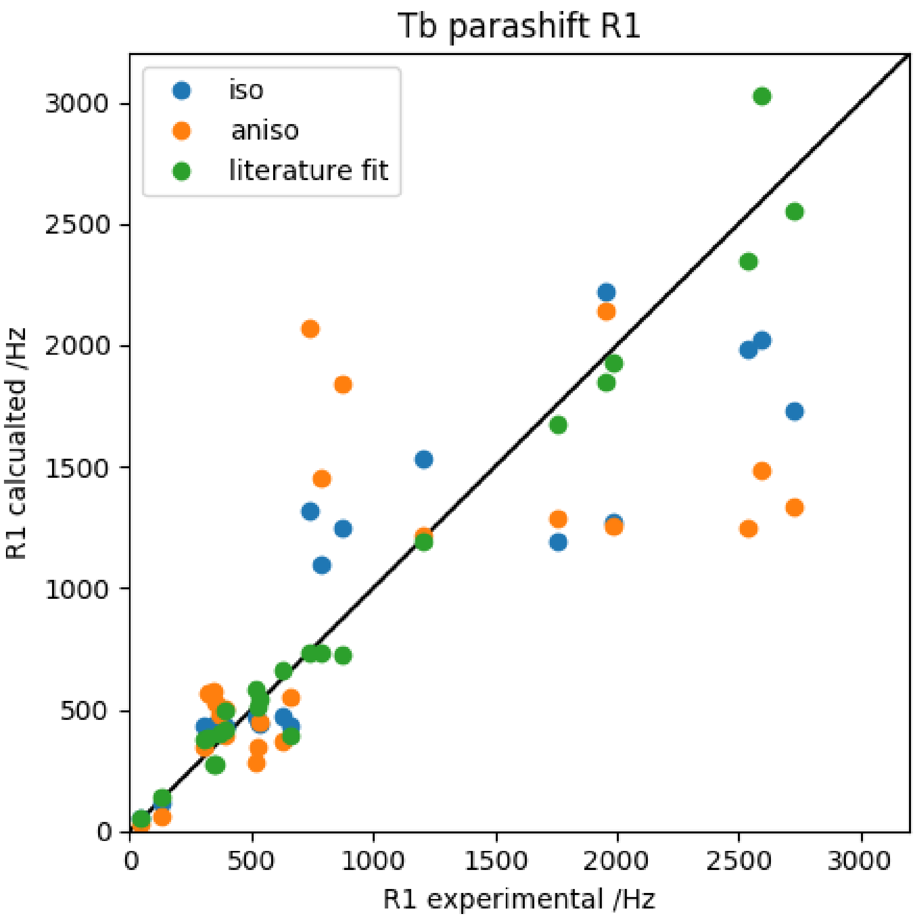

5.2.4 CCR data

Calculate Cross-correlated Relaxation

This example shows how to calculate dipole-dipole/Curie-spin cross-correlated relaxation as measured for data in the literature by Pintacuda et. al.

Downloads

- Download the data files `1bzrH.pdb`, `myoglobin_cn.ccr` and `myoglobin_f.ccr` from here:
- Download the script `ccr_calculate.py`

Script + Explanation

First the relevant modules are loaded, and the iron atom (paramagnetic centre) is identified as the variable `ironAtom`.

```
from paramagpy import protein, fit, dataparse, metal
import numpy as np

# Load the PDB file and get iron centre
prot = protein.load_pdb('../data_files/1bzrH.pdb')
ironAtom = prot[0]['A'][("H_HEM",154," ")]['FE']
```

Two paramagnetic centres are defined for the high and low spin iron atom. The positions are set to that of the iron centre along with other relevant parameters. The measured isotropic χ-tensor magnitudes are also set.

```
met_cn = metal.Metal(position=ironAtom.position,
                                                 B0=18.79,
                                                 temperature=303.0,
                                                 taur=5.7E-9)
met_f = met_cn.copy()
met_cn.iso = 4.4E-32
met_f.iso = 30.1E-32
```

The experimental data are loaded and parsed by the protein.

```
data_cn = prot.parse(dataparse.read_ccr("../data_files/myoglobin_cn.ccr"))
data_f = prot.parse(dataparse.read_ccr("../data_files/myoglobin_f.ccr"))
```

A loop is conducted over the atoms contained in the experimental data and the CCR rate is calculated using the function *paramagpy.metal.Metal.atom_ccr()*. These are appended to lists `compare_cn` and `compare_f`.

Note that the two H and N atoms are provided. The first atom is the nuclear spin undergoing active relaxation. The second atom is the coupling partner. Thus by swapping the H and N atoms to give `atom_ccr(N, H)`, the differential line broadening can be calculated in the indirect dimension.

```
# Calculate the cross-correlated realxation
compare_cn = []
for H, N, value, error in data_cn[['atm','atx','exp','err']]:
        delta = met_cn.atom_ccr(H, N)
        compare_cn.append((value, delta*0.5))

compare_f = []
for H, N, value, error in data_f[['atm','atx','exp','err']]:
        delta = met_f.atom_ccr(H, N)
        compare_f.append((value, delta*0.5))
```

Finally a correlation plot is made.

```
#### Plot the correlation ####
from matplotlib import pyplot as plt
fig, ax = plt.subplots(figsize=(5,5))

# Plot the data correlations
ax.scatter(*zip(*compare_cn), s=7, label="myo_cn")
ax.scatter(*zip(*compare_f), s=7, label="myo_f")

# Plot a diagonal
l, h = ax.get_xlim()
ax.plot([l,h],[l,h],'-k',zorder=0)
ax.set_xlim(l,h)
ax.set_ylim(l,h)

# Make axis labels and save figure
ax.set_xlabel("Experiment")
ax.set_ylabel("Calculated")
ax.legend()
fig.savefig("ccr_calculate.png")
```

Output: [`ccr_calculate.png`]

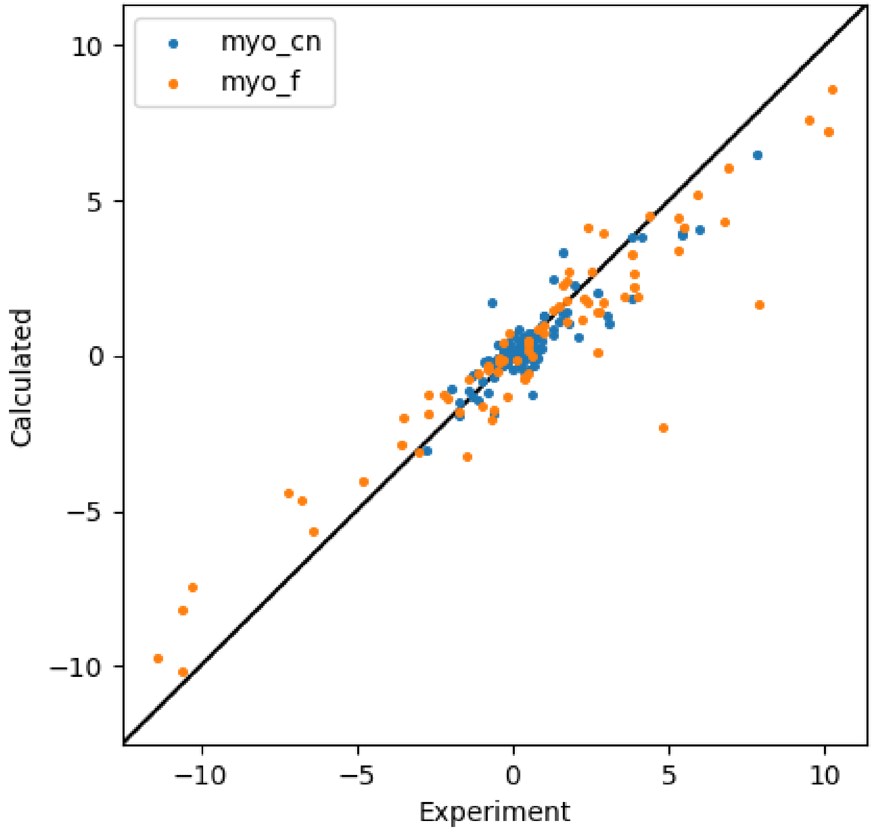

5.3 Graphic User Interface (GUI)

Paramagpy is equipped with a GUI which is cross-platform and contains most of the functionality of the scripted module. This gives a rapid way for new users to fit and compare PCS, RDC and PRE effects.

5.3.1 YouTube Tutorial

Check out the tutorial on YouTube

5.3.2 Running the GUI

To run the GUI, first open the python inperpreter in the terminal

```
user@computer:~$ python3
Python 3.5.2 (default, Nov 23 2017, 16:37:01)
[GCC 5.4.0 20160609] on linux
Type "help", "copyright", "credits" or "license" for more information.
>>>
```

Then import paramagpy and start the gui with `paramagpy.gui.run()`.

```
user@computer:~$ python3
Python 3.5.2 (default, Nov 23 2017, 16:37:01)
[GCC 5.4.0 20160609] on linux
```

(continues on next page)

(continued from previous page)

```
Type "help", "copyright", "credits" or "license" for more information.
>>> import paramagpy
>>> paramagpy.gui.run()
```

Alternatively you can simply execute the following from the command line

```
user@computer:~$ echo "import paramagpy; paramagpy.gui.run()" | python3
```

If all this fails, you can contact the author for a prebuilt executable at henry.orton@anu.edu.au

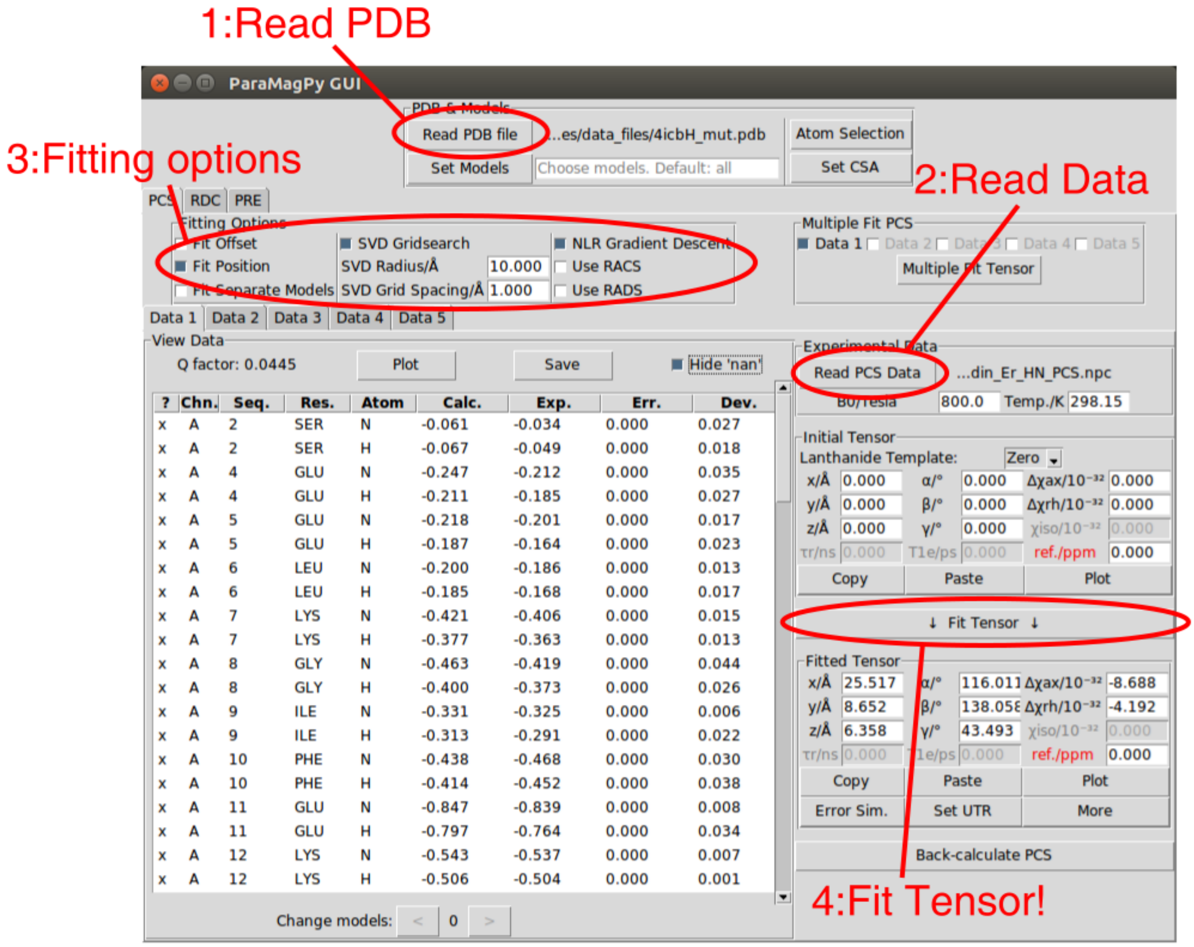

5.4 NMR Software Macros

Paramagpy includes scripts for reading/writing PCS values directly from popular NMR software. This drastically improves the iterative process of tensor fitting.

5.4.1 CCPNMR Analysis 2.4

Download the two scripts:

- `paramagpy_ccpnmr_macro.py`
- `paramagpy_fit_pcs.py`

In the first line of the script *paramagpy_fit_pcs.py*, replace the shebang with the path to the python version on your machine that contains the paramagpy installation. On my computer this is set to.

```
#!/usr/bin/python3
```

Open CCPNMR analysis and navigate to Menu->Macro->Organise Macros. At the lower right click *Add Macro* and open the script *paramagpy_ccpnmr_macro.py*, then select *paramagpyMACRO* and click *Load Macro*. You can then select if from the list and click *Run* to reveal the screen below.

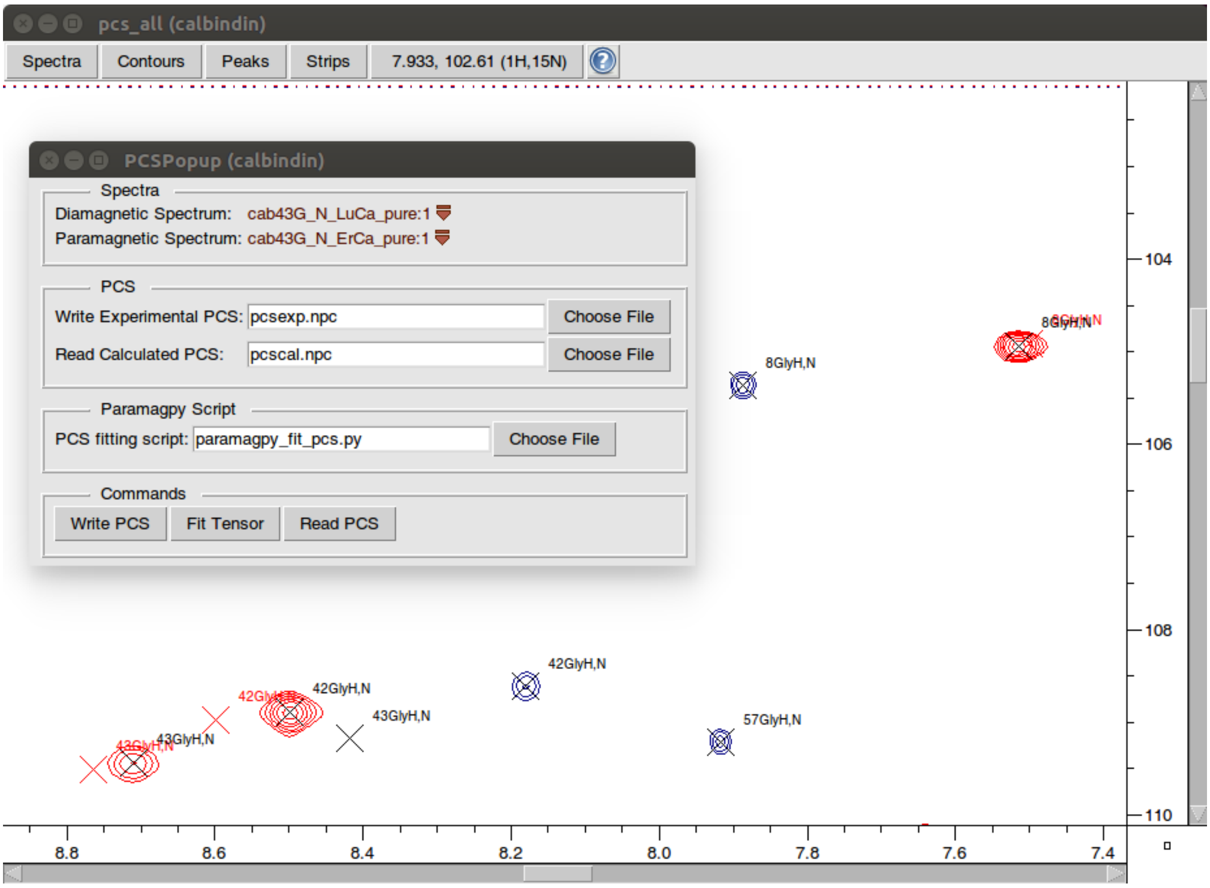

The popup window allows you to select a diamagnetic and paramagnetic spectrum and has 3 available buttons:

- Write PCS: This will calculate the difference between assigned peaks in the paramagnetic and diamagnetic spectra and write them to a .npc file (as specified in the relevant field).
- Fit Tensor: This will call the paramagpy script *paramagpy_fit_pcs.py* to fit the tensor the the written PCS values.
- Read PCS: This will read back-calculated PCS values from file (as specified in the relevant field) and plot the expected peaks on the paramagnetic spectrum in red.

Note, to alter the parameters for fitting of the PCS tensor, you can change the values within the script *paramagpy_fit_pcs.py*.

5.4.2 Sparky

Download the 3 scripts:

- `paramagpy_sparky_macro.py`
- `sparky_init.py`
- `paramagpy_fit_pcs.py`

Place the first two scripts *paramagpy_sparky_macro.py* and *sparky_init.py* in the Sparky directory *~/Sparky/Python*. Note that the Sparky directory usually contains the *Projects*, *Lists* and *Save* folders. You may need to create the *Python* directory here in which to place the two scripts.

Place the third script *paramagpy_fit_pcs.py* in your home directory.

Open Sparky and navigate to Extensions->Read and write PCS files.

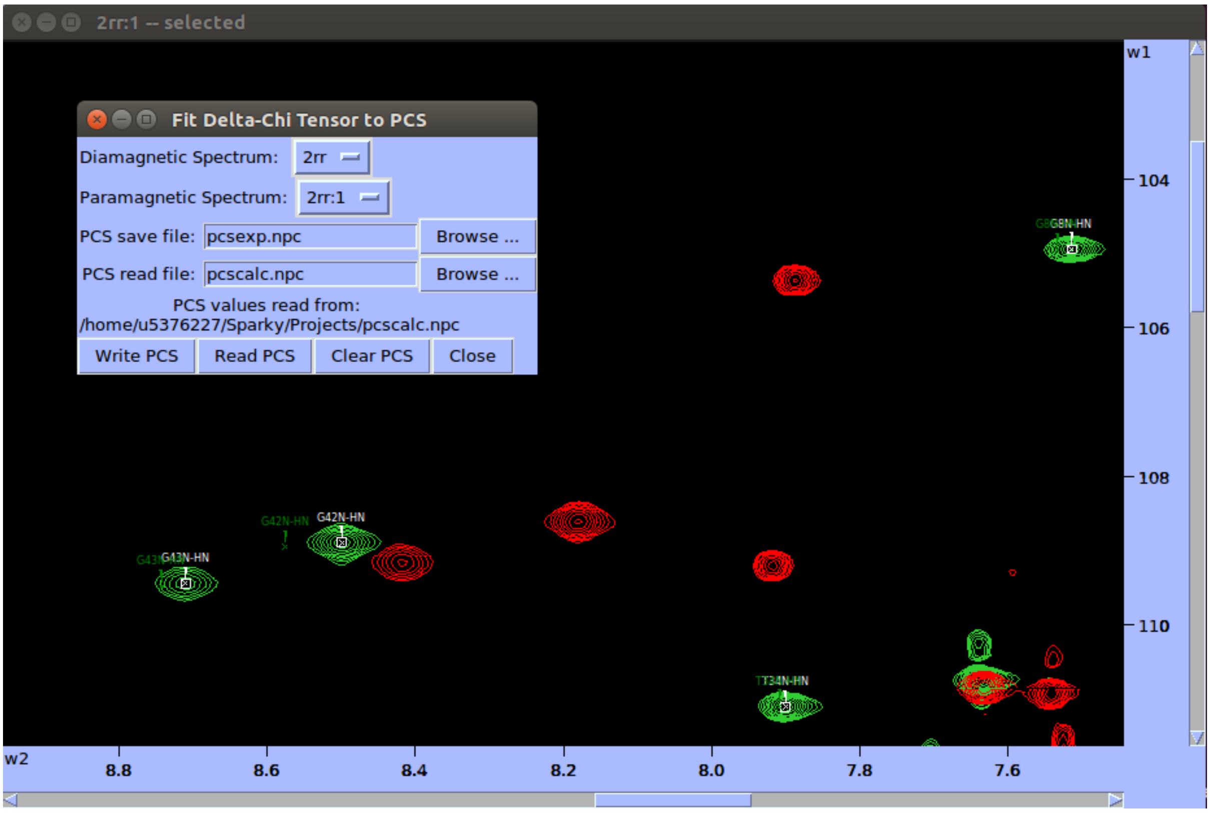

The popup window allows you to select a diamagnetic and paramagnetic spectrum and has 3 available buttons:

- Write PCS: This will calculate the difference between assigned peaks in the paramagnetic and diamagnetic spectra and write them to a .npc file (as specified in the relevant field).
- Read PCS: This will read back-calculated PCS values from file (as specified in the relevant field) and plot the expected peaks on the paramagnetic spectrum in green.
- Clear PCS: This will remove all calculated PCS peaks from the spectrum.

Note, to perform the tensor fitting, you will need to execute the paramagpy script in a separate terminal including an argument with the experimental PCS file such as:

```
user@computer:~$ ./paramagpy_fit_pcs.py pcsexp.npc
```

To alter the parameters for fitting of the PCS tensor, you can change the values within the script *paramagpy_fit_pcs.py*.

5.5 Mathematical Proofs

5.5.1 Proof of equivalence between the matrix representation of Curie-spin dipole–dipole cross-correlated relaxation (Paramagpy implementation) and the original description by Ghose and Prestegard

This proof concerns equations 24-27 of the article on the software Paramagpy, which describe the cross-correlated relaxation between the Curie spin and dipole–dipole relaxation mechanisms. The equations 24-27 follow the matrix representation of relaxation theory (equations 20-21) by Suturina et al. . It is shown below that these equations are equivalent to the equations established previously by Ghose and Prestegard and reported in slightly modified form by Bertini et al.. The proof begins with equations 24-27 of the manuscript and describes their rearrangement into the form given by Bertini et al.

For a specific example, we consider the case of a ^{15}N - ^{1}H group, with the Curie-spin shielding tensor σ at the site of the ^{1}H spin located at $\vec{r} = [x, y, z]$ and distance r from the paramagnetic centre.

$$\sigma = \frac{1}{4\pi r^5} \begin{bmatrix} (3x^2 - r^2) & 3xy & 3xz \\ 3xy & (3y^2 - r^2) & 3yz \\ 3xz & 3yz & (3z^2 - r^2) \end{bmatrix} \cdot \begin{bmatrix} \chi_{xx} & \chi_{xy} & \chi_{xz} \\ \chi_{xy} & \chi_{yy} & \chi_{yz} \\ \chi_{xz} & \chi_{yz} & \chi_{zz} \end{bmatrix}$$

We choose the orientation of the electron–^{1}H vector to be aligned with the z-axis such that $z \rightarrow r$. In the case of an isotropic magnetic susceptibility, $x = y = 0$ and the χ tensor is represented by a diagonal matrix with three identical elements χ_{iso}, yielding the following simplification

$$\begin{aligned} \sigma &= \frac{1}{4\pi r^5} \begin{bmatrix} -r^2 & 0 & 0 \\ 0 & -r^2 & 0 \\ 0 & 0 & 2r^2 \end{bmatrix} \cdot \begin{bmatrix} \chi_{\text{iso}} & 0 & 0 \\ 0 & \chi_{\text{iso}} & 0 \\ 0 & 0 & \chi_{\text{iso}} \end{bmatrix} \\ &= \xi_{\text{DSA}} \begin{bmatrix} -r^2 & 0 & 0 \\ 0 & -r^2 & 0 \\ 0 & 0 & 2r^2 \end{bmatrix} \\ \text{where} \qquad \xi_{\text{DSA}} &= \frac{\chi_{\text{iso}}}{4\pi r^5} \end{aligned}$$

The nuclear dipole shielding tensor arising from the ^{15}N spin can be described in the same coordinate frame for an arbitrary orientation of the bond vector $\vec{r}_{\text{HN}} = [x, y, z]$ with bond length r_{HN} by

$$\begin{aligned} \sigma_N &= \frac{1}{B_0} \frac{\mu_0}{4\pi} \gamma_N \hbar I \left[3 \frac{\vec{r}_{\text{HN}} \otimes \vec{r}_{\text{HN}}^T}{r_{\text{HN}}^5} - \frac{\mathbb{I}_3}{r_{\text{HN}}^3} \right] \\ \sigma_N &= \xi_{\text{DD}} \begin{bmatrix} (3x^2 - r_{\text{HN}}^2) & 3xy & 3xz \\ 3xy & (3y^2 - r_{\text{HN}}^2) & 3yz \\ 3xz & 3yz & (3z^2 - r_{\text{HN}}^2) \end{bmatrix} \\ \text{where} \qquad \xi_{\text{DD}} &= \frac{1}{B_0} \frac{\mu_0}{4\pi} \frac{\gamma_N \hbar I}{r_{\text{HN}}^5} \end{aligned}$$

and x, y, z denote the coordinates of the ^{15}N spin relative to the ^{1}H spin.

The effective shielding tensor at the site of the ^{1}H spin, when the ^{15}N partner is in the spin-up state, is given by the sum of the two tensors

$$\begin{aligned} \sigma_\uparrow &= \sigma + \sigma_N \\ &= \begin{bmatrix} (3x^2 - r_{\text{HN}}^2)\xi_{\text{DD}} - (r^2)\xi_{\text{DSA}} & (3xy)\xi_{\text{DD}} & (3xz)\xi_{\text{DD}} \\ (3xy)\xi_{\text{DD}} & (3y^2 - r_{\text{HN}}^2)\xi_{\text{DD}} - (r^2)\xi_{\text{DSA}} & (3yz)\xi_{\text{DD}} \\ (3xz)\xi_{\text{DD}} & (3yz)\xi_{\text{DD}} & (3z^2 - r_{\text{HN}}^2)\xi_{\text{DD}} + (2r^2)\xi_{\text{DSA}} \end{bmatrix} \end{aligned}$$

Note that this matrix is symmetric. Therefore we can ignore equation 18 of the main text and only need to substitute matrix elements into equation 19. Expanding and simplifying (via symbolic processing in the program

Mathematica), this yields

$$\begin{aligned}
\Delta(\sigma_\uparrow)^2 &= \sigma_{xx}^2 + \sigma_{yy}^2 + \sigma_{zz}^2 - \sigma_{xx}\sigma_{yy} - \sigma_{xx}\sigma_{zz} - \sigma_{yy}\sigma_{zz} \\
&\quad + \frac{3}{4}\left[((\sigma_{xy}+\sigma_{yx})^2 + (\sigma_{xz}+\sigma_{zx})^2 + (\sigma_{yz}+\sigma_{zy})^2\right] \\
&= \left((3x^2 - r_{\text{HN}}^2)\xi_{\text{DD}} - (r^2)\xi_{\text{DSA}}\right)^2 \\
&\quad + \left((3y^2 - r^2)\xi_{\text{DD}} - (r^2)\xi_{\text{DSA}}\right)^2 \\
&\quad + \left((3z^2 - r_{\text{HN}}^2)\xi_{\text{DD}} + (2r^2)\xi_{\text{DSA}}\right)^2 \\
&\quad - \left((3x^2 - r_{\text{HN}}^2)\xi_{\text{DD}} - (r^2)\xi_{\text{DSA}}\right)\left((3y^2 - r^2)\xi_{\text{DD}} - (r^2)\xi_{\text{DSA}}\right) \\
&\quad - \left((3x^2 - r_{\text{HN}}^2)\xi_{\text{DD}} - (r^2)\xi_{\text{DSA}}\right)\left((3z^2 - r_{\text{HN}}^2)\xi_{\text{DD}} + (2r^2)\xi_{\text{DSA}}\right) \\
&\quad - \left((3y^2 - r^2)\xi_{\text{DD}} - (r^2)\xi_{\text{DSA}}\right)\left((3z^2 - r_{\text{HN}}^2)\xi_{\text{DD}} + (2r^2)\xi_{\text{DSA}}\right) \\
&\quad + \frac{3}{4}\left[(6xy\xi_{\text{DD}})^2 + (6xz\xi_{\text{DD}})^2 + (6yz\xi_{\text{DD}})^2\right] \\
&= 9\xi_{\text{DD}}^2 r_{\text{HN}}^4 + 9\xi_{\text{DSA}}^2 r^4 - 9\xi_{\text{DD}}(r_{\text{HN}}^2 - 3z^2)\xi_{\text{DSA}} r^2
\end{aligned}$$

The angle θ between the electron-nuclear vector $\vec{r}$ and the nuclear bond vector $\vec{r}_{\text{HN}}$ is captured by the dot product formula

$$\begin{aligned}
\vec{r}.\vec{r}_{\text{HN}} &= |\vec{r}||\vec{r}_{\text{HN}}|\cos\theta \\
\implies \qquad [0,0,r].[x,y,z] &= r.r_{\text{HN}}\cos\theta \\
\implies \qquad z &= r_{\text{HN}}\cos\theta
\end{aligned}$$

Using above equation to substitute \$z\$ yields

$$\Delta(\sigma_\uparrow)^2 = 9\xi_{\text{DD}}^2 r_{\text{HN}}^4 + 9\xi_{\text{DSA}}^2 r^4 - 9\xi_{\text{DD}} r_{\text{HN}}^2(1 - 3\cos^2\theta)\xi_{\text{DSA}} r^2$$

where the first two terms account for the dipolar and Curie spin auto-relaxation terms respectively, and the last term accounts for their cross-correlation. The R_2 relaxation rate can be calculated by substitution of Δ^2 into equation 21 of the main text.

$$R_2^{\text{Curie}}(\sigma_\uparrow) = \frac{1}{45}\omega^2\left[4\mathbf{J}(0) + 3\mathbf{J}(\omega)\right]\left(9\xi_{\text{DD}}^2 r_{\text{HN}}^4 + 9\xi_{\text{DSA}}^2 r^4 + 9\xi_{\text{DD}} r_{\text{HN}}^2(3\cos^2\theta - 1)\xi_{\text{DSA}} r^2\right)$$

The same derivation for $\sigma_\downarrow$ yields the same result except for a sign change in the cross term:

$$R_2^{\text{Curie}}(\sigma_\downarrow) = \frac{1}{45}B_0^2\gamma_H^2\left[4\mathbf{J}(0) + 3\mathbf{J}(\omega)\right]\left(9\xi_{\text{DD}}^2 r_{\text{HN}}^4 + 9\xi_{\text{DSA}}^2 r^4 - 9\xi_{\text{DD}} r_{\text{HN}}^2(3\cos^2\theta - 1)\xi_{\text{DSA}} r^2\right)$$

Taking the difference we obtain

$$\begin{aligned}
R_2^{\text{Curie}\times\text{DD}} &= R_2^{\text{Curie}}(\sigma_\uparrow) - R_2^{\text{Curie}}(\sigma_\downarrow) \\
&= \frac{1}{45}B_0^2\gamma_H^2\left(18\xi_{\text{DD}} r_{\text{HN}}^2(3\cos^2\theta - 1)\xi_{\text{DSA}} r^2\right)\left[4\mathbf{J}(0) + 3\mathbf{J}(\omega)\right] \\
&= \frac{18}{45}\frac{\mu_0}{4\pi}\frac{B_0\gamma_H^2\gamma_N\hbar I}{r_{\text{HN}}^3}\frac{\chi_{\text{iso}}}{4\pi r^3}(3\cos^2\theta - 1)\left[4\mathbf{J}(0) + 3\mathbf{J}(\omega)\right]
\end{aligned}$$

Substituting χ_{iso} and the spin of ^{15}N as $^{\text{I=1/2}}$ yields

$$\begin{aligned}
R_2^{\text{Curie}\times\text{DD}} &= \frac{9}{45}\left(\frac{\mu_0}{4\pi}\right)^2\frac{B_0\gamma_H^2\gamma_N\hbar}{r_{\text{HN}}^3}\frac{\mu_B^2 g^2 J(J+1)}{3kTr^3}(3\cos^2\theta - 1)\left[4\mathbf{J}(0) + 3\mathbf{J}(\omega)\right] \\
&= \frac{2}{15}\left(\frac{\mu_0}{4\pi}\right)^2\frac{B_0\gamma_H^2\gamma_N\hbar}{r_{\text{HN}}^3}\frac{\mu_B^2 g^2 J(J+1)}{kTr^3}\frac{(3\cos^2\theta - 1)}{2}\left[4\mathbf{J}(0) + 3\mathbf{J}(\omega)\right]
\end{aligned}$$

The differential line width can be calculated from the relaxation rate as $\Delta\nu = R_2^{\text{Curie}\times\text{DD}}/\pi$ and thus this equation matches equation 7 from Bertini et al..

5.6 Reference Guide

5.6.1 Paramagnetic module

This module handles the paramagnetic centre by defining the magnetic susceptibility tensor and methods for PCS, RDC and PRE calculations.

paramagpy.metal

Functions

euler_to_matrix(eulers)	Calculate a rotation matrix from euler angles using ZYZ convention
matrix_to_euler(M)	Calculate Euler angles from a rotation matrix using ZYZ convention
unique_eulers(eulers)	Calculate Euler angles in unique tensor representation.
make_tensor(x, y, z, axial, rhombic, alpha, . . .)	Make a ChiTensor isntance from given parameters.

paramagpy.metal.euler_to_matrix

paramagpy.metal.**euler_to_matrix**(*eulers*)

Calculate a rotation matrix from euler angles using ZYZ convention

Parameters **eulers** (*array of floats*) – the euler angles [alpha,beta,gamma] in radians by ZYZ convention.

Returns **matrix** – the rotation matrix

Return type 3x3 numpy ndarray

Examples

```
>>> eulers = np.array([0.5,1.2,0.8])
>>> euler_to_matrix(eulers)
array([[-0.1223669 , -0.5621374 ,  0.81794125],
       [ 0.75057357,  0.486796  ,  0.44684334],
       [-0.64935788,  0.66860392,  0.36235775]])
```

paramagpy.metal.matrix_to_euler

paramagpy.metal.**matrix_to_euler**(*M*)

Calculate Euler angles from a rotation matrix using ZYZ convention

Parameters **M** (*3x3 numpy ndarray*) – a rotation matrix

Returns **eulers** – the euler angles [alpha,beta,gamma] in radians by ZYZ convention

Return type array of floats

Examples

```
>>> matrix = array([[-0.1223669 , -0.5621374 ,  0.81794125],
                    [ 0.75057357,  0.486796  ,  0.44684334],
                    [-0.64935788,  0.66860392,  0.36235775]])
>>> matrix_to_euler(matrix)
np.array([0.5,1.2,0.8])
```

paramagpy.metal.unique_eulers

paramagpy.metal.**unique_eulers**(*eulers*)

Calculate Euler angles in unique tensor representation.

Given general Euler angles by ZYZ convention, this function accounts for the symmetry of a second rank symmetric tensor to map all angles within the range [0, pi].

Parameters **eulers** (*array of float*) – the three Euler angles in radians

Returns **eulers_utr** – the euler angles [alpha,beta,gamma] in radians by ZYZ convention

Return type array of floats

Examples

```
>>> eulers = np.array([-5.2,10.3,0.1])
>>> unique_eulers(eulers)
np.array([1.08318531 0.87522204 3.04159265])
```

paramagpy.metal.make_tensor

paramagpy.metal.**make_tensor**(*x*, *y*, *z*, *axial*, *rhombic*, *alpha*, *beta*, *gamma*, *lanthanide=None*, *temperature=298.15*)

Make a ChiTensor isntance from given parameters. This is designed to use pdb coordinates (x, y, z) and euler angles from an output like Numbat.

Parameters

- **y, z** (*x,*) – tensor position in pdb coordiante in Angstroms
- **rhombic** (*axial,*) – the tensor anisotropies in units 10^-32
- **beta, gamma** (*alpha,*) – the euler angles in degrees that maps the tensor to the pdb (I think?)

Returns **ChiTensor** – a tensor object for calulating paramagnetic effects on nuclear spins in the pdb coordinate

Return type object *paramagpy.metal.Metal*

Classes

Metal([position, eulers, axrh, mueff, . . .])	An object for paramagnetic chi tensors and delta-chi tensors.

paramagpy.metal.Metal

class paramagpy.metal.**Metal**(*position=(0, 0, 0)*, *eulers=(0, 0, 0)*, *axrh=(0, 0)*, *mueff=0.0*, *g_axrh=(0, 0)*, *t1e=0.0*, *shift=0.0*, *temperature=298.15*, *B0=18.79*, *taur=0.0*)

An object for paramagnetic chi tensors and delta-chi tensors. This class has basic attributes that specify position, axiality/rhombicity, isotropy and euler angles. It also has methods for calculating PCS, RDC, PRE and CCR values.

__init__(*position=(0, 0, 0)*, *eulers=(0, 0, 0)*, *axrh=(0, 0)*, *mueff=0.0*, *g_axrh=(0, 0)*, *t1e=0.0*, *shift=0.0*, *temperature=298.15*, *B0=18.79*, *taur=0.0*)

Instantiate ChiTensor object

Parameters

- **position** (*array of floats, optional*) – the (x,y,z) position in meters. Default is (0,0,0) stored as a np.matrix object.
- **eulers** (*array of floats, optional*) – the euler angles [alpha,beta,gamma] in radians by ZYZ convention. Defualt is (0,0,0)
- **axrh** (*array of floats, optional*) – the axial and rhombic values defining the magnetic susceptibility anisotropy
- **g_axrh** (*array of floats, optional*) – the axial and rhombic values defining the power spectral density tensor
- **mueff** (*float*) – the effective magnetic moment in units of A.m^2
- **shift** (*float*) – a bulk shift value applied to all PCS calculations. This is a correction parameter that may arise due to an offset between diamagnetic and paramagnetic PCS datasets.
- **temperature** (*float*) – the temperature in Kelvin
- **t1e** (*float*) – the longitudinal electronic relaxation time
- **B0** (*float*) – the magnetic field in Telsa
- **taur** (*float*) – the rotational correlation time in seconds

Methods

atom_ccr(atom, atomPartner)	Calculate R2 cross-corelated relaxation due to DDxDSA
atom_pcs(atom[, racs, rads])	Calculate the psuedo-contact shift at the given atom
atom_pre(atom[, rtype, dsa, sbm, csa])	Calculate the PRE for an atom
atom_rdc(atom1, atom2)	Calculate the residual dipolar coupling between two atoms
atom_set_position(atom)	Set the position of the Metal object to that of an atom
average(metals)	Set the attributes of the current instance to the average of a list of provided tensor objects

Continued on next page

Table 3 – continued from previous page

ccr(position, gamma, dipole_shift_tensor)	Calculate R2 cross-corelated relaxation due to DDxDSA
copy()	Copy the current Metal object to a new instance
dipole_shift_tensor(position)	Calculate the chemical shift tensor at the given postition
dsa_r1(position, gamma[, csa])	Calculate R1 relaxation due to Curie Spin
dsa_r2(position, gamma[, csa])	Calculate R2 relaxation due to Curie Spin
fast_ccr(posarray, gammaarray, dstarray)	Vectorised version of paramagpy.metal.Metal.ccr()
fast_dipole_shift_tensor(posarray)	A vectorised version of paramagpy.metal.Metal.dipole_shift_tensor()
fast_dsa_r1(posarray, gammaarray[, csaarray])	Vectorised version of paramagpy.metal.Metal.dsa_r1()
fast_dsa_r2(posarray, gammaarray[, csaarray])	Vectorised version of paramagpy.metal.Metal.dsa_r2()
fast_first_invariant_squared(t)	Vectorised version of paramagpy.metal.Metal.first_invariant_squared()
fast_g_sbm_r1(posarray, gammaarray)	Vectorised version of paramagpy.metal.Metal.g_sbm_r1()
fast_pcs(posarray)	A vectorised version of paramagpy.metal.Metal.pcs()
fast_pre(posarray, gammaarray, rtype[, dsa, ...])	Calculate the PRE for a set of spins using Curie and or SBM theory
fast_racs(csaarray)	A vectorised version of paramagpy.metal.Metal.racs()
fast_rads(posarray)	A vectorised version of paramagpy.metal.Metal.rads()
fast_rdc(vecarray, gammaProdArray)	A vectorised version of paramagpy.metal.Metal.rdc() method.
fast_sbm_r1(posarray, gammaarray)	Vectorised version of paramagpy.metal.Metal.sbm_r1()
fast_sbm_r2(posarray, gammaarray)	Vectorised version of paramagpy.metal.Metal.sbm_r2()
fast_second_invariant_squared(t)	Vectorised version of paramagpy.metal.Metal.second_invariant_squared()
first_invariant_squared(t)	Calculate the antisymmetric contribution to relaxation via the first invariant of a tensor.
g_sbm_r1(position, gamma)	Calculate R1 relaxation due to Solomon-Bloembergen-Morgan theory from anisotropic power spectral density tensor
get_params(params)	Get tensor parameters that have been scaled appropriately
info([comment])	Get basic information about the Metal object
isomap([protein, isoval])	
make_mesh([density, size])	Construct a 3D grid of points to map an isosurface
pcs(position)	Calculate the psuedo-contact shift at the given postition
pcs_mesh(mesh)	Calculate a PCS value at each location of cubic grid of points
pre(position, gamma, rtype[, dsa, sbm, ...])	Calculate the PRE for a set of spins using Curie and or SBM theory
pre_mesh(mesh[, gamma, rtype, dsa, sbm])	Calculate a PRE value at each location of cubic grid of points

Continued on next page

Table 3 – continued from previous page

racs(csa)	Calculate the residual anisotropic chemical shift at the given postition.
rads(position)	Calculate the residual anisotropic dipolar shift at the given postition.
rdc(vector, gammaProd)	Calculate Residual Dipolar Coupling (RDC)
save([fileName])	
sbm_r1(position, gamma)	Calculate R1 relaxation due to Solomon-Bloembergen-Morgan theory
sbm_r2(position, gamma)	Calculate R2 relaxation due to Solomon-Bloembergen-Morgan theory
second_invariant_squared(t)	Calculate the second invariant squared of a tensor.
set_Jg(J, g)	Set the magnetic susceptibility absolute magnitude from J/g.
set_lanthanide(lanthanide[, set_dchi])	Set the anisotropy, isotropy and T1e parameters from literature values
set_params(paramValues)	Set tensor parameters that have been scaled appropriately
set_utr()	Modify current tensor parameters to unique tensor representation (UTR)
spec_dens(tau, omega)	A spectral density function with Lorentzian shape:
write_isomap(mesh, bounds[, fileName])	Write a PyMol script to file which allows loading of the isosurface file
write_pymol_script([isoval, surfaceName, . . .])	Write a PyMol script to file which allows loading of the isosurface file

paramagpy.metal.Metal.atom_ccr

Metal.**atom_ccr**(*atom*, *atomPartner*)

Calculate R2 cross-corelated relaxation due to DDxDSA

Parameters

- **atom** (paramagpy.protein.CustomAtom) – the active nuclear spin for which relaxation will be calculated must have attributes 'position' and 'gamma'
- **atomPartner** (paramagpy.protein.CustomAtom) – the coupling parnter nuclear spin must have method 'dipole_shift_tensor'

Returns **value** – the CCR differential line broadening in Hz

Return type float

paramagpy.metal.Metal.atom_pcs

Metal.**atom_pcs**(*atom*, *racs=False*, *rads=False*)

Calculate the psuedo-contact shift at the given atom

Parameters

- **atom** (*biopython atom object*) – must have 'position' attribute
- **racs** (*bool (optional)*) – when True, RACS (residual anisotropic chemical shielding) correction is included. Default is False
- **rads** (*bool (optional)*) – when True, RADS (residual anisotropic dipolar shielding) correction is included. Defualt is False

Returns **pcs** – the pseudo-contact shift in parts-per-million (ppm)

Return type float

paramagpy.metal.Metal.atom_pre

`Metal.`**`atom_pre`**(*atom*, *rtype='r2'*, *dsa=True*, *sbm=True*, *csa=0.0*)

Calculate the PRE for an atom

Parameters

- **atom** (`paramagpy.protein.CustomAtom`) – the active nuclear spin for which relaxation will be calculated must have attributes 'position' and 'gamma'
- **rtype** (*str*) – either 'r1' or 'r2', the relaxation type
- **dsa** (*bool (optional)*) – when True (defualt), DSA or Curie spin relaxation is included
- **sbm** (*bool (optional)*) – when True (defualt), SBM spin relaxation is included
- **csa** (*array with shape (3,3) (optional)*) – CSA tensor of the spin. This defualts to 0.0, meaning CSAxDSA crosscorrelation is not accounted for.

Returns **rate** – The PRE rate in /s

Return type float

paramagpy.metal.Metal.atom_rdc

`Metal.`**`atom_rdc`**(*atom1*, *atom2*)

Calculate the residual dipolar coupling between two atoms

Parameters

- **atom1** (*biopython atom object*) – must have 'position' and 'gamma' attribute
- **atom1** – must have 'position' and 'gamma' attribute

Returns **rdc** – the RDC values in Hz

Return type float

paramagpy.metal.Metal.atom_set_position

`Metal.`**`atom_set_position`**(*atom*)

Set the position of the Metal object to that of an atom

Parameters **atom** (*biopython atom object*) – must have 'position' attribute

paramagpy.metal.Metal.average

`Metal.`**`average`**(*metals*)

Set the attributes of the current instance to the average of a list of provided tensor objects

WARNING: averging is unstable for spectral power density <g_tensor>

Parameters **metals** (*a list of Metal objects*) – the average of attributes of this list will be taken

paramagpy.metal.Metal.ccr

`Metal.`**`ccr`**(*position*, *gamma*, *dipole_shift_tensor*)

Calculate R2 cross-corelated relaxation due to DDxDSA

If the metal has an anisotropic magnetic susceptibility, this is taken into account.

Parameters

- **position** (*array of floats*) – three coordinates (x,y,z) this is the position of the nuclear spin
- **gamma** (*float*) – the gyromagnetic ratio of the relaxing spin
- **dipole_shift_tensor** (*3x3 array of floats*) – this is the dipole shift tensor arising from the nuclear spin of the coupling partner

Returns **value** – The R2 differential line broadening rate in /s

Return type float

paramagpy.metal.Metal.copy

`Metal.copy()`

Copy the current Metal object to a new instance

Returns **new_tensor** – a new Metal instance with the same parameters

Return type Metal object

paramagpy.metal.Metal.dipole_shift_tensor

`Metal.dipole_shift_tensor(position)`

Calculate the chemical shift tensor at the given postition

This arises due to the paramagnetic dipole tensor field

Parameters **position** (*array floats*) – the position (x, y, z) in meters

Returns **dipole_shift_tensor** – the tensor describing chemical shift at the nuclear position

Return type 3x3 array

paramagpy.metal.Metal.dsa_r1

`Metal.dsa_r1(position, gamma, csa=0.0)`

Calculate R1 relaxation due to Curie Spin

If the metal has an anisotropic magnetic susceptibility, this is taken into account, resulting in orientation dependent PRE as predicted by Vega and Fiat. CSA cross-correlated relaxation may be included by providing an appropriate CSA tensor.

Parameters

- **position** (*array of floats*) – three coordinates (x,y,z) in meters
- **gamma** (*float*) – the gyromagnetic ratio of the spin
- **csa** (*3x3 matrix (optional)*) – the CSA tensor of the given spin. This defualts to 0.0, meaning CSAxDSA crosscorrelation is not accounted for.

Returns **value** – The R1 relaxation rate in /s

Return type float

paramagpy.metal.Metal.dsa_r2

`Metal.dsa_r2(position, gamma, csa=0.0)`

Calculate R2 relaxation due to Curie Spin

If the metal has an anisotropic magnetic susceptibility, this is taken into account, resulting in orientation dependent PRE as predicted by Vega and Fiat. CSA cross-correlated relaxation may be included by providing an appropriate CSA tensor.

Parameters

- **position** (*array of floats*) – three coordinates (x,y,z)
- **gamma** (*float*) – the gyromagnetic ratio of the spin
- **csa** (*3x3 matrix (optional)*) – the CSA tensor of the given spin. This defualts to 0.0, meaning CSAxDSA crosscorrelation is not accounted for.

Returns **value** – The R2 relaxation rate in /s

Return type float

paramagpy.metal.Metal.fast_ccr

Metal.**fast_ccr**(*posarray*, *gammaarray*, *dstarray*)

Vectorised version of *paramagpy.metal.Metal.ccr()*

This is generally used for speed in fitting DDxDSA data

If the metal has an anisotropic magnetic susceptibility, this is taken into account.

Parameters

- **posarray** (*array with shape (n,3)*) – array of positions in meters
- **gammaarray** (*array with shape (n,3)*) – array of gyromagnetic ratios of the spins
- **dstarray** (*array with shape (n,3,3)*) – array of nuclear dipole shift tensors arising from the coupling partners

Returns **rates** – The R2 differential line broadening rates in /s

Return type array with shape (n,1)

paramagpy.metal.Metal.fast_dipole_shift_tensor

Metal.**fast_dipole_shift_tensor**(*posarray*)

A vectorised version of *paramagpy.metal.Metal.dipole_shift_tensor()*

This is generally used for fast calculations.

Parameters **posarray** (*array*) – an array of positions with shape (n,3)

Returns **dipole_shift_tensor_array** – and array of dipole shift tensors at corresponding positions. This has shape (n,3,3)

Return type array

paramagpy.metal.Metal.fast_dsa_r1

Metal.**fast_dsa_r1**(*posarray*, *gammaarray*, *csaarray=0.0*)

Vectorised version of *paramagpy.metal.Metal.dsa_r1()*

This is generally used for speed in fitting PRE data

Parameters

- **posarray** (*array with shape (n,3)*) – array of positions in meters
- **gammaarray** (*array with shape (n,3)*) – array of gyromagnetic ratios of the spins
- **csaarray** (*array with shape (m,3,3) (optional)*) – array of CSA tensors of the spins. This defualts to 0.0, meaning CSAxDSA crosscorrelation is not accounted for.

Returns **rates** – The R1 relaxation rates in /s

Return type array with shape (n,1)

paramagpy.metal.Metal.fast_dsa_r2

Metal.**fast_dsa_r2**(*posarray*, *gammaarray*, *csaarray=0.0*)

Vectorised version of *paramagpy.metal.Metal.dsa_r2()*

This is generally used for speed in fitting PRE data.

Parameters

- **posarray** (*array with shape (n,3)*) – array of positions in meters
- **gammaarray** (*array with shape (n,3)*) – array of gyromagnetic ratios of the spins
- **csaarray** (*array with shape (m,3,3) (optional)*) – array of CSA tensors of the spins. This defualts to 0.0, meaning CSAxDSA crosscorrelation is not accounted for.

Returns **rates** – The R2 relaxation rates in /s
Return type array with shape (n,1)

paramagpy.metal.Metal.fast_first_invariant_squared

static `Metal.`**`fast_first_invariant_squared`**(*t*)
Vectorised version of *`paramagpy.metal.Metal.first_invariant_squared()`*

This is generally used for speed in fitting PRE data
Parameters **`tensorarray`** (*`array with shape (n,3,3)`*) – array of shielding tensors
Returns **firstInvariantSquared** – the first invariants squared of the tensors
Return type array with shape (n,1)

paramagpy.metal.Metal.fast_g_sbm_r1

`Metal.`**`fast_g_sbm_r1`**(*posarray*, *gammaarray*)
Vectorised version of *`paramagpy.metal.Metal.g_sbm_r1()`*

This is generally used for speed in fitting PRE data
Parameters
- **`posarray`** (*`array with shape (n,3)`*) – array of positions in meters
- **`gammaarray`** (*`array with shape (n,3)`*) – array of gyromagnetic ratios of the spins

Returns **rates** – The R1 relaxation rates in /s
Return type array with shape (n,1)

paramagpy.metal.Metal.fast_pcs

`Metal.`**`fast_pcs`**(*posarray*)
A vectorised version of *`paramagpy.metal.Metal.pcs()`*

This efficient algorithm calculates the PCSs for an array of positions and is best used where speed is required for fitting.
Parameters **`posarray`** (*`array with shape (n,3)`*) – array of 'n' positions (x, y, z) in meters
Returns **pcs** – the peudo-contact shift in parts-per-million (ppm)
Return type array of floats with shape (n,1)

paramagpy.metal.Metal.fast_pre

`Metal.`**`fast_pre`**(*posarray*, *gammaarray*, *rtype*, *dsa=True*, *sbm=True*, *gsbm=False*, *csaarray=0.0*)
Calculate the PRE for a set of spins using Curie and or SBM theory
Parameters
- **`posarray`** (*`array with shape (n,3)`*) – array of positions in meters
- **`gammaarray`** (*`array with shape (n,3)`*) – array of gyromagnetic ratios of the spins
- **`rtype`** (*`str`*) – either 'r1' or 'r2', the relaxation type
- **`dsa`** (*`bool (optional)`*) – when True (defualt), DSA or Curie spin relaxation is included
- **`sbm`** (*`bool (optional)`*) – when True (defualt), SBM spin relaxation is included
- **`gsbm`** (*`bool (optional)`*) – when True (default=False), anisotropic dipolar relaxation is included using the spectral power density gensor <g_tensor> NOTE:

when true, ignores relaxation of type SBM NOTE: only implemented for R1 relaxation calculations
- **csaarray** (*array with shape (m,3,3) (optional)*) – array of CSA tensors of the spins. This defualts to 0.0, meaning CSAxDSA crosscorrelation is not accounted for.

Returns **rates** – The PRE rates in /s

Return type array with shape (n,1)

paramagpy.metal.Metal.fast_racs

`Metal.`**`fast_racs`**(*csaarray*)

A vectorised version of *paramagpy.metal.Metal.racs()*

This is generally used when speed is required for fitting

Parameters **csaarray** (*array with shape (n,3,3)*) – array of chemical shift anisotropy tensors

Returns **racs_array** – the residual anisotropic chemical shift in parts-per-million (ppm)

Return type array of floats with shape (n,1)

paramagpy.metal.Metal.fast_rads

`Metal.`**`fast_rads`**(*posarray*)

A vectorised version of *paramagpy.metal.Metal.rads()*

This is generally used when speed is required for fitting

Parameters **posarray** (*array with shape (n,3)*) – an array of 'n' positions (x, y, z) in meters

Returns **rads_array** – the residual anisotropic dipole shift in parts-per-million (ppm)

Return type array of floats with shape (n,1)

paramagpy.metal.Metal.fast_rdc

`Metal.`**`fast_rdc`**(*vecarray*, *gammaProdArray*)

A vectorised version of *paramagpy.metal.Metal.rdc()* method.

This is generally used for speed in fitting RDC data

Parameters
- **vecarray** (*array with shape (n,3)*) – array of internuclear vectors in meters
- **gammaProdArray** (*array with shape (n,1)*) – the products of gyromagnetic ratios of spins A and B where each has units of rad/s/T

Returns **rdc_array** – the RDC values in Hz

Return type array with shape (n,1)

paramagpy.metal.Metal.fast_sbm_r1

`Metal.`**`fast_sbm_r1`**(*posarray*, *gammaarray*)

Vectorised version of *paramagpy.metal.Metal.sbm_r1()*

This is generally used for speed in fitting PRE data

Parameters
- **posarray** (*array with shape (n,3)*) – array of positions in meters
- **gammaarray** (*array with shape (n,3)*) – array of gyromagnetic ratios of the spins

Returns **rates** – The R1 relaxation rates in /s

Return type array with shape (n,1)

paramagpy.metal.Metal.fast_sbm_r2

`Metal.fast_sbm_r2(posarray, gammaarray)`

Vectorised version of *paramagpy.metal.Metal.sbm_r2()*

This is generally used for speed in fitting PRE data

Parameters

- **posarray** (*array with shape (n,3)*) – array of positions in meters
- **gammaarray** (*array with shape (n,3)*) – array of gyromagnetic ratios of the spins

Returns **rates** – The R2 relaxation rates in /s

Return type array with shape (n,1)

paramagpy.metal.Metal.fast_second_invariant_squared

`static Metal.fast_second_invariant_squared(t)`

Vectorised version of *paramagpy.metal.Metal.second_invariant_squared()*

This is generally used for speed in fitting PRE data

Parameters **tensorarray** (*array with shape (n,3,3)*) – array of shielding tensors

Returns **secondInvariantSquared** – the second invariants squared of the tensors

Return type array with shape (n,1)

paramagpy.metal.Metal.first_invariant_squared

`static Metal.first_invariant_squared(t)`

Calculate the antisymmetric contribution to relaxation via the first invariant of a tensor.

This is required for PRE calculations using the shilding tensor

Parameters **tensor** (*3x3 matrix*) – a second rank tensor

Returns **firstInvariantSquared** – the first invariant squared of the shift tensor

Return type float

paramagpy.metal.Metal.g_sbm_r1

`Metal.g_sbm_r1(position, gamma)`

Calculate R1 relaxation due to Solomon-Bloembergen-Morgan theory from anisotropic power spectral density tensor

Parameters

- **position** (*array of floats*) – three coordinates (x,y,z)
- **gamma** (*float*) – the gyromagnetic ratio of the spin

Returns **value** – The R1 relaxation rate in /s

Return type float

paramagpy.metal.Metal.get_params

`Metal.get_params(params)`

Get tensor parameters that have been scaled appropriately

This is often used to get parameter values during fitting where floating point errors would otherwise occur on the small values encountered.

Parameters **params** (*list of str*) – each element of the list is a string that corresponds to an attribute of the Metal to be retrieved.

Returns **scaled_params** – a list with respective scaled parameter values from the input.

Return type list

Examples

```
>>> metal = Metal(axrh=[20E-32, 3E-32],position=[0.0,10E-10,-5E-10])
>>> metal.get_params(['ax','rh','x','y','z'])
[20.0, 3.0, 0.0, 10.0, -5.0]
```

paramagpy.metal.Metal.info

`Metal.info(comment=True)`

Get basic information about the Metal object

This is returned as a string in human readable units This is also the file format for saving the tensor

Parameters **comment** (*bool (optional)*) – if True, each line has a '#' placed at the front

Returns **information** – a string containing basic information about the Metal

Return type str

Examples

```
>>> metal = Metal()
>>> metal.set_lanthanide('Er')
>>> metal.info()
# ax    | 1E-32 m^3 :   -11.600
# rh    | 1E-32 m^3 :    -8.600
# x     |   1E-10 m :     0.000
# y     |   1E-10 m :     0.000
# z     |   1E-10 m :     0.000
# a     |       deg :     0.000
# b     |       deg :     0.000
# g     |       deg :     0.000
# mueff |        Bm :     9.581
# shift |       ppm :     0.000
# B0    |         T :    18.790
# temp  |         K :   298.150
# t1e   |        ps :     0.189
# taur  |        ns :     0.000
```

paramagpy.metal.Metal.isomap

`Metal.isomap(protein=None, isoval=1.0, **kwargs)`

paramagpy.metal.Metal.make_mesh

`Metal.make_mesh(density=2, size=40.0)`

Construct a 3D grid of points to map an isosurface

This is contained in a cube

Parameters

- **density** (*int (optional)*) – the points per Angstrom in the grid
- **size** (*float (optional)*) – the length of one edge of the cube

Returns

- **mesh** (*cubic grid array*) – This has shape (n,n,n,3) where n is the number of points along one edge of the grid. Units are meters
- **origin** (*array of floats,*) – the (x,y,z) location of mesh vertex
- **low** (*array of ints, the integer location of the first*) – point in each dimension
- **high** (*array of ints, the integer location of the last*) – point in each dimension
- **points** (*array of ints,*) – the number of points along each dimension

paramagpy.metal.Metal.pcs

`Metal.pcs(position)`

Calculate the psuedo-contact shift at the given postition

Parameters **position** (*array floats*) – the position (x, y, z) in meters

Returns **pcs** – the pseudo-contact shift in parts-per-million (ppm)

Return type float

Examples

```
>>> metal = Metal()
>>> metal.set_lanthanide('Er')
>>> metal.pcs([0.,0.,10E-10])
-6.153991132886608
```

paramagpy.metal.Metal.pcs_mesh

`Metal.pcs_mesh(mesh)`

Calculate a PCS value at each location of cubic grid of points

Parameters **mesh** (*array with shape (n,n,n,3)*) – a cubic grid as generated by the method <make_mesh>

Returns **pcs_mesh** – The same grid shape, with PCS values at the respective locations

Return type array with shape (n,n,n,1)

paramagpy.metal.Metal.pre

`Metal.pre` (*position*, *gamma*, *rtype*, *dsa=True*, *sbm=True*, *gsbm=False*, *csa=0.0*)

Calculate the PRE for a set of spins using Curie and or SBM theory

Parameters

- **position** (*array of floats*) – position in meters
- **gamma** (*float*) – gyromagnetic ratio of the spin
- **rtype** (*str*) – either 'r1' or 'r2', the relaxation type
- **dsa** (*bool (optional)*) – when True (defualt), DSA or Curie spin relaxation is included
- **sbm** (*bool (optional)*) – when True (defualt), SBM spin relaxation is included
- **gsbm** (*bool (optional)*) – when True (default=False), anisotropic dipolar relaxation is included using the spectral power density gensor <g_tensor> NOTE: when true, ignores relaxation of type SBM NOTE: only implemented for R1 relaxation calculations
- **csa** (*array with shape (3,3) (optional)*) – CSA tensor of the spin. This defualts to 0.0, meaning CSAxDSA crosscorrelation is not accounted for.

Returns **rate** – The PRE rate in /s

Return type float

paramagpy.metal.Metal.pre_mesh

`Metal.pre_mesh` (*mesh*, *gamma=267512897.63847807*, *rtype='r2'*, *dsa=True*, *sbm=True*)

Calculate a PRE value at each location of cubic grid of points

Parameters

- **mesh** (*array with shape (n,n,n,3)*) – a cubic grid as generated by the method <make_mesh>
- **gamma** (*float*) – the gyromagnetic ratio of the spin
- **rtype** (*str*) – either 'r1' or 'r2', the relaxation type
- **dsa** (*bool (optional)*) – when True (defualt), DSA or Curie spin relaxation is included
- **sbm** (*bool (optional)*) – when True (defualt), SBM spin relaxation is included

Returns **pre_mesh** – The same grid shape, with PRE values at the respective locations

Return type array with shape (n,n,n,1)

paramagpy.metal.Metal.racs

`Metal.racs` (*csa*)

Calculate the residual anisotropic chemical shift at the given postition.

The partial alignment induced by an anisotropic magnetic susecptiblity causes the chemical shift tensor at a nuclear position to average to a value different to the isotropic value.

Parameters **csa** (*3 x 3 array*) – the chemical shift anisotropy tensor

Returns **racs** – the residual anisotropic chemical shift in parts-per-million (ppm)

Return type float

paramagpy.metal.Metal.rads

`Metal.rads(position)`

Calculate the residual anisotropic dipolar shift at the given postition.

The partial alignment induced by an anisotropic magnetic susecptiblity causes the dipole shift tensor at a nuclear position to average to a value different to the PCS.

Parameters **position** (*array floats*) – the position (x, y, z) in meters

Returns **rads** – the residual anisotropic dipole shift in parts-per-million (ppm)

Return type float

paramagpy.metal.Metal.rdc

`Metal.rdc(vector, gammaProd)`

Calculate Residual Dipolar Coupling (RDC)

Parameters

- **vector** (*array of floats*) – internuclear vector (x,y,z) in meters
- **gammaProd** (*float*) – the product of gyromagnetic ratios of spin A and B where each has units of rad/s/T

Returns **rdc** – the RDC in Hz

Return type float

paramagpy.metal.Metal.save

`Metal.save(fileName='tensor.txt')`

paramagpy.metal.Metal.sbm_r1

`Metal.sbm_r1(position, gamma)`

Calculate R1 relaxation due to Solomon-Bloembergen-Morgan theory

Parameters

- **position** (*array of floats*) – three coordinates (x,y,z)
- **gamma** (*float*) – the gyromagnetic ratio of the spin

Returns **value** – The R1 relaxation rate in /s

Return type float

paramagpy.metal.Metal.sbm_r2

`Metal.sbm_r2(position, gamma)`

Calculate R2 relaxation due to Solomon-Bloembergen-Morgan theory

Parameters

- **position** (*array of floats*) – three coordinates (x,y,z)
- **gamma** (*float*) – the gyromagnetic ratio of the spin

Returns **value** – The R2 relaxation rate in /s

Return type float

paramagpy.metal.Metal.second_invariant_squared

static Metal.**second_invariant_squared**(*t*)

Calculate the second invariant squared of a tensor.

This is required for PRE calculations using the shilding tensor

Parameters **tensor** (*3x3 matrix*) – a second rank tensor

Returns **secondInvariantSquared** – the second invariant squared of the shift tensor

Return type float

paramagpy.metal.Metal.set_Jg

Metal.**set_Jg**(*J*, *g*)

Set the magnetic susceptibility absolute magnitude from J/g.

This is achieved using the following formula:

$$\mu_{eff} = g\mu_B\sqrt{J(J+1)}$$

Parameters

- **J** (*str*) – the total spin angular momentum quantum number
- **g** (*bool, optional*) – the Lande g-factor

paramagpy.metal.Metal.set_lanthanide

Metal.**set_lanthanide**(*lanthanide*, *set_dchi=True*)

Set the anisotropy, isotropy and T1e parameters from literature values

Parameters

- **lanthanide** (*str*) – one of 'Ce','Pr','Nd','Pm','Sm','Eu','Gd','Tb', 'Dy','Ho','Er','Tm','Yb'
- **set_dchi** (*bool (optional)*) – if True (default), the tensor anisotropy is set. Otherwise only the isotropy and T1e values are set

paramagpy.metal.Metal.set_params

Metal.**set_params**(*paramValues*)

Set tensor parameters that have been scaled appropriately

This is the inverse of the method <get_params>

Parameters **paramValues** (*list of tuple*) – each element is a tuple (variable, value) where 'variable' is the string indentifying the attribute to be set, and 'value' is the corresponding value

Examples

```
>>> metal = Metal()
>>> metal.set_params([('ax',20.0),('rh',3.0)])
>>> metal.axrh
[2.e-31 3.e-32]
```

paramagpy.metal.Metal.set_utr

Metal.**set_utr**()

Modify current tensor parameters to unique tensor representation (UTR)

Note that multiple axial/rhombic and euler angles can give congruent tensors. This method ensures that identical tensors may always be compared by using Numbat style representation.

paramagpy.metal.Metal.spec_dens

static Metal.**spec_dens**(*tau*, *omega*)

A spectral density function with Lorentzian shape:

$$\mathbb{J}(\tau,\omega) = \frac{\tau}{1+(\omega\tau)^2}$$

Parameters
- **tau** (*float*) – correaltion time
- **omega** (*float*) – frequency

Returns **value** – the value of the spectral denstiy

Return type float

paramagpy.metal.Metal.write_isomap

Metal.**write_isomap**(*mesh*, *bounds*, *fileName='isomap.pml.ccp4'*)

Write a PyMol script to file which allows loading of the isosurface file

Parameters
- **mesh** (*3D scalar np.ndarray of floats*) – the scalar field of PCS or PRE values in a cubic grid
- **bounds** (*tuple (origin, low, high, points)*) – as generated by *paramagpy.metal.Metal.make_mesh()*
- **fileName** (*str (optional)*) – the filename of the isosurface file

paramagpy.metal.Metal.write_pymol_script

Metal.**write_pymol_script**(*isoval=1.0*, *surfaceName='isomap'*, *scriptName='isomap.pml'*, *meshName='./isomap.pml.ccp4'*, *pdbFile=None*)

Write a PyMol script to file which allows loading of the isosurface file

Parameters
- **isoval** (*float (optional)*) – the contour level of the isosurface
- **surfaceName** (*str (optional)*) – the name of the isosurface file within PyMol
- **scriptName** (*str (optional)*) – the name of the PyMol script to load the tensor isosurface
- **meshName** (*str (optional)*) – the name of the binary isosurface file
- **pdbFile** (*str (optional)*) – if not <None>, the file name of the PDB file to be loaded with the isosurface.

Attributes

B0_MHz	1H NMR frequency for the given field in MHz
GAMMA	
HBAR	
K	
MU0	
MUB	
a	alpha euler anglue
alignment_factor	Factor for conversion between magnetic susceptibility and alignment tensors
ax	axiality
b	beta euler anglue
eigenvalues	The eigenvalues defining the magnitude of the principle axes
fit_scaling	
fundamental_attributes	
g	gamma euler anglue
g_eigenvalues	The eigenvalues defining the magnitude of the principle axes
g_isotropy	Estimate of the spectral power density tensor isotropy
g_tensor	The magnetic susceptibility tensor matrix representation
gax	axial componenet of spectral power density tensor
grh	axial componenet of spectral power density tensor
iso	isotropy
isotropy	The magnidue of the isotropic component of the tensor
lanth_axrh	
lanth_lib	
lower_coords	
rh	rhombicity
rotationMatrix	The rotation matrix as defined by the euler angles
saupe_factor	Factor for conversion between magnetic susceptibility and saupe tensors
tauc	The effective rotational correlation time.
tensor	The magnetic susceptibility tensor matrix representation
tensor_alignment	The alignment tensor matrix representation
tensor_saupe	The saupe tensor matrix representation
tensor_traceless	The traceless magnetic susceptibility tensor matrix representation
upper_coords	
upper_triang	Fetch 5 unique matrix element defining the magnetic susceptibility tensor
upper_triang_alignment	Fetch 5 unique matrix element defining the alignment tensor
upper_triang_saupe	Fetch 5 unique matrix element defining the magnetic susceptibility tensor
x	x coordinate
y	y coordinate

Continued on next page

Table 4 – continued from previous page

z	z coordinate

paramagpy.metal.Metal.B0_MHz

Metal.**B0_MHz**
1H NMR frequency for the given field in MHz

paramagpy.metal.Metal.GAMMA

Metal.**GAMMA = 176085964400.0**

paramagpy.metal.Metal.HBAR

Metal.**HBAR = 1.0546e-34**

paramagpy.metal.Metal.K

Metal.**K = 1.381e-23**

paramagpy.metal.Metal.MU0

Metal.**MU0 = 1.2566370614359173e-06**

paramagpy.metal.Metal.MUB

Metal.**MUB = 9.274e-24**

paramagpy.metal.Metal.a

Metal.**a**
alpha euler anglue

paramagpy.metal.Metal.alignment_factor

Metal.**alignment_factor**
Factor for conversion between magnetic susceptibility and alignment tensors

paramagpy.metal.Metal.ax

Metal.**ax**
axiality

paramagpy.metal.Metal.b

`Metal.b`
beta euler anglue

paramagpy.metal.Metal.eigenvalues

`Metal.eigenvalues`
The eigenvalues defining the magnitude of the principle axes

paramagpy.metal.Metal.fit_scaling

`Metal.fit_scaling = {'a': 57.29577951308232, 'ax': 1e+32, 'b': 57.29577951308`

paramagpy.metal.Metal.fundamental_attributes

`Metal.fundamental_attributes = ('position', 'eulers', 'axrh', 'mueff', 'g_axrh',`

paramagpy.metal.Metal.g

`Metal.g`
gamma euler anglue

paramagpy.metal.Metal.g_eigenvalues

`Metal.g_eigenvalues`
The eigenvalues defining the magnitude of the principle axes

paramagpy.metal.Metal.g_isotropy

`Metal.g_isotropy`
Estimate of the spectral power density tensor isotropy

paramagpy.metal.Metal.g_tensor

`Metal.g_tensor`
The magnetic susceptibility tensor matrix representation

paramagpy.metal.Metal.gax

`Metal.gax`
axial componenet of spectral power density tensor

paramagpy.metal.Metal.grh

`Metal.grh`
axial componenet of spectral power density tensor

paramagpy.metal.Metal.iso

`Metal.iso`
isotropy

paramagpy.metal.Metal.isotropy

`Metal.isotropy`
The magnidue of the isotropic component of the tensor

paramagpy.metal.Metal.lanth_axrh

`Metal.lanth_axrh = {'Ce': (2.1, 0.7), 'Dy': (34.7, 20.3), 'Er': (-11.6, -8.6)`

paramagpy.metal.Metal.lanth_lib

`Metal.lanth_lib = {'Ce': (2.5, 0.8571428571428571, 1.33e-13), 'Dy': (7.5, 1.33`

paramagpy.metal.Metal.lower_coords

`Metal.lower_coords = ((0, 1, 1, 2, 2), (0, 1, 0, 0, 1))`

paramagpy.metal.Metal.rh

`Metal.rh`
rhombicity

paramagpy.metal.Metal.rotationMatrix

`Metal.rotationMatrix`
The rotation matrix as defined by the euler angles

paramagpy.metal.Metal.saupe_factor

`Metal.saupe_factor`
Factor for conversion between magnetic susceptibility and saupe tensors

paramagpy.metal.Metal.tauc

`Metal.tauc`

The effective rotational correlation time.

This is calculated by combining the rotational correaltion time and the electronic relaxation time:

$$\tau_c = \frac{1}{\frac{1}{\tau_r} + \frac{1}{T_{1e}}}$$

paramagpy.metal.Metal.tensor

`Metal.tensor`

The magnetic susceptibility tensor matrix representation

paramagpy.metal.Metal.tensor_alignment

`Metal.tensor_alignment`

The alignment tensor matrix representation

paramagpy.metal.Metal.tensor_saupe

`Metal.tensor_saupe`

The saupe tensor matrix representation

paramagpy.metal.Metal.tensor_traceless

`Metal.tensor_traceless`

The traceless magnetic susceptibility tensor matrix representation

paramagpy.metal.Metal.upper_coords

`Metal.upper_coords = ((0, 1, 0, 0, 1), (0, 1, 1, 2, 2))`

paramagpy.metal.Metal.upper_triang

`Metal.upper_triang`

Fetch 5 unique matrix element defining the magnetic susceptibility tensor

paramagpy.metal.Metal.upper_triang_alignment

`Metal.upper_triang_alignment`

Fetch 5 unique matrix element defining the alignment tensor

paramagpy.metal.Metal.upper_triang_saupe

Metal.**upper_triang_saupe**
Fetch 5 unique matrix element defining the magnetic susceptibility tensor

paramagpy.metal.Metal.x

Metal.**x**
x coordinate

paramagpy.metal.Metal.y

Metal.**y**
y coordinate

paramagpy.metal.Metal.z

Metal.**z**
z coordinate

5.6.2 Protein module

This module handles the protein structure coordinates and includes methods for loading a PDB file and calculating atomic properites such as CSA or gyromagnetic ratio

paramagpy.protein

Functions

load_pdb(fileName[, ident])	Read PDB from file into biopython structure object
rotation_matrix(axis, theta)	Return the rotation matrix associated with counter-clockwise rotation about the given axis by theta radians.

paramagpy.protein.load_pdb

paramagpy.protein.**load_pdb**(*fileName*, *ident=None*)
Read PDB from file into biopython structure object

Parameters

- **fileName** (*str*) – the path to the file
- **ident** (*str (optional)*) – the desired identity of the structure object

Returns **values** – a structure object containing the atomic coordinates

Return type *paramagpy.protein.CustomStructure*

paramagpy.protein.rotation_matrix

paramagpy.protein.**rotation_matrix**(*axis*, *theta*)

Return the rotation matrix associated with counterclockwise rotation about the given axis by theta radians.

Parameters **axis** (*array of floats*) – the [x,y,z] axis for rotation.

Returns **matrix** – the rotation matrix

Return type numpy 3x3 matrix object

Classes

CustomAtom(*arg, **kwargs)	
CustomStructure(*arg, **kwargs)	This is an overload hack of the BioPython Structure object
CustomStructureBuilder(*arg, **kwargs)	This is an overload hack of BioPython's CustomStructureBuilder

paramagpy.protein.CustomAtom

class paramagpy.protein.**CustomAtom**(**arg*, ***kwargs*)

__init__(**arg*, ***kwargs*)

Initialize Atom object.

Parameters

- **name** (*string*) – atom name (eg. "CA"). Note that spaces are normally stripped.
- **coord** (*Numeric array (Float0, size 3)*) – atomic coordinates (x,y,z)
- **bfactor** (*number*) – isotropic B factor
- **occupancy** (*number*) – occupancy (0.0-1.0)
- **altloc** (*string*) – alternative location specifier for disordered atoms
- **fullname** (*string*) – full atom name, including spaces, e.g. " CA ". Normally these spaces are stripped from the atom name.
- **element** (*uppercase string (or None if unknown)*) – atom element, e.g. "C" for Carbon, "HG" for mercury,

Methods

copy()	Create a copy of the Atom.
detach_parent()	Remove reference to parent.
dipole_shift_tensor(position)	Calculate the magnetic field shielding tensor at the given postition due to the nuclear dipole
flag_disorder()	Set the disordered flag to 1.
get_altloc()	Return alternative location specifier.
get_anisou()	Return anisotropic B factor.
get_bfactor()	Return B factor.
get_coord()	Return atomic coordinates.
get_full_id()	Return the full id of the atom.

Continued on next page

Table 7 – continued from previous page

get_fullname()	Return the atom name, including leading and trailing spaces.
get_id()	Return the id of the atom (which is its atom name).
get_level()	Return level.
get_name()	Return atom name.
get_occupancy()	Return occupancy.
get_parent()	Return parent residue.
get_serial_number()	Return the serial number.
get_sigatm()	Return standard deviation of atomic parameters.
get_siguij()	Return standard deviations of anisotropic temperature factors.
get_vector()	Return coordinates as Vector.
is_disordered()	Return the disordered flag (1 if disordered, 0 otherwise).
set_altloc(altloc)	Set alternative location specifier.
set_anisou(anisou_array)	Set anisotropic B factor.
set_bfactor(bfactor)	Set isotroptic B factor.
set_coord(coord)	Set coordinates.
set_occupancy(occupancy)	Set occupancy.
set_parent(parent)	Set the parent residue.
set_serial_number(n)	Set serial number.
set_sigatm(sigatm_array)	Set standard deviation of atomic parameters.
set_siguij(siguij_array)	Set standard deviations of anisotropic temperature factors.
top()	
transform(rot, tran)	Apply rotation and translation to the atomic coordinates.

paramagpy.protein.CustomAtom.copy

CustomAtom.**copy**()

Create a copy of the Atom.

Parent information is lost.

paramagpy.protein.CustomAtom.detach_parent

CustomAtom.**detach_parent**()

Remove reference to parent.

paramagpy.protein.CustomAtom.dipole_shift_tensor

CustomAtom.**dipole_shift_tensor**(*position*)

Calculate the magnetic field shielding tensor at the given postition due to the nuclear dipole

Assumes nuclear spin 1/2

Parameters **position** (*array floats*) – the position (x, y, z) in meters

Returns **dipole_shielding_tensor** – the tensor describing magnetic shielding at the given position

Return type 3x3 array

paramagpy.protein.CustomAtom.flag_disorder

`CustomAtom.flag_disorder()`

Set the disordered flag to 1.

The disordered flag indicates whether the atom is disordered or not.

paramagpy.protein.CustomAtom.get_altloc

`CustomAtom.get_altloc()`

Return alternative location specifier.

paramagpy.protein.CustomAtom.get_anisou

`CustomAtom.get_anisou()`

Return anisotropic B factor.

paramagpy.protein.CustomAtom.get_bfactor

`CustomAtom.get_bfactor()`

Return B factor.

paramagpy.protein.CustomAtom.get_coord

`CustomAtom.get_coord()`

Return atomic coordinates.

paramagpy.protein.CustomAtom.get_full_id

`CustomAtom.get_full_id()`

Return the full id of the atom.

The full id of an atom is the tuple (structure id, model id, chain id, residue id, atom name, altloc).

paramagpy.protein.CustomAtom.get_fullname

`CustomAtom.get_fullname()`

Return the atom name, including leading and trailing spaces.

paramagpy.protein.CustomAtom.get_id

`CustomAtom.get_id()`

Return the id of the atom (which is its atom name).

paramagpy.protein.CustomAtom.get_level

CustomAtom.**get_level**()
Return level.

paramagpy.protein.CustomAtom.get_name

CustomAtom.**get_name**()
Return atom name.

paramagpy.protein.CustomAtom.get_occupancy

CustomAtom.**get_occupancy**()
Return occupancy.

paramagpy.protein.CustomAtom.get_parent

CustomAtom.**get_parent**()
Return parent residue.

paramagpy.protein.CustomAtom.get_serial_number

CustomAtom.**get_serial_number**()
Return the serial number.

paramagpy.protein.CustomAtom.get_sigatm

CustomAtom.**get_sigatm**()
Return standard deviation of atomic parameters.

paramagpy.protein.CustomAtom.get_siguij

CustomAtom.**get_siguij**()
Return standard deviations of anisotropic temperature factors.

paramagpy.protein.CustomAtom.get_vector

CustomAtom.**get_vector**()
Return coordinates as Vector.

Returns coordinates as 3D vector

Return type Bio.PDB.Vector class

paramagpy.protein.CustomAtom.is_disordered

CustomAtom.**is_disordered**()

Return the disordered flag (1 if disordered, 0 otherwise).

paramagpy.protein.CustomAtom.set_altloc

CustomAtom.**set_altloc**(*altloc*)

Set alternative location specifier.

paramagpy.protein.CustomAtom.set_anisou

CustomAtom.**set_anisou**(*anisou_array*)

Set anisotropic B factor.

Parameters **anisou_array** (*Numeric array (length 6)*) – anisotropic B factor.

paramagpy.protein.CustomAtom.set_bfactor

CustomAtom.**set_bfactor**(*bfactor*)

Set isotroptic B factor.

paramagpy.protein.CustomAtom.set_coord

CustomAtom.**set_coord**(*coord*)

Set coordinates.

paramagpy.protein.CustomAtom.set_occupancy

CustomAtom.**set_occupancy**(*occupancy*)

Set occupancy.

paramagpy.protein.CustomAtom.set_parent

CustomAtom.**set_parent**(*parent*)

Set the parent residue.

Parameters **parent - Residue object** (*-*) –

paramagpy.protein.CustomAtom.set_serial_number

CustomAtom.**set_serial_number**(*n*)

Set serial number.

paramagpy.protein.CustomAtom.set_sigatm

CustomAtom.**set_sigatm**(*sigatm_array*)

Set standard deviation of atomic parameters.

The standard deviation of atomic parameters consists of 3 positional, 1 B factor and 1 occupancy standard deviation.

Parameters **sigatm_array** (*Numeric array (length 5)*) – standard deviations of atomic parameters.

paramagpy.protein.CustomAtom.set_siguij

CustomAtom.**set_siguij**(*siguij_array*)

Set standard deviations of anisotropic temperature factors.

Parameters **siguij_array** (*Numeric array (length 6)*) – standard deviations of anisotropic temperature factors.

paramagpy.protein.CustomAtom.top

CustomAtom.**top**()

paramagpy.protein.CustomAtom.transform

CustomAtom.**transform**(*rot*, *tran*)

Apply rotation and translation to the atomic coordinates.

Parameters

- **rot** (*3x3 Numeric array*) – A right multiplying rotation matrix
- **tran** (*size 3 Numeric array*) – the translation vector

Examples

This is an incomplete but illustrative example:

```
from numpy import pi, array
from Bio.PDB.vectors import Vector, rotmat
rotation = rotmat(pi, Vector(1, 0, 0))
translation = array((0, 0, 1), 'f')
atom.transform(rotation, translation)
```

Attributes

HBAR	
MU0	
csa	Get the CSA tensor at the nuclear position This uses the geometry of neighbouring atoms and a standard library from Bax J.
csa_lib	docstring for CustomAtom
gyro_lib	
position	

paramagpy.protein.CustomAtom.HBAR

`CustomAtom.HBAR = 1.0546e-34`

paramagpy.protein.CustomAtom.MU0

`CustomAtom.MU0 = 1.2566370614359173e-06`

paramagpy.protein.CustomAtom.csa

`CustomAtom.csa`

Get the CSA tensor at the nuclear position This uses the geometry of neighbouring atoms and a standard library from Bax J. Am. Chem. Soc. 2000

Returns matrix – the CSA tensor in the PDB frame if appropriate nuclear positions are not available <None> is returned.

Return type 3x3 array

paramagpy.protein.CustomAtom.csa_lib

`CustomAtom.csa_lib = {'C': (array([-8.65e-05, 1.18e-05, 7.47e-05]), 0.6632251157`

docstring for CustomAtom

paramagpy.protein.CustomAtom.gyro_lib

`CustomAtom.gyro_lib = {'C': 67261498.71335746, 'H': 267512897.63847807, 'N': -27`

paramagpy.protein.CustomAtom.position

`CustomAtom.position`

paramagpy.protein.CustomStructure

`class paramagpy.protein.CustomStructure(*arg, **kwargs)`

This is an overload hack of the BioPython Structure object

`__init__(*arg, **kwargs)`

Initialize the class.

Methods

add(entity)	Add a child to the Entity.
copy()	Copy entity recursively.
detach_child(id)	Remove a child.
detach_parent()	Detach the parent.
get_atoms()	Return atoms from residue.
get_chains()	Return chains from models.
get_full_id()	Return the full id.
get_id()	Return the id.
get_iterator()	Return iterator over children.
get_level()	Return level in hierarchy.

Continued on next page

Table 9 – continued from previous page

get_list()	Return a copy of the list of children.
get_models()	Return models.
get_parent()	Return the parent Entity object.
get_residues()	Return residues from chains.
has_id(id)	Check if a child with given id exists.
insert(pos, entity)	Add a child to the Entity at a specified position.
parse(dataValues[, models])	Associate experimental data with atoms of the PDB file This method takes a DataContainer instance from the dataparse module
set_parent(entity)	Set the parent Entity object.
transform(rot, tran)	Apply rotation and translation to the atomic coordinates.

paramagpy.protein.CustomStructure.add

`CustomStructure.`**`add`**`(`*entity*`)`
Add a child to the Entity.

paramagpy.protein.CustomStructure.copy

`CustomStructure.`**`copy`**`()`
Copy entity recursively.

paramagpy.protein.CustomStructure.detach_child

`CustomStructure.`**`detach_child`**`(`*id*`)`
Remove a child.

paramagpy.protein.CustomStructure.detach_parent

`CustomStructure.`**`detach_parent`**`()`
Detach the parent.

paramagpy.protein.CustomStructure.get_atoms

`CustomStructure.`**`get_atoms`**`()`
Return atoms from residue.

paramagpy.protein.CustomStructure.get_chains

`CustomStructure.`**`get_chains`**`()`
Return chains from models.

paramagpy.protein.CustomStructure.get_full_id

`CustomStructure.get_full_id()`

Return the full id.

The full id is a tuple containing all id's starting from the top object (Structure) down to the current object. A full id for a Residue object e.g. is something like:

("1abc", 0, "A", (" ", 10, "A"))

This corresponds to:

Structure with id "1abc" Model with id 0 Chain with id "A" Residue with id (" ", 10, "A")

The Residue id indicates that the residue is not a hetero-residue (or a water) because it has a blank hetero field, that its sequence identifier is 10 and its insertion code "A".

paramagpy.protein.CustomStructure.get_id

`CustomStructure.get_id()`

Return the id.

paramagpy.protein.CustomStructure.get_iterator

`CustomStructure.get_iterator()`

Return iterator over children.

paramagpy.protein.CustomStructure.get_level

`CustomStructure.get_level()`

Return level in hierarchy.

A - atom R - residue C - chain M - model S - structure

paramagpy.protein.CustomStructure.get_list

`CustomStructure.get_list()`

Return a copy of the list of children.

paramagpy.protein.CustomStructure.get_models

`CustomStructure.get_models()`

Return models.

paramagpy.protein.CustomStructure.get_parent

`CustomStructure.get_parent()`

Return the parent Entity object.

paramagpy.protein.CustomStructure.get_residues

`CustomStructure.get_residues()`
Return residues from chains.

paramagpy.protein.CustomStructure.has_id

`CustomStructure.has_id(id)`
Check if a child with given id exists.

paramagpy.protein.CustomStructure.insert

`CustomStructure.insert(pos, entity)`
Add a child to the Entity at a specified position.

paramagpy.protein.CustomStructure.parse

`CustomStructure.parse(dataValues, models=None)`
Associate experimental data with atoms of the PDB file This method takes a DataContainer instance from the dataparse module

Parameters **dataValues** (*DataContainer instance*) – a dictionary containing the experimental values

Returns **dataArray** – the returned array has a row for each relevant atom in the PDB file. The columns contain model, experimental/calculated data, errors and indexes.

Return type numpy structured array

paramagpy.protein.CustomStructure.set_parent

`CustomStructure.set_parent(entity)`
Set the parent Entity object.

paramagpy.protein.CustomStructure.transform

`CustomStructure.transform(rot, tran)`
Apply rotation and translation to the atomic coordinates.

Parameters

- **rot** (*3x3 Numeric array*) – A right multiplying rotation matrix
- **tran** (*size 3 Numeric array*) – the translation vector

Examples

This is an incomplete but illustrative example:

```
from numpy import pi, array
from Bio.PDB.vectors import Vector, rotmat
rotation = rotmat(pi, Vector(1, 0, 0))
translation = array((0, 0, 1), 'f')
entity.transform(rotation, translation)
```

Attributes

id	Return identifier.

paramagpy.protein.CustomStructure.id

`CustomStructure.id`
Return identifier.

paramagpy.protein.CustomStructureBuilder

class paramagpy.protein.**CustomStructureBuilder**(**arg*, ***kwargs*)
This is an overload hack of BioPython's CustomStructureBuilder

__init__(**arg*, ***kwargs*)
Initialize the class.

Methods

get_structure()	Return the structure.
init_atom(name, coord, b_factor, occupancy, ...)	Create a new Atom object.
init_chain(chain_id)	Create a new Chain object with given id.
init_model(model_id[, serial_num])	Create a new Model object with given id.
init_residue(resname, field, resseq, icode)	Create a new Residue object.
init_seg(segid)	Flag a change in segid.
init_structure(structure_id)	Initialize a new Structure object with given id.
set_anisou(anisou_array)	Set anisotropic B factor of current Atom.
set_header(header)	Set header.
set_line_counter(line_counter)	Tracks line in the PDB file that is being parsed.
set_sigatm(sigatm_array)	Set standard deviation of atom position of current Atom.
set_siguij(siguij_array)	Set standard deviation of anisotropic B factor of current Atom.
set_symmetry(spacegroup, cell)	Set symmetry.

paramagpy.protein.CustomStructureBuilder.get_structure

`CustomStructureBuilder.get_structure()`
Return the structure.

paramagpy.protein.CustomStructureBuilder.init_atom

`CustomStructureBuilder.init_atom`(*name*, *coord*, *b_factor*, *occupancy*, *altloc*, *fullname*, *serial_number=None*, *element=None*)
Create a new Atom object. :param - name - string, atom name, e.g. CA, spaces should be stripped: :param - coord - Numeric array: :type - coord - Numeric array: Float0, size 3 :param - b_factor - float, B factor: :param - occupancy - float: :param - altloc - string, alternative location specifier: :param - fullname - string, atom name including spaces, e.g. " CA ": :param - element - string, upper case, e.g. "HG" for mercury:

paramagpy.protein.CustomStructureBuilder.init_chain

`CustomStructureBuilder.`**`init_chain`**(*chain_id*)

Create a new Chain object with given id.

Parameters **`chain_id – string`** (–) –

paramagpy.protein.CustomStructureBuilder.init_model

`CustomStructureBuilder.`**`init_model`**(*model_id*, *serial_num=None*)

Create a new Model object with given id.

Parameters

- **`id – int`** (–) –
- **`serial_num – int`** (–) –

paramagpy.protein.CustomStructureBuilder.init_residue

`CustomStructureBuilder.`**`init_residue`**(*resname*, *field*, *resseq*, *icode*)

Create a new Residue object.

Parameters

- **`resname – string, e.g. "ASN"`** (–) –
- **`field – hetero flag, "W" for waters, "H" for`** (–) – hetero residues, otherwise blank.
- **`resseq – int, sequence identifier`** (–) –
- **`icode – string, insertion code`** (–) –

paramagpy.protein.CustomStructureBuilder.init_seg

`CustomStructureBuilder.`**`init_seg`**(*segid*)

Flag a change in segid.

Parameters **`segid – string`** (–) –

paramagpy.protein.CustomStructureBuilder.init_structure

`CustomStructureBuilder.`**`init_structure`**(*structure_id*)

Initialize a new Structure object with given id.

Parameters **`id – string`** (–) –

paramagpy.protein.CustomStructureBuilder.set_anisou

`CustomStructureBuilder.`**`set_anisou`**(*anisou_array*)

Set anisotropic B factor of current Atom.

paramagpy.protein.CustomStructureBuilder.set_header

CustomStructureBuilder.**set_header**(*header*)
Set header.

paramagpy.protein.CustomStructureBuilder.set_line_counter

CustomStructureBuilder.**set_line_counter**(*line_counter*)
Tracks line in the PDB file that is being parsed.

Parameters line_counter – int (*-*) –

paramagpy.protein.CustomStructureBuilder.set_sigatm

CustomStructureBuilder.**set_sigatm**(*sigatm_array*)
Set standard deviation of atom position of current Atom.

paramagpy.protein.CustomStructureBuilder.set_siguij

CustomStructureBuilder.**set_siguij**(*siguij_array*)
Set standard deviation of anisotropic B factor of current Atom.

paramagpy.protein.CustomStructureBuilder.set_symmetry

CustomStructureBuilder.**set_symmetry**(*spacegroup*, *cell*)
Set symmetry.

5.6.3 Data I/O module

This module handles the reading and writing of experimental data.

paramagpy.dataparse

Functions

read_pcs(fileName)	Read pseudo contact shift values from file.
read_rdc(fileName)	Read residual dipolar coupling values from file.
read_pre(fileName)	Read paramagnetic relaxation enhancement values from file.
read_ccr(fileName)	Read cross-correlated relaxation values from file.

paramagpy.dataparse.read_pcs

paramagpy.dataparse.**read_pcs**(*fileName*)

Read pseudo contact shift values from file. The returned object is a dicationary. They keys are tuples of (sequence, atomName) The values are tuples of (value, error)

Parameters **fileName** (*str*) – the path to the file

Returns **values** – a dictionary containing the parsed data

Return type *paramagpy.dataparse.DataContainer*

Examples

```
>>> values = paramagpy.dataparse.read_pcs("calbindin_Er_HN_PCS_errors.npc")
>>> for v in values.items():
...     print(v)
...
((2, 'H'), (-0.04855485, 0.0016))
((2, 'N'), (-0.03402764, 0.0009))
((4, 'H'), (-0.18470315, 0.0004))
...
((75, 'H'), (0.19553661, 0.0005))
((75, 'N'), (0.17840666, 0.0004))
```

paramagpy.dataparse.read_rdc

paramagpy.dataparse.**read_rdc**(*fileName*)

Read residual dipolar coupling values from file. The returned object is a dicationary. They keys are frozensets of tuples of the form: frozenset({(sequence1, atomName1), (sequence2, atomName2)}) The frozenset only allows unordered unique atom identification pairs The values are tuples of (value, error)

Parameters **fileName** (*str*) – the path to the file

Returns **values** – a dictionary containing the parsed data

Return type *paramagpy.dataparse.DataContainer*

Examples

```
>>> values = paramagpy.dataparse.read_rdc("ubiquitin_a28c_c1_Tb_HN.rdc")
>>> for v in values.items():
...     print(v)
...
(frozenset({(2, 'N'), (2, 'H')}), (-2.35, 0.32))
(frozenset({(3, 'N'), (3, 'H')}), (-4.05, 0.38))
(frozenset({(4, 'H'), (4, 'N')}), (-3.58, 0.42))
...
(frozenset({(73, 'N'), (73, 'H')}), (-0.47, 0.75))
(frozenset({(76, 'H'), (76, 'N')}), (0.14, 0.3))
```

paramagpy.dataparse.read_pre

paramagpy.dataparse.**read_pre**(*fileName*)

Read paramagnetic relaxation enhancement values from file. The returned object is a dicationary. They keys are tuples of (sequence, atomName) The values are tuples of (value, error)

Parameters **fileName** (*str*) – the path to the file

Returns **values** – a dictionary containing the parsed data

Return type *paramagpy.dataparse.DataContainer*

Examples

see *paramagpy.dataparse.read_pcs()* which has the same file structure

paramagpy.dataparse.read_ccr

paramagpy.dataparse.**read_ccr**(*fileName*)

Read cross-correlated relaxation values from file. These are typically Curie-spin cross Dipole-dipole relaxation rates The returned object is a dicationary. They keys are tuples of the form: ((sequence1, atomName1), (sequence2, atomName2)) Note that the first column is for the active nucleus undergoing relaxation and the second column is for the partner spin. The values are tuples of (value, error)

Parameters **fileName** (*str*) – the path to the file

Returns **values** – a dictionary containing the parsed data

Return type *paramagpy.dataparse.DataContainer*

Examples

see *paramagpy.dataparse.read_rdc()* which has the similar file structure

Classes

DataContainer(*args, **kwargs)	A dictionary-like container for storing PCS, RDC, PRE and CCR data Has an additional attribute 'dtype' to define datatype

paramagpy.dataparse.DataContainer

class paramagpy.dataparse.**DataContainer**(**args*, ***kwargs*)

A dictionary-like container for storing PCS, RDC, PRE and CCR data Has an additional attribute 'dtype' to define datatype

__init__(**args*, ***kwargs*)

Initialize self. See help(type(self)) for accurate signature.

Methods

clear()	
copy()	
fromkeys	Create a new ordered dictionary with keys from iterable and values set to value.
get	Return the value for key if key is in the dictionary, else default.
items()	
keys()	
move_to_end	Move an existing element to the end (or beginning if last is false).
pop(k[,d])	value.
popitem	Remove and return a (key, value) pair from the dictionary.
setdefault	Insert key with a value of default if key is not in the dictionary.
update([E,]**F)	If E is present and has a .keys() method, then does: for k in E: D[k] = E[k] If E is present and lacks a .keys() method, then does: for k, v in E: D[k] = v In either case, this is followed by: for k in F: D[k] = F[k]
values()	

paramagpy.dataparse.DataContainer.clear

`DataContainer.clear()` → None. Remove all items from od.

paramagpy.dataparse.DataContainer.copy

`DataContainer.copy()` → a shallow copy of od

paramagpy.dataparse.DataContainer.fromkeys

`DataContainer.fromkeys()`

Create a new ordered dictionary with keys from iterable and values set to value.

paramagpy.dataparse.DataContainer.get

`DataContainer.get()`

Return the value for key if key is in the dictionary, else default.

paramagpy.dataparse.DataContainer.items

DataContainer.**items**() → a set-like object providing a view on D's items

paramagpy.dataparse.DataContainer.keys

DataContainer.**keys**() → a set-like object providing a view on D's keys

paramagpy.dataparse.DataContainer.move_to_end

DataContainer.**move_to_end**()
Move an existing element to the end (or beginning if last is false).

Raise KeyError if the element does not exist.

paramagpy.dataparse.DataContainer.pop

DataContainer.**pop**(*k*[, *d*]) → v, remove specified key and return the corresponding value. If key is not found, d is returned if given, otherwise KeyError is raised.

paramagpy.dataparse.DataContainer.popitem

DataContainer.**popitem**()
Remove and return a (key, value) pair from the dictionary.

Pairs are returned in LIFO order if last is true or FIFO order if false.

paramagpy.dataparse.DataContainer.setdefault

DataContainer.**setdefault**()
Insert key with a value of default if key is not in the dictionary.

Return the value for key if key is in the dictionary, else default.

paramagpy.dataparse.DataContainer.update

DataContainer.**update**([*E*], ***F*) → None. Update D from dict/iterable E and F.
If E is present and has a .keys() method, then does: for k in E: D[k] = E[k] If E is present and lacks a .keys() method, then does: for k, v in E: D[k] = v In either case, this is followed by: for k in F: D[k] = F[k]

paramagpy.dataparse.DataContainer.values

DataContainer.**values**() → an object providing a view on D's values

5.6.4 Fitting module

This module handles the fitting of paramagnetic objects to experimental data.

paramagpy.fit

paramagpy.fit

Functions

ensemble_average(dataArray)	Calculate the ensemble average for the calculated values in the column 'cal' of the argument <dataArray> over models of the PDB file.
extract_atom_data(data[, csa, separateModels])	Extract values required for PCS/PRE calculations
extract_ccr_data(data[, separateModels])	Extract values required for CCR calculations
extract_rdc_data(data[, separateModels])	Extract values required for RDC calculations
fit_error_bootstrap(fittingFunction, ...)	Perform uncertainty analysis sourcing noise from fractioning the experimental data.
fit_error_models(fittingFunction, **kwargs)	Perform uncertainty analysis sourcing noise from cooridinates as defined by models of the PDB structure.
fit_error_monte_carlo(fittingFunction, ...)	Perform uncertainty analysis sourcing noise from experimental uncertainties This function takes a fitting routine <fittingFunction> and repeats it for the specified iterations in a Monte-Carlo approach.
metal_standard_deviation(metals, params)	Calculate the standard deviation in parameters <params> for a list of metal objects <metals>.
nlr_fit_metal_from_ccr(initMetals, dataArrays)	Fit Chi tensor to CCR values using non-linear regression.
nlr_fit_metal_from_pcs(initMetals, dataArrays)	Fit deltaChi tensor to PCS values using non-linear regression.
nlr_fit_metal_from_pre(initMetals, ...[, ...])	Fit Chi tensor to PRE values using non-linear regression.
qfactor(dataArray[, ensembleAverage, ...])	Calculate the Q-factor to judge tensor fit quality
sphere_grid(origin, radius, points)	Make a grid of cartesian points within a sphere
svd_calc_metal_from_pcs(pos, pcs, idx, errors)	Solve PCS equation by single value decomposition.
svd_calc_metal_from_pcs_offset(pos, pcs, ...)	Solve PCS equation by single value decomposition with offset.
svd_calc_metal_from_rdc(vec, ...)	Solve RDC equation by single value decomposition.
svd_fit_metal_from_rdc(initMetals, dataArrays)	Fit deltaChi tensor to RDC values using Single Value Decomposition.
svd_gridsearch_fit_metal_from_pcs(...[, ...])	Fit deltaChi tensor to PCS values using Single Value Decomposition over a grid of points in a sphere.

paramagpy.fit.ensemble_average

paramagpy.fit.**ensemble_average**(*dataArray*)

Calculate the ensemble average for the calculated values in the column 'cal' of the argument <dataArray> over models of the PDB file. Ensemble averaging behaviour is determined by the column 'idx' of the input array.

Parameters **dataArray** (*numpy array*) – the input array for ensemble averaging

Returns **data** – a smaller dataArray with ensemble averaged values

Return type numpy array

paramagpy.fit.extract_atom_data

paramagpy.fit.**extract_atom_data**(*data*, *csa=False*, *separateModels=True*)

Extract values required for PCS/PRE calculations

Parameters

- **data** (*numpy.ndarray*) – a numpy structured array containing atomic information and experimental data values This is returned from. *paramagpy.protein.CustomStructure.parse()*
- **csa** (*bool, optional*) – when True, calculates the CSA tensor for each atom this may be required for RACS and CSAxDSA calculations
- **separateModels** (*bool, optional*) – when True, separates data into separate lists by their model number. When False, returns only one list

Returns **arr** – this has fields specified by structdtype this array is core to all fitting algorithms

Return type numpy.ndarra

paramagpy.fit.extract_ccr_data

paramagpy.fit.**extract_ccr_data**(*data*, *separateModels=True*)

Extract values required for CCR calculations

Parameters

- **data** (*numpy.ndarray*) – a numpy structured array containing atomic information and experimental data values This is returned from *paramagpy.protein.CustomStructure.parse()*
- **separateModels** (*bool, optional*) – when True, separates data into separate lists by their model number. When False, returns only one list

Returns **arr** – this has fields specified by structdtype this array is core to all fitting algorithms

Return type numpy.ndarra

paramagpy.fit.extract_rdc_data

paramagpy.fit.**extract_rdc_data**(*data*, *separateModels=True*)

Extract values required for RDC calculations

Parameters

- **data** (*numpy.ndarray*) – a numpy structured array containing atomic information and experimental data values This is returned from *paramagpy.protein.CustomStructure.parse()*
- **separateModels** (*bool, optional*) – when True, separates data into separate lists by their model number. When False, returns only one list

Returns **arr** – this has fields specified by structdtype this array is core to all fitting algorithms

Return type numpy.ndarra

paramagpy.fit.fit_error_bootstrap

paramagpy.fit.**fit_error_bootstrap**(*fittingFunction*, *iterations*, *fraction*, ***kwargs*)

Perform uncertainty analysis sourcing noise from fractioning the experimental data. This function takes a fitting routine <fittingFunction> and repeats it for the specified iterations in a Bootstrap approach. With each iteration, a random subset of the experimental data is sampled as specified by the <fraction> argument. The standard deviation in the fitted parameters is then returned.

Parameters

- **fittingFunction** (*function*) – the fitting routine to be used. This could be 'nlr_fit_metal_from_ccr' for example
- **iterations** (*int*) – the number of iterations for the Monte-Carlo simulation
- **kwargs** (*dict*) – all key-word arguments will be bundled into this variable and parsed to the fittingFunction.

Returns

- **sample_metals** (*list of list of metals*) – the metals fitted to the data with noise at each iteration
- **std_metals** (*list of metals*) – the standard deviation in fitted parameters over all iterations of the Monte Carlo simulation. These are stored within the metal object. All unfitted parameters are zero.

paramagpy.fit.fit_error_models

paramagpy.fit.**fit_error_models**(*fittingFunction*, ***kwargs*)

Perform uncertainty analysis sourcing noise from cooridinates as defined by models of the PDB structure. This function takes a fitting routine <fittingFunction> and repeats it for each model. The standard deviation in the fitted parameters is then returned.

Parameters

- **fittingFunction** (*function*) – the fitting routine to be used. This could be 'nlr_fit_metal_from_ccr' for example
- **kwargs** (*dict*) – all key-word arguments will be bundled into this variable and parsed to the fittingFunction.

Returns

- **sample_metals** (*list of list of metals*) – the metals fitted to the data with noise at each iteration

- **std_metals** (*list of metals*) – the standard deviation in fitted parameters over all iterations of the Monte Carlo simulation. These are stored within the metal object. All unfitted parameters are zero.

paramagpy.fit.fit_error_monte_carlo

`paramagpy.fit.`**`fit_error_monte_carlo`**(*fittingFunction*, *iterations*, ***kwargs*)

Perform uncertainty analysis sourcing noise from experimental uncertainties This function takes a fitting routine <fittingFunction> and repeats it for the specified iterations in a Monte-Carlo approach. With each iteration, random noise sourced from a uniform distribution scaled by the experimental uncertainties is added to the experimental values. The standard deviation in the fitted parameters is then returned.

NOTE: the 'err' column of the dataArrays must be set to non-zero values for this method to work.

Parameters

- **fittingFunction** (*function*) – the fitting routine to be used. This could be 'nlr_fit_metal_from_ccr' for example
- **iterations** (*int*) – the number of iterations for the Monte-Carlo simulation
- **kwargs** (*dict*) – all key-word arguments will be bundled into this variable and parsed to the fittingFunction.

Returns

- **sample_metals** (*list of list of metals*) – the metals fitted to the data with noise at each iteration
- **std_metals** (*list of metals*) – the standard deviation in fitted parameters over all iterations of the Monte Carlo simulation. These are stored within the metal object. All unfitted parameters are zero.

paramagpy.fit.metal_standard_deviation

`paramagpy.fit.`**`metal_standard_deviation`**(*metals*, *params*)

Calculate the standard deviation in parameters <params> for a list of metal objects <metals>.

Parameters

- **metals** (*list of Metal objects*) – the metals for which the standard deviation in parameters will be calculated
- **params** (*list of str*) – the parameters for the standard deviation calculation. For example ['x','y','z','ax','rh','a','b','g','shift']

Returns **std_metal** – the returned metal object has attributes equal to the standard deviation in the given parameter. All other attributes are zero.

Return type metal object

paramagpy.fit.nlr_fit_metal_from_ccr

`paramagpy.fit.`**`nlr_fit_metal_from_ccr`**(*initMetals*, *dataArrays*, *params=('x', 'y', 'z')*, *ensembleAverage=False*, *progress=None*)

Fit Chi tensor to CCR values using non-linear regression. This algorithm applies to CSA/Curie spin cross-correlated relaxation for R2 differential line broadening.

Parameters

- **initMetals** (*list of Metal objects*) – a list of metals used as starting points for fitting. a list must always be provided, but may also contain only one element. If multiple metals are provided, each metal is fitted to their respective PRE dataset by index in <dataArrays, but all are fitted to a common position.
- **dataArrays** (*list of PRE dataArray*) – each PRE dataArray must correspond to an associated metal for fitting. each PRE dataArray has structure determined by `paramagpy.protein.CustomStructure.parse()`.
- **params** (*list of str*) – the parameters to be fit. For example ['x','y','z','ax','rh','a','b','g']
- **ensembleAverage** (*bool, optional*) – when False, each model of the structure is fit independently. The parameters for each fitted tensor are then averaged before returning the final averaged tensor. When True, the structure models are treated as an ensemble and ensemble averaging of calculated PCS/PRE/RDC/CCR values is conducted at all stages of fitting to fit a single tensor to all models simultaneously. The 'idx' column of the dataArray determines the ensemble averaging behaviour with common indices for atoms between models resulting in their summation.
- **progress** (*object, optional*) – to keep track of the calculation, progress.set(x) is called each iteration and varies from 0.0 -> 1.0 when the calculation is complete.

Returns

- **fitMetals** (*list of metals*) – a list of the fitted tensors.
- **dataArrays** (*list of dataArray*) – each dataArray is copy of the original dataArray with the 'cal' column populated with back-calculated values from the fitted tensor.

paramagpy.fit.nlr_fit_metal_from_pcs

`paramagpy.fit.`**`nlr_fit_metal_from_pcs`**(*initMetals, dataArrays, params=('x', 'y', 'z', 'ax', 'rh', 'a', 'b', 'g'), ensembleAverage=False, userads=False, useracs=False, progress=None*)

Fit deltaChi tensor to PCS values using non-linear regression.

Parameters

- **initMetals** (*list of Metal objects*) – a list of metals used as starting points for fitting. a list must always be provided, but may also contain only one element. If multiple metals are provided, each metal is fitted to their respective PCS dataset by index in <dataArrays, but all are fitted to a common position.
- **dataArrays** (*list of PCS dataArray*) – each PCS dataArray must correspond to an associated metal for fitting. each PCS dataArray has structure determined by `paramagpy.protein.CustomStructure.parse()`.
- **params** (*list of str*) – the parameters to be fit. For example ['x','y','z','ax','rh','a','b','g','shift']
- **ensembleAverage** (*bool, optional*) – when False, each model of the structure is fit independently. The parameters for each fitted tensor are then averaged before returning the final averaged tensor. When True, the structure models are treated as an ensemble and ensemble averaging of calculated PCS/PRE/RDC/CCR values is conducted at all stages of fitting to fit a single tensor to all models simultaneously. The 'idx' column of the dataArray determines the ensemble averaging behaviour with common indices for atoms between models resulting in their summation.
- **userads** (*bool, optional*) – include residual anisotropic dipolar shielding (RADS) during fitting
- **useracs** (*bool, optional*) – include residual anisotropic chemical shielding (RACS) during fitting. CSA tensors are taken using the <csa> method of atoms.

- **progress** (*object, optional*) – to keep track of the calculation, progress.set(x) is called each iteration and varies from 0.0 -> 1.0 when the calculation is complete.

Returns

- **fitMetals** (*list of metals*) – a list of the fitted tensors.
- **dataArrays** (*list of dataArray*) – each dataArray is copy of the original dataArray with the 'cal' column populated with back-calculated values from the fitted tensor.

paramagpy.fit.nlr_fit_metal_from_pre

paramagpy.fit.**nlr_fit_metal_from_pre**(*initMetals, dataArrays, rtypes, params=('x', 'y', 'z'), usesbm=True, usegsbm=False, usedsa=True, usecsa=False, ensembleAverage=False, progress=None*)

Fit Chi tensor to PRE values using non-linear regression.

Parameters

- **initMetals** (*list of Metal objects*) – a list of metals used as starting points for fitting. a list must always be provided, but may also contain only one element. If multiple metals are provided, each metal is fitted to their respective PRE dataset by index in <dataArrays, but all are fitted to a common position.
- **dataArrays** (*list of PRE dataArray*) – each PRE dataArray must correspond to an associated metal for fitting. each PRE dataArray has structure determined by `paramagpy.protein.CustomStructure.parse()`.
- **rtypes** (*list of str, optional*) – the relaxtion type, either 'r1' or 'r2'. A list must be provided with each element corresponding to an associated dataset. Defaults to 'r2' for all datasets of None is specified.
- **params** (*list of str*) – the parameters to be fit. For example ['x','y','z','ax','rh','a','b','g','shift']
- **usesbm** (*bool, optional*) – include Solomon-Bloemenbergen-Morgan (Dipole-dipole) relaxation theory. default is True
- **usegsbm** (*bool, optional*) – include anisotropic dipolar relaxation theory. note that the g-tensor must be set for this default is False
- **usedsa** (*bool, optional*) – include Dipolar-Shielding-Anisotropy (Curie Spin) relaxation theory. default is True
- **usecsa** (*bool, optional*) – include Chemical-Shift-Anisotropy cross-correlated realxation theory. default is False
- **ensembleAverage** (*bool, optional*) – when False, each model of the structure is fit independently. The parameters for each fitted tensor are then averaged before returning the final averaged tensor. When True, the structure models are treated as an ensemble and ensemble averaging of calculated PCS/PRE/RDC/CCR values is conducted at all stages of fitting to fit a single tensor to all models simultaneously. The 'idx' column of the dataArray determines the ensemble averaging behaviour with common indices for atoms between models resulting in their summation.
- **progress** (*object, optional*) – to keep track of the calculation, progress.set(x) is called each iteration and varies from 0.0 -> 1.0 when the calculation is complete.

Returns

- **fitMetals** (*list of metals*) – a list of the fitted tensors.
- **dataArrays** (*list of dataArray*) – each dataArray is copy of the original dataArray with the 'cal' column populated with back-calculated values from the fitted tensor.

paramagpy.fit.qfactor

paramagpy.fit.**qfactor**(*dataArray*, *ensembleAverage=False*, *calDenominator=False*)

Calculate the Q-factor to judge tensor fit quality

A lower value indicates a better fit. The Q-factor is calculated using the following equation:

$$Q = \sqrt{\frac{\sum_i\left[\left(\sum_m\left[PCS^{exp}_{m,i} - PCS^{calc}_{m,i}\right]\right)^2\right]}{\sum_i\left[\left(\sum_m\left[PCS^{exp}_{m,i}\right]\right)^2\right]}}$$

where m and i are usually indexed over models and atoms respectively.

Parameters

- **dataArray** (*numpy array*) – the dataArray must contain the columns 'exp', 'cal' and 'idx' corresponding to the experimenta, calculated and index values respectively. The index value determines the ensemble averaging behaviour, and can be ignored if the argument <ensembleAverage> is False.
- **ensembleAverage** (*bool, optional*) – when False, the q-factor calculation squares each difference independently. When True, the q-factor calculates an ensemble average before taking the square of differences. The 'idx' column of the dataArray determines the ensemble averaging behaviour with common indices for atoms between models resulting in their summation.
- **calDenominator** (*bool, optional*) – when False, the standard Q-factor is calculated with only the sum of squares for the experimental values used in the denominator when True, the Q-factor established by Ubbink et al. is calculated which has a sum of absolute values of exp and cal values squared in the denominator.

Returns **qfactor** – the Q-factor

Return type float

paramagpy.fit.sphere_grid

paramagpy.fit.**sphere_grid**(*origin*, *radius*, *points*)

Make a grid of cartesian points within a sphere

Parameters

- **origin** (*float*) – the centre of the sphere
- **radius** (*float*) – the radius of the sphere
- **points** (*int*) – the number of points per radius

Returns **array** – the points within the sphere

Return type array of [x,y,z] coordinates

paramagpy.fit.svd_calc_metal_from_pcs

paramagpy.fit.**svd_calc_metal_from_pcs**(*pos*, *pcs*, *idx*, *errors*)

Solve PCS equation by single value decomposition. This function is generally called by higher methods like <svd_gridsearch_fit_metal_from_pcs>

Parameters

- **pos** (*array of [x,y,z] floats*) – the atomic positions in meters
- **pcs** (*array of floats*) – the PCS values in ppm. Note these should be weighted by the experimental uncertainties.

- **idx** (*array of ints*) – an index assigned to each atom. Common indices determine summation between models for ensemble averaging.
- **errors** (*array of floats*) – the standard deviation representing experimental uncertainty in the measured value

Returns

- **calc** (*array of floats*) – the calculated PCS values from the fitted tensor
- **sol** (*array of floats*) – solution to the linearised PCS equation and consists of the tensor 5 matrix elements

paramagpy.fit.svd_calc_metal_from_pcs_offset

paramagpy.fit.**svd_calc_metal_from_pcs_offset**(*pos*, *pcs*, *idx*, *errors*)

Solve PCS equation by single value decomposition with offset. An offset arising from referencing errors between diamagnetic and paramagnetic datasets can be accounted for using this method. This function is generally called by higher methods like <svd_gridsearch_fit_metal_from_pcs>

NOTE: the factor of 1E26 is required for floating point error mitigation

Parameters

- **pos** (*array of [x,y,z] floats*) – the atomic positions in meters
- **pcs** (*array of floats*) – the PCS values in ppm. Note these should be weighted by the experimental uncertainties.
- **idx** (*array of ints*) – an index assigned to each atom. Common indices determine summation between models for ensemble averaging.
- **errors** (*array of floats*) – the standard deviation representing experimental uncertainty in the measured value

Returns

- **calc** (*array of floats*) – the calculated PCS values from the fitted tensor
- **sol** (*array of floats*) – solution to the linearised PCS equation and consists of the tensor 5 matrix elements and offset values

paramagpy.fit.svd_calc_metal_from_rdc

paramagpy.fit.**svd_calc_metal_from_rdc**(*vec*, *rdc_parameterised*, *idx*, *errors*)

Solve RDC equation by single value decomposition. This function is generally called by higher methods like <svd_fit_metal_from_rdc>

Parameters

- **vec** (*array of [x,y,z] floats*) – the internuclear vectors in meters
- **rdc_parameterised** (*array of floats*) – the experimental RDC values, normalised by a prefactor
- **idx** (*array of ints*) – an index assigned to each atom. Common indices determine summation between models for ensemble averaging.
- **errors** (*array of floats*) – the standard deviation representing experimental uncertainty in the measured value

Returns

- **calc** (*array of floats*) – the calculated RDC values from the fitted tensor
- **sol** (*array of floats*) – sol is the solution to the linearised PCS equation and consists of the tensor matrix elements

paramagpy.fit.svd_fit_metal_from_rdc

paramagpy.fit.**svd_fit_metal_from_rdc**(*initMetals*, *dataArrays*, *params=('ax', 'rh', 'a', 'b', 'g')*, *ensembleAverage=False*, *progress=None*)

Fit deltaChi tensor to RDC values using Single Value Decomposition. Note this is a weighted SVD calculation which takes into account experimental errors.

Parameters

- **initMetals** (*list of Metal objects*) – a list of metals used as starting points for fitting. a list must always be provided, but may also contain only one element. If multiple metals are provided, each metal is fitted to their respective RDC dataset by index in <dataArrays>.
- **dataArrays** (*list of PRE dataArray*) – each RDC dataArray must correspond to an associated metal for fitting. each RDC dataArray has structure determined by *paramagpy.protein.CustomStructure.parse()*.
- **params** (*list of str*) – the parameters to be fit. NOTE: This is a dummy argument and does not influence the fitting. The default parameters ('ax','rh','a','b','g') are the only option.
- **ensembleAverage** (*bool, optional*) – when False, each model of the structure is fit independently. The parameters for each fitted tensor are then averaged before returning the final averaged tensor. When True, the structure models are treated as an ensemble and ensemble averaging of calculated PCS/PRE/RDC/CCR values is conducted at all stages of fitting to fit a single tensor to all models simultaneously. The 'idx' column of the dataArray determines the ensemble averaging behaviour with common indices for atoms between models resulting in their summation.
- **progress** (*object, optional*) – to keep track of the calculation, progress.set(x) is called each iteration and varies from 0.0 -> 1.0 when the calculation is complete.

Returns

- **fitMetals** (*list of metals*) – a list of the fitted tensors.
- **dataArrays** (*list of dataArray*) – each dataArray is copy of the original dataArray with the 'cal' column populated with back-calculated values from the fitted tensor.

paramagpy.fit.svd_gridsearch_fit_metal_from_pcs

paramagpy.fit.**svd_gridsearch_fit_metal_from_pcs**(*initMetals*, *dataArrays*, *ensembleAverage=False*, *origin=None*, *radius=20.0*, *points=16*, *offsetShift=False*, *progress=None*)

Fit deltaChi tensor to PCS values using Single Value Decomposition over a grid of points in a sphere. Note this uses a weighted SVD fit which takes into account experimental errors

Parameters

- **initMetals** (*list of Metal objects*) – a list of metals used as starting points for fitting. a list must always be provided, but may also contain only one element. If multiple metals are provided, each metal is fitted to their respective PCS dataset by index in <dataArrays, but all are fitted to a common position.
- **dataArrays** (*list of PCS dataArray*) – each PCS dataArray must correspond to an associated metal for fitting. each PCS dataArray has structure determined by *paramagpy.protein.CustomStructure.parse()*.
- **ensembleAverage** (*bool, optional*) – when False, each model of the structure is fit independently. The parameters for each fitted tensor are then averaged before returning the final averaged tensor. When True, the structure models are treated as

an ensemble and ensemble averaging of calculated PCS/PRE/RDC/CCR values is conducted at all stages of fitting to fit a single tensor to all models simultaneously. The 'idx' column of the dataArray determines the ensemble averaging behaviour with common indices for atoms between models resulting in their summation.

- **origin** (*float, optional*) – the centre of the gridsearch of positions in Angstroms. If None, the position of the first metal is used
- **radius** (*float, optional*) – the radius of the gridsearch in Angstroms.
- **points** (*int, optional*) – the number of points per radius in the gridsearch
- **offsetShift** (*bool, optional*) – if True, an offset value added to all PCS values is included in the SVD fitting. This may arise due to a referencing error between diamagnetic and paramagnetic PCS datasets and may be used when many data points are available. Default False, no offset is included in the fitting.
- **progress** (*object, optional*) – to keep track of the calculation, progress.set(x) is called each iteration and varies from 0.0 -> 1.0 when the calculation is complete.

Returns

- **fitMetals** (*list of metals*) – a list of the fitted tensors.
- **dataArrays** (*list of dataArray*) – each dataArray is copy of the original dataArray with the 'cal' column populated with back-calculated values from the fitted tensor.

PYTHON MODULE INDEX

p

paramagpy.dataparse, 81
paramagpy.fit, 86
paramagpy.metal, 46
paramagpy.protein, 68

INDEX

Symbols

__init__() (paramagpy.dataparse.DataContainer method), 83
__init__() (paramagpy.metal.Metal method), 48
__init__() (paramagpy.protein.CustomAtom method), 69
__init__() (paramagpy.protein.CustomStructure method), 75
__init__() (paramagpy.protein.CustomStructureBuilder method), 79

A

a (paramagpy.metal.Metal attribute), 64
add() (paramagpy.protein.CustomStructure method), 76
alignment_factor (paramagpy.metal.Metal attribute), 64
atom_ccr() (paramagpy.metal.Metal method), 50
atom_pcs() (paramagpy.metal.Metal method), 50
atom_pre() (paramagpy.metal.Metal method), 51
atom_rdc() (paramagpy.metal.Metal method), 51
atom_set_position() (paramagpy.metal.Metal method), 51
average() (paramagpy.metal.Metal method), 51
ax (paramagpy.metal.Metal attribute), 64

B

b (paramagpy.metal.Metal attribute), 65
B0_MHz (paramagpy.metal.Metal attribute), 64

C

ccr() (paramagpy.metal.Metal method), 51
clear() (paramagpy.dataparse.DataContainer method), 84
copy() (paramagpy.dataparse.DataContainer method), 84
copy() (paramagpy.metal.Metal method), 52
copy() (paramagpy.protein.CustomAtom method), 70
copy() (paramagpy.protein.CustomStructure method), 76
csa (paramagpy.protein.CustomAtom attribute), 75
csa_lib (paramagpy.protein.CustomAtom attribute), 75
CustomAtom (class in paramagpy.protein), 69
CustomStructure (class in paramagpy.protein), 75
CustomStructureBuilder (class in paramagpy.protein), 79

D

DataContainer (class in paramagpy.dataparse), 83
detach_child() (paramagpy.protein.CustomStructure method), 76
detach_parent() (paramagpy.protein.CustomAtom method), 70
detach_parent() (paramagpy.protein.CustomStructure method), 76
dipole_shift_tensor() (paramagpy.metal.Metal method), 52
dipole_shift_tensor() (paramagpy.protein.CustomAtom method), 70
dsa_r1() (paramagpy.metal.Metal method), 52
dsa_r2() (paramagpy.metal.Metal method), 52

E

eigenvalues (paramagpy.metal.Metal attribute), 65
ensemble_average() (in module paramagpy.fit), 87
euler_to_matrix() (in module paramagpy.metal), 46
extract_atom_data() (in module paramagpy.fit), 87
extract_ccr_data() (in module paramagpy.fit), 87
extract_rdc_data() (in module paramagpy.fit), 88

F

fast_ccr() (paramagpy.metal.Metal method), 53
fast_dipole_shift_tensor() (paramagpy.metal.Metal method), 53
fast_dsa_r1() (paramagpy.metal.Metal method), 53
fast_dsa_r2() (paramagpy.metal.Metal method), 53
fast_first_invariant_squared() (paramagpy.metal.Metal static method), 54
fast_g_sbm_r1() (paramagpy.metal.Metal method), 54
fast_pcs() (paramagpy.metal.Metal method), 54

fast_pre() (*paramagpy.metal.Metal method*), 54
fast_racs() (*paramagpy.metal.Metal method*), 55
fast_rads() (*paramagpy.metal.Metal method*), 55
fast_rdc() (*paramagpy.metal.Metal method*), 55
fast_sbm_r1() (*paramagpy.metal.Metal method*), 55
fast_sbm_r2() (*paramagpy.metal.Metal method*), 56
fast_second_invariant_squared() (*paramagpy.metal.Metal static method*), 56
first_invariant_squared() (*paramagpy.metal.Metal static method*), 56
fit_error_bootstrap() (*in module paramagpy.fit*), 88
fit_error_models() (*in module paramagpy.fit*), 88
fit_error_monte_carlo() (*in module paramagpy.fit*), 89
fit_scaling (*paramagpy.metal.Metal attribute*), 65
flag_disorder() (*paramagpy.protein.CustomAtom method*), 71
fromkeys() (*paramagpy.dataparse.DataContainer method*), 84
fundamental_attributes (*paramagpy.metal.Metal attribute*), 65

G

g (*paramagpy.metal.Metal attribute*), 65
g_eigenvalues (*paramagpy.metal.Metal attribute*), 65
g_isotropy (*paramagpy.metal.Metal attribute*), 65
g_sbm_r1() (*paramagpy.metal.Metal method*), 56
g_tensor (*paramagpy.metal.Metal attribute*), 65
GAMMA (*paramagpy.metal.Metal attribute*), 64
gax (*paramagpy.metal.Metal attribute*), 65
get() (*paramagpy.dataparse.DataContainer method*), 84
get_altloc() (*paramagpy.protein.CustomAtom method*), 71
get_anisou() (*paramagpy.protein.CustomAtom method*), 71
get_atoms() (*paramagpy.protein.CustomStructure method*), 76
get_bfactor() (*paramagpy.protein.CustomAtom method*), 71
get_chains() (*paramagpy.protein.CustomStructure method*), 76
get_coord() (*paramagpy.protein.CustomAtom method*), 71
get_full_id() (*paramagpy.protein.CustomAtom method*), 71
get_full_id() (*paramagpy.protein.CustomStructure method*), 77
get_fullname() (*paramagpy.protein.CustomAtom method*), 71
get_id() (*paramagpy.protein.CustomAtom method*), 71
get_id() (*paramagpy.protein.CustomStructure method*), 77
get_iterator() (*paramagpy.protein.CustomStructure method*), 77
get_level() (*paramagpy.protein.CustomAtom method*), 72
get_level() (*paramagpy.protein.CustomStructure method*), 77
get_list() (*paramagpy.protein.CustomStructure method*), 77
get_models() (*paramagpy.protein.CustomStructure method*), 77
get_name() (*paramagpy.protein.CustomAtom method*), 72
get_occupancy() (*paramagpy.protein.CustomAtom method*), 72
get_params() (*paramagpy.metal.Metal method*), 57
get_parent() (*paramagpy.protein.CustomAtom method*), 72
get_parent() (*paramagpy.protein.CustomStructure method*), 77
get_residues() (*paramagpy.protein.CustomStructure method*), 78
get_serial_number() (*paramagpy.protein.CustomAtom method*), 72
get_sigatm() (*paramagpy.protein.CustomAtom method*), 72
get_siguij() (*paramagpy.protein.CustomAtom method*), 72
get_structure() (*paramagpy.protein.CustomStructureBuilder method*), 79
get_vector() (*paramagpy.protein.CustomAtom method*), 72
grh (*paramagpy.metal.Metal attribute*), 66
gyro_lib (*paramagpy.protein.CustomAtom attribute*), 75

H

has_id() (*paramagpy.protein.CustomStructure method*), 78
HBAR (*paramagpy.metal.Metal attribute*), 64
HBAR (*paramagpy.protein.CustomAtom attribute*), 75

I

id (*paramagpy.protein.CustomStructure attribute*), 79
info() (*paramagpy.metal.Metal method*), 57
init_atom() (*paramagpy.protein.CustomStructureBuilder method*), 79

init_chain() (*paramagpy.protein.CustomStructureBuilder method*), 80
init_model() (*paramagpy.protein.CustomStructureBuilder method*), 80
init_residue() (*paramagpy.protein.CustomStructureBuilder method*), 80
init_seg() (*paramagpy.protein.CustomStructureBuilder method*), 80
init_structure() (*paramagpy.protein.CustomStructureBuilder method*), 80
insert() (*paramagpy.protein.CustomStructure method*), 78
is_disordered() (*paramagpy.protein.CustomAtom method*), 73
iso (*paramagpy.metal.Metal attribute*), 66
isomap() (*paramagpy.metal.Metal method*), 58
isotropy (*paramagpy.metal.Metal attribute*), 66
items() (*paramagpy.dataparse.DataContainer method*), 85

K

K (*paramagpy.metal.Metal attribute*), 64
keys() (*paramagpy.dataparse.DataContainer method*), 85

L

lanth_axrh (*paramagpy.metal.Metal attribute*), 66
lanth_lib (*paramagpy.metal.Metal attribute*), 66
load_pdb() (*in module paramagpy.protein*), 68
lower_coords (*paramagpy.metal.Metal attribute*), 66

M

make_mesh() (*paramagpy.metal.Metal method*), 58
make_tensor() (*in module paramagpy.metal*), 47
matrix_to_euler() (*in module paramagpy.metal*), 46
Metal (*class in paramagpy.metal*), 48
metal_standard_deviation() (*in module paramagpy.fit*), 89
move_to_end() (*paramagpy.dataparse.DataContainer method*), 85
MU0 (*paramagpy.metal.Metal attribute*), 64
MU0 (*paramagpy.protein.CustomAtom attribute*), 75
MUB (*paramagpy.metal.Metal attribute*), 64

N

nlr_fit_metal_from_ccr() (*in module paramagpy.fit*), 89
nlr_fit_metal_from_pcs() (*in module paramagpy.fit*), 90
nlr_fit_metal_from_pre() (*in module paramagpy.fit*), 91

P

paramagpy.dataparse (*module*), 81
paramagpy.fit (*module*), 86
paramagpy.metal (*module*), 46
paramagpy.protein (*module*), 68
parse() (*paramagpy.protein.CustomStructure method*), 78
pcs() (*paramagpy.metal.Metal method*), 58
pcs_mesh() (*paramagpy.metal.Metal method*), 58
pop() (*paramagpy.dataparse.DataContainer method*), 85
popitem() (*paramagpy.dataparse.DataContainer method*), 85
position (*paramagpy.protein.CustomAtom attribute*), 75
pre() (*paramagpy.metal.Metal method*), 59
pre_mesh() (*paramagpy.metal.Metal method*), 59

Q

qfactor() (*in module paramagpy.fit*), 92

R

racs() (*paramagpy.metal.Metal method*), 59
rads() (*paramagpy.metal.Metal method*), 60
rdc() (*paramagpy.metal.Metal method*), 60
read_ccr() (*in module paramagpy.dataparse*), 83
read_pcs() (*in module paramagpy.dataparse*), 82
read_pre() (*in module paramagpy.dataparse*), 83
read_rdc() (*in module paramagpy.dataparse*), 82
rh (*paramagpy.metal.Metal attribute*), 66
rotation_matrix() (*in module paramagpy.protein*), 69
rotationMatrix (*paramagpy.metal.Metal attribute*), 66

S

saupe_factor (*paramagpy.metal.Metal attribute*), 66
save() (*paramagpy.metal.Metal method*), 60
sbm_r1() (*paramagpy.metal.Metal method*), 60
sbm_r2() (*paramagpy.metal.Metal method*), 60
second_invariant_squared() (*paramagpy.metal.Metal static method*), 61
set_altloc() (*paramagpy.protein.CustomAtom method*), 73
set_anisou() (*paramagpy.protein.CustomAtom method*), 73
set_anisou() (*paramagpy.protein.CustomStructureBuilder method*), 80
set_bfactor() (*paramagpy.protein.CustomAtom method*), 73
set_coord() (*paramagpy.protein.CustomAtom method*), 73

set_header() (*paramagpy.protein.CustomStructureBuilder method*), 81
set_Jg() (*paramagpy.metal.Metal method*), 61
set_lanthanide() (*paramagpy.metal.Metal method*), 61
set_line_counter() (*paramagpy.protein.CustomStructureBuilder method*), 81
set_occupancy() (*paramagpy.protein.CustomAtom method*), 73
set_params() (*paramagpy.metal.Metal method*), 61
set_parent() (*paramagpy.protein.CustomAtom method*), 73
set_parent() (*paramagpy.protein.CustomStructure method*), 78
set_serial_number() (*paramagpy.protein.CustomAtom method*), 73
set_sigatm() (*paramagpy.protein.CustomAtom method*), 74
set_sigatm() (*paramagpy.protein.CustomStructureBuilder method*), 81
set_siguij() (*paramagpy.protein.CustomAtom method*), 74
set_siguij() (*paramagpy.protein.CustomStructureBuilder method*), 81
set_symmetry() (*paramagpy.protein.CustomStructureBuilder method*), 81
set_utr() (*paramagpy.metal.Metal method*), 62
setdefault() (*paramagpy.dataparse.DataContainer method*), 85
spec_dens() (*paramagpy.metal.Metal static method*), 62
sphere_grid() (*in module paramagpy.fit*), 92
svd_calc_metal_from_pcs() (*in module paramagpy.fit*), 92
svd_calc_metal_from_pcs_offset() (*in module paramagpy.fit*), 93
svd_calc_metal_from_rdc() (*in module paramagpy.fit*), 93
svd_fit_metal_from_rdc() (*in module paramagpy.fit*), 94
svd_gridsearch_fit_metal_from_pcs() (*in module paramagpy.fit*), 94

T

tauc (*paramagpy.metal.Metal attribute*), 67
tensor (*paramagpy.metal.Metal attribute*), 67
tensor_alignment (*paramagpy.metal.Metal attribute*), 67
tensor_saupe (*paramagpy.metal.Metal attribute*), 67
tensor_traceless (*paramagpy.metal.Metal attribute*), 67
top() (*paramagpy.protein.CustomAtom method*), 74
transform() (*paramagpy.protein.CustomAtom method*), 74
transform() (*paramagpy.protein.CustomStructure method*), 78

U

unique_eulers() (*in module paramagpy.metal*), 47
update() (*paramagpy.dataparse.DataContainer method*), 85
upper_coords (*paramagpy.metal.Metal attribute*), 67
upper_triang (*paramagpy.metal.Metal attribute*), 67
upper_triang_alignment (*paramagpy.metal.Metal attribute*), 67
upper_triang_saupe (*paramagpy.metal.Metal attribute*), 68

V

values() (*paramagpy.dataparse.DataContainer method*), 85

W

write_isomap() (*paramagpy.metal.Metal method*), 62
write_pymol_script() (*paramagpy.metal.Metal method*), 62

X

x (*paramagpy.metal.Metal attribute*), 68

Y

y (*paramagpy.metal.Metal attribute*), 68

Z

z (*paramagpy.metal.Metal attribute*), 68
